# Circular PCR as an efficient and precise umbrella of methods for the generation of circular dsDNA with staggered nicks: Mechanism and types

**DOI:** 10.1093/biomethods/bpae051

**Published:** 2024-08-12

**Authors:** Pedro Ferro-Gallego, Antón Vila-Sanjurjo, Andrea Katherine Valderrama Pereira, Gonzalo Porres Pérez, Lourdes Domínguez-Gerpe

**Affiliations:** Department of Biochemistry and Molecular Biology, University of Santiago de Compostela, Santiago de Compostela, 15782, Spain; Present Address: IBIMA, Instituto de Investigación Biomédica y Plataforma en Nanomedicina, BIONAND, Málaga, 29590, Spain; Grupo GIBE, Biology Department of the School of Sciences & Interdisciplinary Center for Chemistry and Biology (CICA), Universidade da Coruña (UDC), A Coruña, Spain; Chemistry Department of the School of Sciences & Interdisciplinary Center for Chemistry and Biology (CICA), Universidade da Coruña (UDC), A Coruña, Spain; Grupo GIBE, Biology Department of the School of Sciences & Interdisciplinary Center for Chemistry and Biology (CICA), Universidade da Coruña (UDC), A Coruña, Spain; Department of Biochemistry and Molecular Biology, University of Santiago de Compostela, Santiago de Compostela, 15782, Spain

**Keywords:** CiPCR, seamless cloning, circular-nicked dsDNA, PCR cloning, site-directed mutagenesis, ligation independent cloning

## Abstract

Here, we introduce the highly versatile circular polymerase chain reaction (CiPCR) technique, propose a mechanism of action, and describe a number of examples demonstrating the versatility of this technique. CiPCR takes place between two fragments of dsDNA with two homologous regions, as long as one of the fragments carries said regions at its 3′- and 5′-ends. Upon hybridization, elongation by a polymerase occurs from all 3′-ends continuously until a 5′-end is reached, leading to stable circular dsDNA with staggered nicks. When both dsDNA fragments carry the homology at their 3′- and 5′-ends (Type I CiPCR), all four 3′-ends effectively prime amplification of the intervening region and CiPCR products can function as template during the reaction. In contrast, when only one of the two dsDNA fragments carries the homologous regions at its 3′- and 5′-ends and the other carries such regions internally (Type II CiPCR), only two 3′-ends can be amplified and CiPCR products possess no template activity. We demonstrate the applicability of both CiPCR types via well-illustrated experimental examples. CiPCR is well adapted to the quick resolution of most of the molecular cloning challenges faced by the biology/biomedicine laboratory, including the generation of insertions, deletions, and mutations.

## Introduction

The manipulation of genetic constructs by the introduction of mutations, deletions, tags, as well as by the creation of all types of gene fusions is an everyday task in the modern molecular biology laboratory. Obtaining the desired results quickly while operating under limited budgets requires the development of cheap and highly versatile DNA manipulation technologies. Investing less time and money in obtaining such genetic constructs becomes an obligatory requirement for the advancement of everyday biological and biomedical research.

Since its discovery by K. Mullis in 1985 [1, 2], the polymerase chain reaction (PCR) has undergone a process of continuous modification that improved its versatility, leading to new molecular tools capable of expanding the reach of the PCR technique way beyond the mere amplification of DNA sequences. One of the first applications of PCR was its use in the advancement of DNA cloning methodologies. In the first decade following the start of the genetic engineering revolution, the power of recombinant DNA technologies [3–5] was limited by difficulties related to the availability of appropriate DNA molecules that could be used in cloning schemes. However, the discovery of PCR permitted the amplification of cloning-grade DNA from any natural source that could be used in standard cloning schemes involving restriction enzymes and ligases. Restriction- and ligation-free cloning methods, aimed at simplifying molecular cloning, quickly emerged over the years. These methods were dependent on PCR to different degrees and included T-A cloning [6, 7]; blunt-end cloning [8], ligation-independent cloning (LIC) [9–13], *in vitro* and *in vivo* recombination-based cloning [14–16], Gibson assembly [17], and others [18–22].

**Figure 1.**
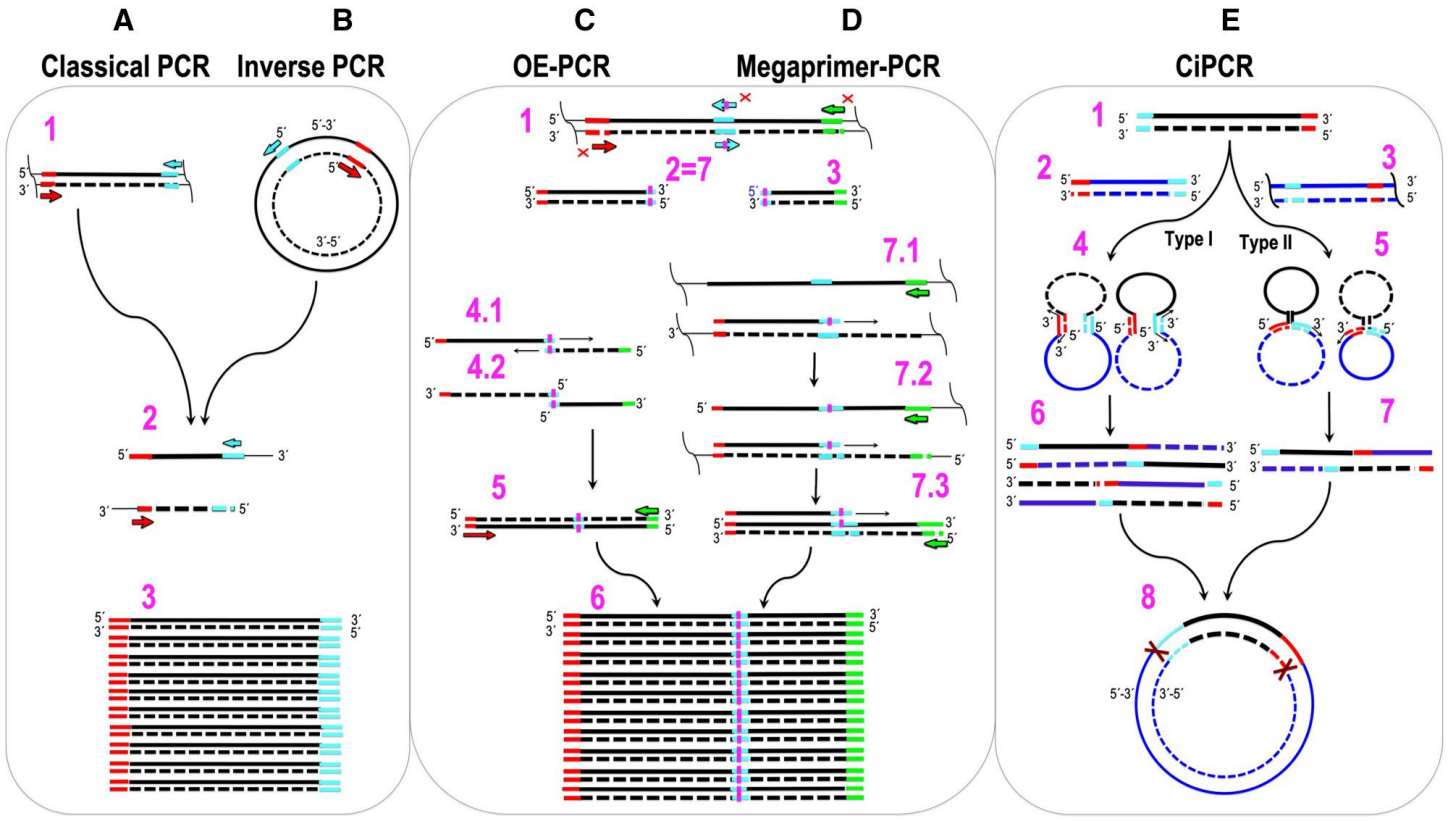
Illustration and comparison of classical PCR, iPCR, OE-PCR, megaprimer-PCR, and CiPCR. (A) Classical PCR. (B) iPCR. Forward primer, right facing arrow; reverse primer, left facing arrow. Homology regions on templates are highlighted next to the primers. Step 1 in (A) and (B), templates and primers. Step 2 in (A) and (B), first-cycle products. Step 3 in (A) and (B), last-cycle products. (C) OE-PCR. (D) Megaprimer-PCR. Step 1 in (C) and (D), templates and primers. Forward primer, left arrow pointing right; reverse primer, right arrow pointing left; mutagenic primers, two central arrows with a vertical stripe. Homology regions on templates are highlighted next to the primers. In (D), only the three primers marked with a cross are used. Steps 2 and 3 in (C), generation of overlapping fragments. Steps 4.1 and 4.2 in (C), overlap extension with strands from steps 2 and 3. Thin arrows indicate extendable 3′-ends. Step 5 in (C), standard PCR with terminal primers. Step 6 in (C) and (D), final product. Step 7 in (D), megaprimer generation. Step 7.1 in (D), generation of first cycle PCR products with megaprimer and reverse primer (indicated in step 1 as right arrow pointing left). Non-productive annealing not shown for simplicity. Step 7.2 in (D), annealing of megaprimer and reverse primer to first-cycle PCR products. Step 7.3 in (D), annealing of megaprimer and reverse primer to second-cycle PCR products. Thin arrows indicate extendable 3′-ends in steps 7.1–7.3. (E) CiPCR. Step 1 in (E), α-DNA (thick central stripe). Steps 2 and 3 in (E), β-DNA in Types I and II CiPCR (thick central stripes). Sites of homology are highlighted as thick 3’- and 5’- stripes. Steps 4 and 5, annealing of α- and β-DNA in Type I (step 4) and II CiPCR (step 5). Thin arrows indicate extendable 3′-ends in steps 4 and 5. Steps 6 and 7 in (E), ssDNA products in Type I (step 6) and II CiPCR (step 7). Step 8 in (E), final CiPCR product consisting of a circular dsDNA molecule with staggered nicks (indicated by crosses). DNA strand in the 5′–3′ direction, solid line, DNA strand in the 3′–5′ direction, broken line. Thin line in step 1 in A and C, and steps 1, 7.1, 7.2 in D indicate DNA regions beyond the priming sites. Wiggly, vertical line indicates undetermined length of the template beyond the priming sites. The position of 5′- and 3′-ends is indicated.

The general mechanisms of classical PCR [1, 2] and later variations, such as inverse (iPCR) [23], overlap extension PCR (OE-PCR) [24, 25], megaprimer-based PCR [26–28], and other adaptations [29–35], are well characterized (Fig. 1). Depending on the polymerase used, PCR-based methods generate dsDNA products that are either blunt-ended [36] or contain a protruding A at their 3′-ends [37, 38]. Classical PCR and iPCR are depicted in Fig. 1A and B, respectively. IPCR applies only to circular templates and is the same as classical PCR, but with the primers extending toward the larger, rather than the smaller arc of the circumference. When the two primers’ binding sites are immediately adjacent, the product is a linear dsDNA molecule of the same length as the circular template (Fig. 1B) [23]. OE-PCR was originally invented to introduce site-directed mutations into linear DNAs [24, 25]. The DNA of interest is split into two fragments by means of a PCR with four primers, two targeted to the terminal regions of the desired fragment (red and green primers in step 1 of Fig. 1C) and two internal, complementary, mutagenic primers (cyan primers in step 1 of Fig. 1C). The overlap between the resulting dsDNA products (steps 2 and 3 in Fig. 1C) permits the extension of the 3′-ends, as shown in step 4.1 in Fig. 1C. The extension product can then be amplified via a regular PCR (step 5 in Fig. 1C). The same final product can be obtained by megaprimer-PCR (Fig. 1D). Instead of linking two DNA fragments, megaprimer-PCR uses one strand of a PCR-amplified dsDNA fragment, which carries the mutation on one of the priming sites, as a long primer (megaprimer), together with a regular primer to amplify the target DNA (step 7.1 of Fig. 1D).

Here, we introduce a new PCR variant named circular PCR (CiPCR) (Fig. 1E). In the results section, we describe this technique on theoretical grounds and delineate its mechanism. Such theoretical considerations led us to propose two mechanisms for CiPCR, Type I (step 4 in Fig. 1E) and Type II (step 5 in Fig. 1E). The theoretical discussion is immediately followed by experiments aimed to prove the validity of the CiPCR hypothesis, namely the demonstration that either Type I or Type II CiPCR can lead to the same result (step 8 in Fig. 1E). A number of practical examples follow, aimed at proving the versatility and simplicity of the CiPCR method, leading to a view that CiPCR can easily be adapted to most molecular cloning applications encountered in the biology/biomedicine laboratory. In the discussion, we present additional mechanistic hypothesis regarding CiPCR kinetics, regarded as an important issue whose elucidation might be required to advance CiPCR-based techniques beyond their use as host-dependent, molecular cloning techniques. The article ends with a discussion of how our definition of CiPCR fits within the myriad of the different molecular cloning methods described in the literature, both PCR- and non-PCR-based. In the context of this discussion, we also provide potential future avenues of research aimed at increasing the palette of CiPCR applications.

## Material and methods

### Biological samples

A 5-mg piece of gut from a control BALB/c mouse was kindly provided by the research group led by Dr M. Freire Rama (Department of Biochemistry and Molecular Biology, USC). Human white blood cells were collected from a blood sample obtained from a patient who signed Informed Consent to participate in a genetic study approved by the Ethics Committee of the local Government of Galicia (Code 2008/137) (Xunta de Galicia, Spain).

### Extraction and retro-transcription of mouse, human, and cell lines total mRNA to obtain cDNA

Total mRNA was extracted with TRIzol^®^ following manufacturer’s instructions from the following sources: BALB/c mouse gut, human white blood cells, or cultured cell lines. Retro-transcription of 1 µg of total mRNA using 170 ng of random hexamers per each sample were performed with M-MLV following manufacturer’s instructions. The resulting products were stored at −20°C.

### Extraction of human gDNA from white blood cells

Genomic DNA (gDNA) was extracted with TRIzol^®^ after mRNA extraction following manufacturer’s instructions.

### Primers, probes, enzymes, especial reagents, genes, tags, plasmids, cell lines, and *bacterial* strains

All the primers and probes used in this study are summarized in Table 1; enzymes and especial reagents in Table 2; genes and tags in Table 3; plasmids in Table 4; eukaryotic cell lines in Table 5. Except when noted otherwise, the *Escheritia coli* strain TG1 was used in all standard chemical transformations and the *E. coli* strain BL21 for protein expression experiments. *Photobacterium damselae* subsp. *piscicola* DI21 was obtained from the laboratory led by Dr M.L. Lemos Ramos (Department of Microbiology and Parasitology, USC).

**Table 1.** Primers and probes used in the experiments.

Name	Sequence
EGFP-1	F: CCAACCCTGAGGAACCAATCACAACCATGGTGAGCAA R: CAATGGCAAGAAAGGCATTACTTGTACAGCTCGTCCATGC
EGFP-2	F: ATGCCTTTCTTGCCATTGTGTGC R: GATTGGTTCCTCAGGGTTGG
EGFP-3	F: CTGCCGGGCTCCCCCAACCCTGAGGAAC R: GGCCCTCTAGAGCACACAATGGCAAG
EGFP-4	F: ATGGTGAGCAAGGGC R: GATTGGTTCCTCAGGGTTGG
Si-SLC16A2	GATCCCCAGCAGCAGCAGCTGCAGCATTCAAGAGATGCTGCAGCTGCTGCTGCTTTTTTA
Si-SLC16A2-1	F: GAAGATGGCTGTGAGGGAC R: CACTCATTAGGCACCCCAG
PCBP1-1	F: CACGATGACGATGACAAGGATGCCGGTGTGACTGAA R: ATGACAACTCCGTCTTCCCTAGCTGCACCCCAT
BRCA1-1	F: TTAGGGCGGAAAGAGTGGGG R: ACGGAAACCAAGGGGCTA
THRB-1	F: GGTCCCGGTGTCACTCGTT R: GCGTCCACCCACCCG
OPRM1-1	F: TTCTCTCCATCTCCCTCCTTTAG R: ACAGAGAGGAGCGTCAGGC
CCAAT-1	F: GAGATATCGGGGGCGGGGCA**CAAACTGGCGGCTGGAC R: TCCAGCCGCCAGTTTG**TGCCCCGCCCCCGATATCTC
SDM-L512P-1	F: TTCTCCATGTCCTTCCCCT R: GATTGGTTCCTCAGGGTTGG
SDM-L512P-2	F: AAAGTACTTCAACATGCGAGTGTT R: AACTCGAGTTAGATTGGTTCCTCAGGGT
SDM-S33P-1	F: GCCCAAGCAGCCACAGTCCCCCCGCCG R: TTGGGCCAGCTTGATCTT
SDM-S33P-2	F: GCCCAAGCAGCCACAGTCCCCCCGCCG R: AGGATCCCGACAGAGTTAT
HA-1	F: AGTCCCCCCGCCGCGATGTACCCATACGATGTTCCAGATTACGCTGCGCTGCAAAGC R: GCTTTGCAGCGCAGCGTAATCTGGAACATCGTATGGGTACATCGCGGCGGGGGGACT
HA-2	F: GCCCAAGCAGCCACAGTCCCCCCGCCG R: TTGCTTCCTCGCTCGCCTGGCTTTGCAGC
HA-3	F: TAATACGACTCACTATAGGG R: TTGCTTCCTCGCTCGCCTGGCTTTGCAGC
HA-4	F: TAATACGACTCACTATAGGG R: CATCGCGGCGGGG
qPCR-TBP	F: CCGAAACGCCGAATATAATCC R: CCGTGGTTCGTGGCTCTCT PROBE: [FAM]CGGTTTGCTGCGGTAATCATGAGGAT[BHQ1]
qPCR-SLC16A2	F: ACTTGCAGGTCCTTTCCTTCCT R: TGATGAAGAAGCCATCGCAAA PROBE: [CY5]CTGATGTCCATGATGATTCCCCTGTGCC[BHQ2]
qPCR-ZAKI-4	F: AGCCTGCCAAACAGTTTCTCA R: TCCCTGCATGGAGCTCATACT PROBE: [HEX]TTCCTCCCCACCTGTTGGCTGG[BHQ1]
frpA-pBAD	F: GGGCTAACAGGAGGAATTAACATGTACAGGAACAGTTTCTCGCTCTCTCC R: CGAATTCCAATGACAACTCCGTCTTCCTTACCACTCTAGCTTCATATTCACACCC
F-pBAD-pelB pelB-His-FrpA-R	F: GGGCTAACAGGAGGAATTAACCATGAAATACCTGCTGCCGACCGCTGCTGCTGGTCTGCTGCTCCTCGCTGCCCAGCCGGCGATGGCC R: CCGTAACTTCTGGATTATGTTCATCATGATGATGATGGTGGTGGTGATGGTGATGGGCCATCGCCGGCTGGGCAGC
Reamp	F: CCCGTTTTTTTGGGCTAACAGGAGGAATTAACCATGAAATACCTGC R:GGTTTCCATAACCGTAACTTCTGGATTATGTTCATC
+1N	GCGGATAACAATTGCACACAAGGAAACACCCNTGAGCAC
Sec_lacZ_R	GATCGCACTCCAGCCAGCTTTCC
PCR-F-SDL	GGCACGACAGGTTTCCCGACTGG

All sequences are written in the 5′–3′-direction. Chimeric primers have two color sequence regions, black is to amplify the insert and blue letters are the sequences with homology to the DNA template. Underlined blue fonts are for extension of homologous regions, ** indicates the position of a deletion. Probes: FAM, fluorescein fluorophore; CY5, cyanine 5 fluorophore; HEX, hexachlorofluorescein fluorophore. BHQ1 and 2, black hole quencher 1 and 2. All primers and probes were from Sigma-Aldrich.

**Table 2.** Enzymes and especial reagents.

Name in the text	Full name	Supplier
BamHI	BamH I Restriction Endonuclease	Roche
Big Dye	Big Dye Terminator v3.1 Cycle Sequencing Kit	4336917, Applied Biosystems
DpnI	Dpn I Restriction Endonuclease	Thermo Fisher Scientific
EcoRI	EcoR I Restriction Endonuclease	Roche
Geneticin	G 418 disulfate salt	Sigma
GST antibody	GST (B14): sc-138	Santa Cruz Biotechnology
Gt-beads	Glutathione Sepharose^®^ 4B	Amersham Biosciences
High Pure PCR Product	High Pure PCR Product Purification kit	Roche
HinfI	Hinf I Restriction Endonuclease	Thermo Fisher Scientific
HpaII	Restriction Endonuclease Hpa II	Roche
HRP-secondary antibody	Amersham ECL Mouse IgG HRP-Linked Whole Ab	Merck
Lipofectamine	Invitrogen™ Lipofectamine™ LTX Reagent	Fisher Scientific
M-MLV	M-MLV Reverse Transcriptase	Invitrogen
Miniprep	GenElute^TM^ HP Plasmid Miniprep Kit	Roche
MunI	Mun I Restriction Endonuclease	Sigma-Aldrich
Ni-beads	Ni-NTA, R901-01,	Invitrogen
Pfu Pol	PfuUltra™ High-Fidelity DNA Polymerase	Stratagene
Phusion Pol	Phusion High-Fidelity DNA Polymerase	Finnzymes
Polyhistidine antibody	Anti-polyhistidine-Peroxidase antibody, Mouse monoclonal	A7058, Sigma-Aldrich
Takara Pol	PrimeSTART™ HS DNA Polymerase	Takara
Taq Pol	Taq DNA Polymerase	Thermo Fisher Scientific
Trypsin	Gibco™ Enzima TrypLE™ Express	Fisher Scientific
XhoI	Xho I Restriction Endonuclease	Roche

**Table 3.** Genes and tags.

Gen	Names	Source
*BRCA1*	BRCA1 DNA repair associated	ENSG00000012048
*frpA*	Bacterial outer membrane iron receptor	Protein accession No. AKQ52529.1, coded by KP100338.1:10003…11985
CI-*lacZ*	cI-lacZ fusion reporter	[39, 40]
*HIST4H4*	H4 HISTONE 16	ENST00000562691
*KLF9*	KRUPPEL-LIKE FACTOR 9	ENSG00000119138
*OPRM1*	opioid receptor mu 1, MOP, MOR1	ENSG00000112038
*PCBP1-201*	Poly(rC) binding protein 1	ENST00000303577
*RCAN2 or ZAKI-4*	Human member 2 of the regulator of calcineurin (RCAN) protein family	ENSG00000172348
*SLC16A2*	Human monocarboxylate transporter 8 (MCT8)	ENSG00000147100
*SP1*	TRANSCRIPTION FACTOR Sp1; SPECIFICITY PROTEIN 1	ENSG00000185591
*TBP*	TATA box binding protein (TBP)	ENST00000230354
*THRB*	Thyroid hormone receptor beta	ENSG00000151090
*Tmprss15*	Mouse enterokinase light chain (mEKLC)	ENSMUST00000023566
HA-tag	Human influenza hemagglutinin aminoacids 98-106	
10H-tag	HHHHHHHHHH polypeptide	
GST-tag	Glutathione S-transferase	
EGFP-tag	Enhanced Green fluorescence protein	
PS target	PreScission protease target sequence	
EK target	Enterokinase target sequence	
PelB signal	MKYLLPTAAAGLLLLAAQPAMA PelB signal sequence	

**Table 4.** Plasmids used or generated in this study.

Plasmid name	Origin
pcDNA3	Invitrogen
pGL4.10(luc2)	Promega
pIRES2-EGFP	Clontech
pSUPER	OligoEngine
pSUPER.neo	OligoEngine
pcDNA3-hSLC16A2(wt) [41]	Gift (see acknowledgements)
pcDNA3-hSLC16A2(wt)-EGFP	This work
pSUPER-Si-hSLC16A2	Unpublished
pSUPER.neo-Si-hSLC16A2	This work
p2GUS-10H-GST-PS-10H-EK-PCBP1(wt)	This work
p2GUS-10H-GST-PS-10H-EK-mTfam(c.1_123del)	Unpublished
pGL4.10(luc2)-hSLC16A2pr(wt)	Unpublished
pGL4.10(luc2)- hSLC16A2pr(pr.-303_-295del)	This work
pGEMTE-hSLC16A2(c.1535T>C)	Unpublished
pcDNA3-hSLC16A2(c.1535T>C)	This work
pcDNA3-hSLC16A2 (c.97T>C)	This work
pcDNA3-HA-hSLC16A2(wt)	This work
pBAD	[42]
pBAD-FrpA(wt)	[43]
pBAD-pelB-10XHis-FrpA	[43]
SDL	[55, 56]
SDL-+1 series of plasmids	This work

**Table 5.** Cell lines.

Cell lines		
Name	Description	Source
A-172	Human glioblastoma cell line	ATCC^®^ CRL-1620
COS-7	African green monkey kidney fibroblast-like cell line	ATCC^®^ CRL-1651™
JEG-3	Human Choriocarcinoma cell line	ATCC^®^ HTB-36™
NCI H-460	Human lung cancer cell line	ATCC^®^ HTB-177™
SH-SY5Y	Human neuroblastoma cell line	ATCC^®^ CRL-2266™

### DsDNA cloning, mutagenesis, or deletion by CiPCR

To keep track of all PCR-based reactions, they will be given a name in this section that will be used in all throughout the article.

1. *PCR1. Amplification of an insert carrying the sequence of EGFP.* The 763-bp insert was created via a standard PCR in which the pair of chimeric primers EGFP-1 were used to amplify the enhanced green fluorescence protein (EGFP) from pIRES2-EGFP. A 50 µl PCR was prepared that contained the manufacturer’s recommended buffer; primers (0.2 µM), dNTPs (0.2 mM), 100 ng of pIRES2-EGFP, and 0.5 U of Takara Pol. Amplification was performed with 1 denaturation cycle at 98°C (40 s); 35 amplification cycles composed of the following three stages, 98°C (10 s), 62°C (40 s), and 72°C (60 s); and 1 final cycle at 72°C (10 min). PCR products were precipitated with sodium acetate-ethanol and then dissolved in water.
2. *iPCR1. Amplification of a vector carrying the sequence of pcDNA3-hSLC16A2(wt).* A 7070-bp linear fragment was amplified via inverse PCR (iPCR) [23] with pcDNA3-hSLC16A2(wt) [41] as a template and with the pair of primers EGFP-2. To do this, a 50 µl iPCR was prepared that contained the manufacturer’s recommended buffer; primers (0.2 µM), dNTPs (0.2 mM), 100 ng of pcDNA3-hSLC16A2(wt); and 0.5 U of Takara Pol. Amplification was performed with 1 denaturation cycle at 98°C (40 s); 40 amplification cycles composed of the following three stages, 98°C (10 s), 63°C (30 s), and 72°C (8 min); and 1 final cycle at 72°C (15 min). The PCR products were treated with 2 U of DpnI during 6 h at 37°C and stored at −20°C without further purification.
3. *CiPCR1 for cloning (EGFP in pcDNA3-hSLC16A2(wt)).* A 25-µl, Type I CiPCR was prepared that contained the manufacturer’s recommended buffer; dNTPs (0.2 mM), 250 ng of linear vector and insert DNA from PCR1 and iPCR1 above; and 1.25 U of Takara Pol. Amplification was performed with 1 denaturation cycle at 98°C (40 s); 20 amplification cycles composed of the following three stages, 98°C (10 s), 63°C (30 s), and 72°C (8 min); and 1 final cycle at 72°C (15 min). PCR products were precipitated with sodium acetate-ethanol and then dissolved in water. Following transformation, transformants were directly screened for the presence of the final construct, pcDNA3-hSLC16A2(wt)-EGFP, by means of colony-PCR with the pair of primers EGFP-3. The integrity of the resulting pcDNA3-hSLC16A2(wt)-EGFP was also verified by restriction analysis and sequencing.
4. *CiPCR2 for direct cloning (EGFP in pcDNA3-hSLC16A2(wt)).* A 25-µl, Type II CiPCR was prepared that contained the manufacturer’s recommended buffer; dNTPs (0.2 mM); 500 ng of the 763-bp EGFP insert from PCR1 above; 100 ng of pcDNA3-hSLC16A2(wt); and 1.25 U of Takara Pol. Amplification was performed with 1 denaturation cycle at 95°C (5 min); 7 amplification cycles composed of the following three stages, 98°C (30 s), 59°C (2 min), 72°C (8 min); 12 amplification cycles composed of the following three stages, 98°C (20 s), 85°C (1 min), 72°C (8 min); and 1 final cycle at 72°C (15 min), followed by addition of 4 U of DpnI to the CiPCR mixture in the same PCR tube, incubation at 37°C for 1 h and an inactivation step at 65°C for 15 min. Following chemical transformation, transformants were directly screened for the presence of the final construct, pcDNA3-hSLC16A2(wt)-EGFP, by means of colony-PCR with the pair of primers EGFP-3, and by sequencing.
5. *iPCR2. Analysis of the potential presence of CiPCR byproduts.* IPCR2 was used to verify the integrity of the CiPCR2 products as well as the potential presence of unnicked-byproducts with lengths longer than those of the predicted products. A 20-µl iPCR2 was prepared that contained the manufacturer’s recommended buffer, the pair of primers EGFP-4 (0.2 µM), dNTPs (0.2 mM), 1 µl CiPCR product or 5 ng of pure pcDNA-hSLC16A2(wt)-EGFP as templates, and 1.25 U of Takara Pol to obtain a 7789-bp DNA product. Amplification was performed with 1 denaturation cycle at 98°C (40 s); 30 amplification cycles composed of the following three stages, 98°C (10 s), 58°C (30 s), and 72°C (8 min); and 1 final cycle at 72°C (10 min). The results were analyzed by agarose gel electrophoresis.
6. *PCR2. Amplification of a SiRNA precursor.* A 389-bp dsDNA containing a 60-bp SiRNA precursor (Si-*SLC16A2*, Table 1), was PCR amplified with the pair of primers Si-*SLC16A2*-1 and a template composed of pSUPER-Si-hSLC16A2. A 40-µl PCR was prepared that contained the manufacturer’s recommended buffer, dNTPs (0.2 mM), primers (0.2 µM), 1 µl pSUPER-Si-hSLC16A2, and 1.25 U of Takara Pol. Amplification was performed with 1 denaturation cycle at 94°C (30 s); 30 amplification cycles composed of the following three stages, 98°C (10 s), 55°C (15 s), 72°C (30 s); and 1 final cycle at 72°C (10 min). The insert was kit-purified and stored at −20°C.
7. *CiPCR3 for direct cloning (SiRNA).* A 25-µl, Type II CiPCR was prepared that contained the manufacturer’s recommended buffer, dNTPs (0.2 mM), 250 ng of the insert from PCR2, 100 ng of pSUPER.neo, and 1.25 U of Takara Pol. Amplification was performed with 1 denaturation cycle at 95°C (1 min); 18 amplification cycles composed of the following four stages, 98°C (20 s), 85°C (20 s), at 60°C (20 s), 72°C (7 min); and 1 final cycle at 72°C (10 min). Following treatment with 2 U of DpnI for 2 h at 37°C and *E. coli* transformation with the CiPCR products, 1 colony was grown. The integrity of the resulting plasmid, pSUPER.neo-Si-hSLC16A2, was verified by restriction analysis with the enzymes EcoRI and XhoI as well as by DNA sequencing.
8. *PCR3. Amplification of an insert carrying the sequence of hPCBP1(wt).* A 50-µl PCR was prepared that contained the manufacturer’s recommended buffer; PCBP1-1 primers (0.2 µM), dNTPs (0.2 mM), 1 µl of the cDNA obtained from human white blood cells (see above); and 1.25 U of Takara Pol. Amplification was performed with 1 denaturation cycle at 94°C (40 s); 40 amplification cycles composed of the following three stages, 98°C (10 s), 60°C (30 s), 72°C (60 s); and 1 final cycle at 72°C (10 min). PCR products were kit-purified and stored at −20°C.
9. *CiPCR4 for direct cloning hPCBP1(wt) in p2GUS-10H-GST-PS-10H-EK-mTAM(c.1_123del).* A 25-µl, Type II CiPCR was prepared that contained the manufacturer’s recommended buffer, dNTPs (0.2 mM), 250 ng of the insert from PCR3, 100 ng of p2GUS-10H-GST-PS-10H-EK-mTfam(c.1_123del), and 1.25 U of Takara Pol. Amplification was performed with 1 denaturation cycle at 95°C (5 min); 5 cycles composed of the following four stages, 95°C (1 min), 65°C (30 s), 62°C (30 s), and 72°C (7 min); 11 cycles composed of the following three stages: 98°C (20 s), 80°C (15 s), 63°C (15 s), and 72°C (7 min); and 1 final cycle at 72°C (15 min). PCR products were treated with 2 U of DpnI for 2 h at 37°C prior to ethanol precipitation and final resuspension in water. Screening of transformants by colony-PCR was performed with the same pair of primers PCBP1-1. The correct insertion of *hPCBP1(wt)* ORF in the resulting plasmid, p2GUS-10H-GST-PS-10H-EK-hPCBP1(wt), was verified by sequencing.
10. *CiPCR5 for the generation of deletions.* A 50-µl, Type II PCR was prepared that contained the manufacturer’s recommended buffer, dNTPs (0.2 mM), the pair of chimeric primers CCAAT-1 (0.2 µM), 60 ng of pGL4.10(luc2)-hSLC16A2pr(wt); and 1.25 U of Takara Pol. Amplification was performed with 1 denaturation cycle at 95°C (30 s); 18 amplification cycles composed of the following three stages, 98°C (10 s), 80°C (15 s), 72°C (8 min); and 1 final cycle at 72°C (15 min), followed by treatment with 4 U of DpnI at 37°C for 2 h, and an inactivation step at 65°C for 15 min. The presence of the deletion in the resulting plasmid, pGL4.10(luc2)-hSLC16A2pr(pr.-993_-985del), was verified by sequencing analysis.
11. *PCR4. Generating a mutagenic insert (hSLC16A2(c1535T>C)).* An 894-bp mutagenic insert carrying the *hSLC16A2(c1535T>C)* transition was generated with the pair of primers SDM-L512P-1 and pGEMTE-hSLC16A2(c1535T>C) as a template. A 50-µl PCR was prepared that contained the manufacturer’s recommended buffer, primers (0.2 µM), dNTPs (0.2 mM), 1 µl of pGEMTE-hSLC16A2(c1535T>C), and 1.25 U of Pfu Pol. Amplification was performed with 1 denaturation cycle at 94°C (40 s); 30 amplification cycles composed of the following three stages, 94°C (20 s), 59°C (30 s), 72°C (60 s); and 1 final cycle at 72°C (10 min). The dsDNA insert was purified with a PCR Purification kit and stored at −20°C.
12. *CiPCR6 for site-directed mutagenesis (hSLC16A2(c1535T>C).* A 30-µl, Type II CiPCR was set up that contained the manufacturer’s recommended buffer, dNTPs (0.2 mM), 250 ng of the 894-bp insert from PCR4, 100 ng of pcDNA3-hSLC16A2(wt), 0.8 µl DMSO, and 1.25U of Pfu Pol. Amplification was performed with 1 denaturation cycle at 95°C (60 s); 18 amplification cycles composed of the following three stages, 95°C (60 s), 74°C (30 s), 72°C (5 min); and 1 final cycle at 72°C (15 min). Following treatment with 2 U of DpnI for 1 h at 37°C, the products were ethanol precipitated and resuspended in water. Following transformation, colony-PCR screening of transformants carrying the newly made pcDNA3- hSLC16A2(c1535T>C) was performed with the pair of primers SDM-L512P-2. The presence of the mutation was verified by restriction of these PCR fragments with HpaII and by sequencing.
13. *PCR5. Generating a mutagenic insert (hSLC16A2(c.97T>C)).* The protocol was the same as in PCR4 but with the pair of primers SDM-S33P-1 and a template composed of cDNA from a patient that carried the rs6647476 allelic variant.
14. *CiPCR7 for site-directed mutagenesis (hSLC16A2(c.97T>C)).* The protocol was the same as in CiPCR6 above, but with the 812-bp insert generated in PCR5. Verification was performed by colony-PCR screening with the pair of primers SDM-S33P-1 followed by restriction analysis with the enzyme HinfI, and by sequencing.
15. *Enlargement of terminal homology regions.* This procedure required the following steps: PCR7, PCR8, and OE-PCR1 for CiPCR8, and a single cycle extension and PCR10 for CiPCR10 (see below).
16. *PCR7. Generation of α-DNA_2_.* A 90-bp dsDNA fragment containing the 27-bp HA epitope was obtained by PCR7 with the pair of primers HA-2 and a template composed of the oligonucleotide HA-1-F (57-mer, Table 1). A 50-µl PCR7 was prepared that contained the manufacturer’s recommended buffer, dNTPs (0.2 mM), primers HA-2 (0.2 µM), 50 ng of the HA-1-F template, and 1.25 U of Takara Pol. Amplification was performed with 1 denaturation cycle at 94°C (40 s); 40 amplification cycles composed of the following three stages, 98°C (10 s), 58°C (30 s), 72°C (30 s); and 1 final cycle at 72°C (10 min). The insert was kit-purified and stored at −20°C. The 90-bp dsDNA fragment was used in PCR8, below.
17. *PCR8. Generation of 107-bp DNA fragments for the enlargement of α-DNA_2_*. A 107-bp dsDNA fragment was obtained with the pair of primers HA-4 and a template composed of pcDNA3-hSLC16A2 (wt) by PCR8. A 50-µl reaction was prepared that contained the manufacturer’s recommended buffer; dNTPs (0.2 mM); primers HA-4 (0.2 µM); 100 ng of template; and 1.25 U of Takara Pol. Amplification was performed with 1 denaturation cycle at 98°C (40 s); 40 amplification cycles composed of the following three stages, 98°C (10 s), 58°C (30 s), 72°C (30 s); and 1 final cycle at 72°C (10 min). The insert was kit-purified and stored at −20°C. The 107-bp dsDNA fragment was used in OE-PCR1, below.
18. *OE-PCR1. Generation of* α-*DNA_3_.* The overlap between the 90- and 107-bp fragments was used in the OE-PCR1 to generate a 165-bp insert. A 50-µl reaction was prepared that contained the manufacturer’s recommended buffer; dNTPs (0.2 mM); the pair of primers HA-3 (0.2 µM); 100 ng of each of the purified DNA from PCR7 and PCR8; and 1.25 U of Takara Pol to obtain a 165-bp dsDNA fragment. Amplification was performed with 1 denaturation cycle at 94°C (40 s); 40 amplification cycles composed of the following three stages, 98°C (10 s), 58°C (30 s), 72°C (30 s); and 1 final cycle at 72°C (10 min). PCR products were kit-purified and stored at −20°C.
19. *CiPCR8. Type II CiPCR for the construction of* pcDNA3-HA-hSLC16A2(wt)*.* The 165-bp insert created by OE-PCR1 was used in a Type II CiPCR. A 25-µl Type II CiPCR was prepared that contained the manufacturer’s recommended buffer, dNTPs (0.2 mM); 250 ng of the insert from OE-PCR1; 100 ng of pcDNA3-hSLC16A2(wt); and 1.25 U of Takara Pol. Amplification was performed with 1 denaturation cycle at 95°C (5 min); 6 cycles composed of the following four stages, 98°C (1 min), 74°C (90 s), 63°C (30 s), and 72°C (7 min); 10 cycles composed of the following three stages: 98°C (20 s), 74°C (15 s), 63°C (15 s), and 72°C (7 min); and 1 final cycle at 72°C (15 min). CiPCR products were treated with 2 U of DpnI for 2 h at 37°C prior to ethanol precipitation and *E. coli* transformation. Colony-PCR screening of transformants carrying the newly made pcDNA3-HA-hSLC16A2(wt) was performed with the pair of primers HA-3 and verified by sequencing analysis.
20. *PCR9 for the generation of an α-DNA carrying the sequence of frpA(wt).* The template for this PCR was a suspension of gDNA obtained after the resuspension of a colony of *P. damselae* subsp. *piscicola* DI21 in 100 µl of ultrapure water, followed by incubation at 96°C for 10 min with slow agitation. A 25-µl PCR was set up that contained the manufacturer’s recommended buffer; dNTPs (0.2 mM), MgCl_2 _(1mM); the pair of primers frpA-pBAD (1.25 µM); 1 μl of template; and 0.5 U of Phusion Pol. Amplification was performed with 1 cycle of 98°C (30 s); 30 cycles with the following stages: 98°C (30 s), 60°C (30 s), and 72°C (3 min); 1 cycle at 72°C (10 min).
21. *CiPCR9 type II for the cloning of frpA(wt) in pBAD.* A 25-μl Type II CiPCR containing the manufacturer’s recommended buffer; dNTPs (0.2 mM), MgCl_2_ (1mM); 0.5 μl of a miniprep of pBAD as β-DNA; 12.5 μl of kit-purified α-DNA from PCR9 above, and 0.5 U of Phusion Pol. The annealing temperatures for the forward and reverse regions of homology between α-DNA and β-DNA (T_F_ and T_R_) were calculated with IDT SciTools web server [44]. Amplification was performed with 1 cycle of denaturation at 95°C (5 min); 20 cycles with the following four steps: 95°C (1 min), higher of T_F_ and T_R_ (30 s), lower of T_F_ and T_R_ (30 s); 72°C (1 min per kb of β-DNA); 10 cycles with the following 3 steps: 98°C (20 s), 80°C (15 s), and 72°C (1 min per kb of β-DNA); 1 cycle at 72°C for 15 min. Following DpnI treatment, the CiPCR products were transformed and colonies carrying the desired product were identified by colony-PCR.
22. *Insertion of PelB-10H with simultaneous deletion of frpA(wt)’s signal sequence.* This procedure required the following steps: single cycle extension and PCR10 as follows. First, FrpA(wt)’s signal sequence was identified by SignalP 4.1 Server N [45]. Primers F-pBAD-pelB and pelB-His-FrpA-R were designed and analyzed with the IDT SciTools web server [44]. Following chemical synthesis, the primers were HPLC-purified by manufacturer (Sigma-Aldrich). An aliquot of 150 μl of a single-cycle extension reaction to create the desired α-DNA (143 nts), was prepared that contained the manufacturer’s recommendation buffer, the above pair of primers (3.33 μM); dNTPs (0.4 mM); and MgCl_2_ (1 mM). The mixture was separated into three vials, two of which received 1 U of Phusion pol each, whereas the other was left as a no-polymerase control. A single cycle of extensión at 72°C (30 s) was applied following denaturation and annealing.
23. *PCR10 for the amplification of the single-cycle extension products.* The unpurified products of above extension were used directly as a template in a PCR with primers Reamp following manufacturer’s conditions for Phusion pol. The expected 165-nt product was run on a 2.5% agarose gel, the band was cut, kit-purified, and stored for later use.
24. *CiPCR10 to generate pBAD-pelB-10XHis-FrpA*. This CiPCR was setup just like CiPCR9 but with the above PCR10-165-nt product as α-DNA and pBAD-FrpA(wt) as β-DNA.
25. *PCR11 for the amplification of the α-DNA carrying a reporter gene based on lacZ.* The target α-DNA (308-bp) contained the start codon of the cI-*lacZ* fusion carried on plasmid SDL. The pair of oligonucleotides was composed of the forward mutagenic primer +1N and the reverse primer Sec-lacZ-R (Table 1), the latter binding 255 nts downstream of the first base of cI-*lacZ’*s start codon. A 25-µl PCR was set up that contained the manufacturer’s recommended buffer; dNTPs (0.2 mM), MgCl_2_ (1 mM); the above pair of primers (0.8 µM); a template composed of 1 μl from a miniprep of plasmid SDL; and 0.5 U of Phusion Pol. Amplification was performed with 1 cycle of 98°C (30 s); 30 cycles with the following stages: 98°C (30 s), 62.5°C (30 s), and 72°C (1 min); 1 cycle at 72°C (10 min).
26. *CiPCR11 for the creation of the SDL-+1 series of plasmids by randomized SDM.* Performed as CiPCR9 with the α-DNA created in PCR11 and plasmid SDL as β-DNA. Following DpnI digest and transformation, colony-PCR analysis of the transformants with primers PCR-F-SDL, whose binding site starts 160 nts from the first base of cI-*lacZ’*s start codon and Sec-lacZ-R. The 468-bp-colony-PCR generated products were digested with MunI to detect the mutant constructs via electrophoresis analysis. The presence of mutations at position +1 was verified by DNA sequencing analysis.

#### Expression of genes by green fluorescent reporters

The human A-172 and NCI H-460 cell lines, that are adherent cells growing attached to the wells, were cultured following provider’s instructions. Cells were collected at confluence and transferred at a concentration of 2x10^5^ cells/well to a 24-well plate that contained plastic coverslips (Nunc™ Thermanox™, ThermoFisher) in the bottom. The coverslips are used to facilitate their removal from the well for its direct mounting over a normal microscope slide to perform the microscopic observations. After 19 h, cells were washed with PBS and fresh medium was added. Five hours later, cells were transfected with pcDNA3-SLC16A2(wt)-EGFP (obtained by CiPCR2) (500 µg) by means of Lipofectamine following manufacturer’s instructions. At 12 h post-transfection, the cells were washed with PBS and fresh medium was added. An additional wash was performed at 48 h post-transfection, after which the cells were fixed with 4% paraformaldehyde and the coverslips, containing the cells, were collected and mounted on microscope slides with Fluorescence Mounting Medium containing 100 µg/l of DAPI (Dako). A-172 cells were analyzed with the Olympus BX51 and Nikon Eclipse E1000 fluorescence microscopes, whereas NIH H-460 cells were monitored with a Leica TCS-SP2 confocal microscope.

#### Gene-silencing capacity of the Si-RNA-expressing constructs

The human SH-SY5Y cell line was cultured following provider’s instructions. Cells were collected at confluence and distributed into four wells in a 24-well plate. When 80% confluence was reached, cells were washed with PBS and fresh medium was added. After 5 h, the medium was replaced again, prior to lipofectamine transfection of two wells with pSUPER.neo-Si-SLC16A2 (500 µg) and the other two wells with pSUPER.neo, following manufacturer’s instructions. At 12 h post-transfection, the cells were washed with PBS and fresh medium was added. At 24 h post-transfection geneticin (400 µg/ml) was added and the medium was replaced every 2–3 days maintaining geneticin selection. On day 8 post-transfection, cells were washed with PBS, trypsinized, transferred to Eppendorf tubes and pelleted by centrifugation. *SLC16A2* and *ZAKI-4* gene expression was quantified by triplex qPCR using the Real-Time thermocycler Mx3005P (Stratagene) and the *TBP* housekeeping gene as reference. To do this, 25-µl reactions (triplicate) were prepared that contained 12.5 µl of FastStart Universal Probe Master (ROX) (Roche); 1 µl of cDNA obtained by retro-transcription of 50 ng of total RNA as described above; primers qPCR-*TBP*, qPCR-*SLC16A2*, and qPCR-*ZAKI-4* (0.1 µM each); probes qPCR-*TBP*, qPCR*-SLC16A2*, and qPCR-*ZAKI-4* (0.2 µM each). Amplification was performed with 1 denaturation cycle at 95°C (10 min); and 50 amplification cycles composed of the following two stages, at 95°C (15 s) and 60°C (60 s). The results were analyzed following the mathematical model 2^−ΔΔCT^ [46].

#### Small-scale expression of recombinant (r) -10H-GST-PS-10H-EK-hPCBP1(wt)

*Escherichia coli* BL21 cells carrying p2GUS-10H-GST-PS-10H-EK-hPCBP1(wt) were grown in 100 ml of LB culture medium supplemented with ampicillin (50 µg/ml) up to an OD_600_ of 0.4 U. To activate expression, arabinose (Sigma-Aldrich) was added to a concentration of 0.2% (w/v). The 1-ml samples were collected hourly for 6 h and their OD_600_ was measured. Following centrifugation of all the collected samples, the bacterial pellets were stored at −80°C. Pellets were thawed on ice and the volume of ice-cold lysis buffer (50 mM Tris–HCl pH 7.9, 0.5 M NaCl, 1 µg/ml leupeptin, 1 mM PMSF, and 1 µg/ml aprotinin) used for pellet resuspension was normalized to their associated OD_600_ value, starting with 100 µl in the case of the zero time-point control. The resuspended samples were sonicated with a 150 V/T Ultrasonic Homogenizer (Biologics, Inc.) operated at 50% of its capacity. Following four 4-s sonication pulses with a 5-s ice-cooling interval between pulses, we further lysed the cells with three cycles of freeze–thaw performed by alternating incubation in dry-ice and regular ice. All samples were centrifuged at 15,000 × *g* for 20 min at 4°C to remove debris and obtain r-10H-GST-PS-10H-EK-hPCBP1(wt)-rich supernatants.

#### Western blot analysis of r-10H-GST-PS-10H-EK-hPCBP1(wt)

R-10H-GST-PS-10H-EK-hPCBP1(wt)-rich supernatants were loaded onto 12% SDS–PAGE polyacrylamide minigels (Mini-Protean 3, BioRad) and, following electrophoresis, transferred to an activated PVDF membrane (Hybond-P, RPN303F, Amersham Life Sciences) with the Mini Trans-Bloot^®^ (Bio-Rad) at 60 V for 2 h. The PVDF membrane was washed with 0.05% tween-20 in PBS; incubated overnight with 0.05% tween-20 and 10% powder-milk in PBS at RT and slow orbital stirring; washed 3 times with 0.05% tween 20 in PBS; and incubated with 5 ml of a 1:2.500 dilution of a primary polyhistidine or GST antibody for 1 h at room temperature and slow orbital stirring. This was followed by three additional washes with 0.05% tween-20 in PBS and then incubation with 5 ml of a 1: 5000 dilution of an HRP-conjugated, mouse secondary antibody for 1 h at room temperature and slow orbital stirring. After 3 additional washes with 0.05% tween 20 in PBS, the membrane was reacted with Lumigen (Lumigen™ PS-3, Amersham) following manufacturer’s instructions, and used to expose a Hyperfilm (ECL, 108531A, Amersham).

#### Large-scale expression and purification of tagged r-10H-GST-PS-10H-EK-hPCBP1(wt)

For large-scale (150 ml) production of r-10H-GST-PS-10H-EK-hPCBP1(wt) cultures were stopped when they reached an OD_600_ of 0.4, at which point 0.2% arabinose was added. After incubation for 3 h at 37°C with continuous shaking at 200 rpm, the culture was chilled on ice prior to centrifugation at 3000 × *g* for 20 min at 4°C. The bacterial pellet was resuspended in 5 ml of lysis buffer, and lysed as described above, but with 10 sonication pulses instead of 4. The supernatant was incubated with 200 µl of Ni-beads (Ni-NTA, R901-01, Invitrogen) following by 2 h of gently orbital shaking at 4°C, 2 washes with lysis buffer and 2 with lysis buffer that contained 50 mM imidazole. Following 5 min incubation the protein was eluted by adding 200 µl of lysis buffer that contained 1M imidazole, and following centrifugation the supernatant was collected. The elution step was repeated again, and the supernatant was transferred to the same tube. Then, 150 µl of Gt-beads (Glutathione Sepharose 4B beads, Amersham) were added to the supernatant, following by 2 h of gently agitation in an orbital shaker, the beads were pelleted and then washed 2 times with cold lysis buffer supplemented with 1% triton X-100 following another two with lysis buffer alone and two more washes with preScission protease cleavage buffer (PS™ Protease, 270843, Amersham) and then resuspended in 70 µl of preScission protease cleavage buffer. An aliquot of 35 µl was diluted with 35 µl of the same buffer and treated with 4 µl of preScission protease for 48 h at 4°C and gently orbital shaking. An aliquot of 2 µl was stored at 4°C for later analysis, and the remaining volume was also stored to perform pull-down assays as described below. Supernatant from the cleavage reaction was collected and stored at −80°C by adding 25% glycerol and the process was repeated by adding 65 µl of fresh preScission protease cleavage buffer and collecting supernatant every 12 h for 2 more days. The 2 µl aliquots from the Gt-beads before and after cleavage were directly loaded in a 12% SDS–PAGE polyacrylamide minigel and analyzed by electrophoresis and colloidal Coomassie staining (Sigma-Aldrich).

#### Functional analysis of the r-tagged-hPCBP1(wt)

For the functional analysis of r-tagged-hPCBP(wt), 2-µg gDNA aliquots of obtained from human white blood cells, diluted into a volume of 100 µl with molecular biology grade water, and maintained on ice, were sonicated with a 150 V/T Ultrasonic Homogenizer (Biologics, Inc.) operated at 50% of its capacity. Following six 10-s sonication pulses with a 30-s ice-cooling interval between pulses, the aliquots were divided into two tubes (50 µl, 1 µg of gDNA each). 1-mL suspension of the r-10H-GST-PS-10H-EK-PCBP1(wt) attached to Gt-beads (from a total of 10 ml obtained from 1-l culture), was added to the first tube, and 1 ml of the light chain of the mouse enterokinase non-specific protein (similarly obtained) was added to the second tube. Following 2 h incubation at 4°C and gentle orbital shaking, the beads with the attached DNA fragments were washed 8 times with hepes buffer (Hepes 20 mM pH7.9; NaCL 400 mM; glycerol 25%; MgCl_2_ 1.5 mM; EDTA 1.5 mM; DTT 1 mM, PMSF 0.5 mM) for 5 min each time with gentle rotary shaking at 4°C. The final pellets were resuspended in 20 µl of molecular biology grade water. Following incubation at 96°C for 5 min, the supernatants were stored at −20°C. The pulled-down gDNA-specific fragments contained in the supernatants were studied by PCR with primers BRCA1-1 for *BRCA1* promoter detection; THRB-1 primers for *THRB* promoter detection; and OPRM1-1 primers for *OPRM1* promoter detection in those gDNA fragments. PCR were prepared in 20 µl volume that contained the manufacturer’s recommended buffer, dNTPs (0.2 mM), primers BRCA1-1 or THRB-1 or OPRM1-1 (0.2 µM), 1.4 µl of supernatant as template, and 1.25 U of Taq Pol. Amplification was performed with 1 denaturation cycle at 94°C (5 min); 35 amplification cycles composed of the following three stages, 94°C (30 s), 62°C for *BRCA1* or *OPRM1*, and 61°C for *THRB*, (30 s), 72°C (30 s); and 1 final cycle at 72°C (10 min), and the results were analyzed by agarose gel electrophoresis.

#### Analysis of promoter regulation by luminescent-reporter analysis

COS-7 cells were cultured following provider’s instructions. Cells were collected at confluence and distributed in six wells of a 24-well plate at a concentration of 1.5 × 10^5^ cells/well. After 24 h cells were washed with PBS and fresh medium was added. Five hours later, the cells from three wells were lipofectamine transfected with pGL4.10(luc2)-hSLC16A2pr(wt) (500 µg) and the remaining three wells with pGL4.10(luc2)-hSLC16A2pr(pr.-993_-985del), following manufacturer’s instructions. At 12 h post-transfection, the cells were washed with PBS and fresh medium was added. At 36 h post-transfection, the supernatants were removed, the cells washed with PBS once and immediately lysed by adding 100 µl of lysis buffer (25 mM Tris pH 7.8, 10% glicerol, 1% Tritón X-100, 2 mM DTT, 1 mM EDTA). Following centrifugation at 15.000 × *g* for 2 min, 80 µl of the supernatants were transferred to a 96 well-plate and 50 µl of luciferin were added to measure luminescence emission with a Fluostar Optima BMG LABTECH apparatus.

## Results

### CiPCR mechanisms: hypothesis

**Figure 2.**
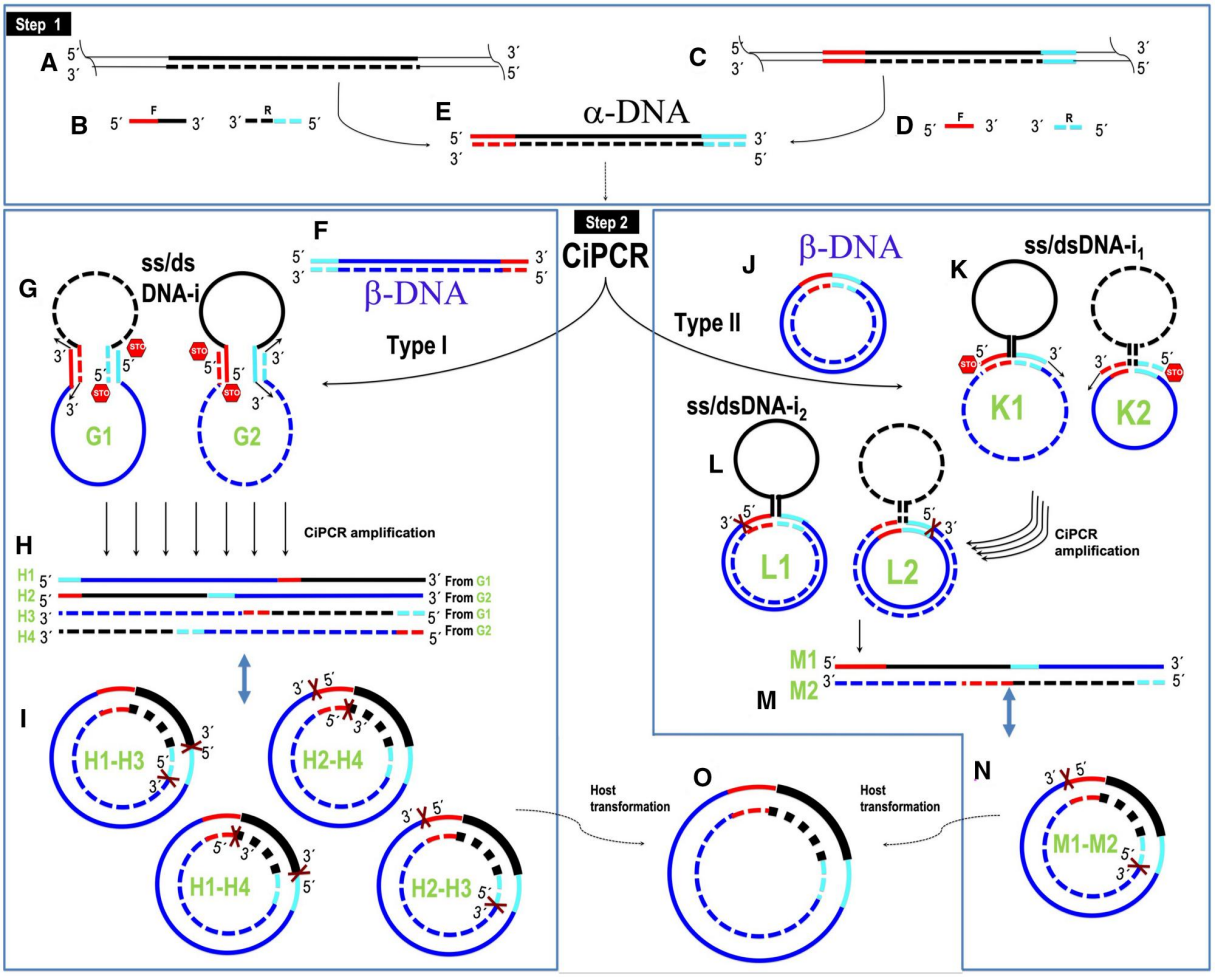
CiPCR hypothesis. (A)–(E) Generation of α-DNA (E) from dsDNA (A) with no homology to β-DNA with chimeric primers (B), or from dsDNA (C) with homology to β-DNA with normal primers (D). (F–I) Type I CiPCR. (F) β-DNA. (G) Ss/dsDNA-i stage. (H) Type I CiPCR ssDNA products. (I) Type I CiPCR circular dsDNA products containing staggered nicks. (J–N) Type II CiPCR. (J) β-DNA. (K) Ss/dsDNA-i_1_ stage. (L) Ss/dsDNA-i_2_ stage. (M) Type II CiPCR ssDNA products. (N) Type II CiPCR circular dsDNA product containing staggered nicks. (O) Plasmid DNA recovered after transformation of *E. coli* host. Regions of homology are denoted in red and cyan. DNA strand in the 5′–3′ direction, solid line, DNA strand in the 3′–5′ direction, broken line. The position of 5′- and 3′-ends is indicated.

We hypothesized that PCR could be used to integrate two dsDNA fragments (Fig. 1E, steps 1, 2, and 3), which we will call α- and β-DNA, into a circular dsDNA molecule. In principle, any linear ds α-DNA (Fig. 1E, step 1) with two different terminal sequences at either end that are homologous to two separate regions present in the β-DNA molecule (Fig. 1E2 and 3), could be used in this scheme. Annealing of α-DNA and β-DNA would result in a circular, hybrid ss/dsDNA intermediate (Fig. 1E, steps 4 and 5) that would be held together by ds regions of complementarity whose size would depend on the extent of homology. DNA synthesis at the available 3′-ends would result in a circular, dsDNA molecule with two staggered nicks. We termed this type of PCR circular PCR or CiPCR (Fig. 1E) and its basic mechanism is presented in detail in Fig. 2. In Fig. 2A–D, we show two possible sources for α-DNA. α-DNA can be generated by regular PCR using a template without natural homology to β-DNA (Fig. 2A), hence requiring chimeric primers (see Supplementary Material for its design) carrying such homology in their 5′ terminal sequences (Fig. 2B). Should the template naturally possess the required homology (Fig. 2C), a PCR step with regular primers targeted to the regions of homology (Fig. 2D) can be used to generate α-DNA (Fig. 2E). Regarding β-DNA, we distinguish Type I (Fig. 2F) and Type II CiPCR (Fig. 2J), depending on whether two or four 3′-ends are available for templated elongation in the *ss/dsDNA-i* (Fig. 2G and K). Type I CiPCR is depicted in Fig. 2E–I. The *ss/dsDNA-i*s intermediates created after annealing α-DNA and β-DNA (Fig. 2G) are extended via the available four 3′-ends to produce, upon denaturation, the four linear strands shown in Fig. 2H. At the end of the procedure, the annealing of different types of these strands, will give rise to four types of circular dsDNA molecules with two staggered nicks (Fig. 2I), whose location is given by the position of all four 5′-ends participating in the CiPCR (Fig. 2G).

Type II CiPCR is depicted in Fig. 2E–N for a β-DNA composed of a molecule of circular dsDNA (Fig. 2J) as mentioned above. Other possibilities are available for β-DNA in Type II CiPCR, as will be discussed below. Following annealing, only the two available 3′-ends present in the *ss/dsDNA-i_1_* (arrows in Fig. 2K) can be extended, giving rise to *ss/dsDNA-i_2_* in which the ds region covers the entirety of β-DNA (Fig. 2L). Upon denaturation, these structures would release the two types of linear strands shown in Fig. 2M which, at the end of the procedure, would give rise to one circular dsDNA molecule with two staggered nicks shown in Fig. 2N. Note that if the region between the homology sequences in β-DNA is the same in Fig. 2F and J, H2 = M1 and H3 = M2.

Finally, in the case that the final products of both Type I and Type II CiPCR carry plasmid-defining sequences (basically, an origin of replication and a selective marker), they can be transformed into an appropriate host, resulting in a seamless circular DNA molecule, that is, a plasmid (Fig. 2O). The use of *dam^+^* strains of *E. coli* as sources for β-DNA in Type II CiPCR is advisable, so that a simple DpnI treatment can destroy this template DNA prior to transformation of the final host [47].

As indicated above, when homology is already present in the template, α-DNA, in addition to PCR, can be alternatively generated by restriction digest, or by any other method producing blunt ends. Regarding β-DNA, Type I CiPCR requires linear templates with terminal homology as α-DNA. These can be easily generated by iPCR [23], but restriction cut is also valid. Note that for β-DNA, in Type I CiPCR, chimeric primers can alternatively be used to generate homology to α-DNA. In Type II CiPCR, β-DNA can be any linear or circular DNA with two internal sequences presenting homology to α-DNA. When circular DNA molecules serve as β-DNA, the two regions of homology may be continuous or separated and in the same or opposite orientation, relative to α-DNA. In the case of separated regions of homology with the same orientation, CiPCR gives rise to deletions and substitutions. Should the orientation be the opposite, an inversion of the intervening sequence is to be expected (not tested).

**Figure 3.**
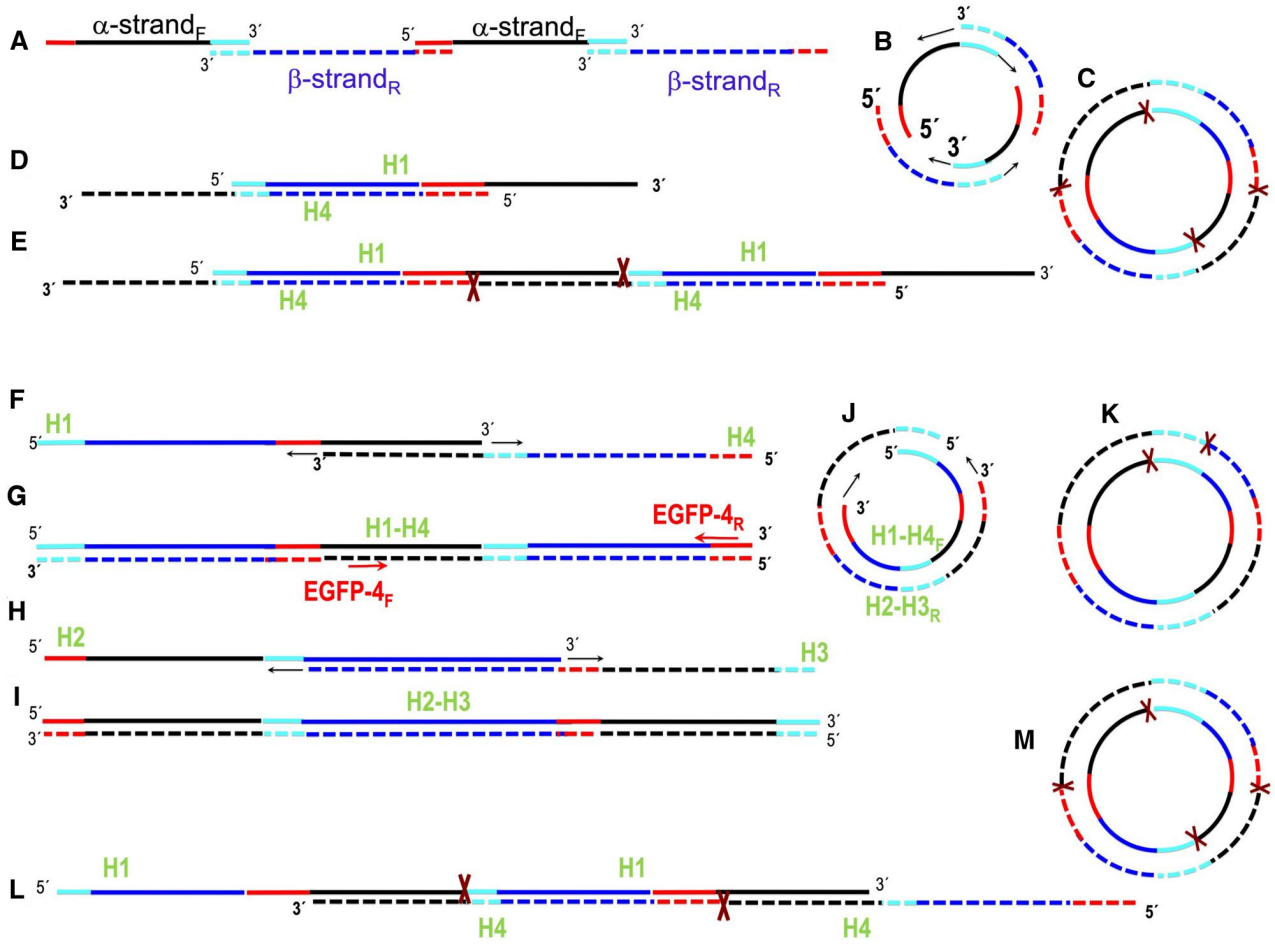
Potential formation of CiPCR byproducts. (A) Annealing of several α- or β-strands. (B) Ss/dsDNA-i stage following the annealing arrangements shown in (A). Arrows denote extension at the available 3′-ends. (C) Circularized CiPCR byproduct with two copies of α- or β-DNA and four nicks. (D) Annealing of H1 and H4 CiPCR strands via their 5′-ends. (E) Concatemer of H1 and H4 CiPCR product strands via their 5′-ends annealing. (F) Annealing of H1 and H4 CiPCR product strands via their 3′-ends. (G) Linear CiPCR byproduct generated by the extension of (F). (H) Annealing of H2 and H3 CiPCR product strands via their 3′-ends. (I) Linear CiPCR byproduct generated by the extension of (H). (J) Circularization of (G) and (I). (K**)** Circular CiPCR byproduct formed by the extension of (J). (L) Concatemer of H1 and H4 CiPCR product strands via their 3′-ends annealing. (M) Circular CiPCR byproduct formed from total annealing of concatemer (L). Strand names relative to the Type I CiPCR products shown in Figure 2H are provided for clarity. Black crosses, nicks; black arrows, extension at the 3′-end.

We also hypothesized that the large amount of homology displayed by CiPCR precursors and products could lead to an important but undetermined proportion of byproducts during the procedure. For example, during Type I CiPCR, the annealing of several α- or β-strands, followed by extension (Fig. 3A and B), could possibly lead to circularized, multiply nicked byproducts carrying more than one copies of α- and β-DNAs (Fig. 3C). The same type of circularized byproducts could be formed by the annealing of two normal Type I CiPCR product strands (e.g. H1 and H4, Fig. 2H), via their 5′-ends, as shown in Fig. 3D, followed by the annealing of two such complexes via their 3′-ends (Fig. 3E). Circularization of the complex shown in Fig, 3E, would also give rise to the circular dsDNA byproduct with four nicks, carrying two copies of α- and β-DNAs (Fig. 3C). Continuous strands longer than those of the target CiPCR products (Fig. 2H) could also be possibly formed. For example, the same Type I CiPCR strands shown in Fig. 3D (H1 and H4) might anneal via their 3′-ends, as shown in Fig. 3F, leading to a linear dsDNA with two copies of β-DNA (H1–H4 in Fig. 3G). Similarly, Type I CiPCR product strands H2 and H3 (Fig. 2H) (or M1 and M2 in Type II CiPCR) could anneal via their 3′-ends and become extended, leading a linear dsDNA with two copies of α-DNA (H2-H3 in Fig. 3I). Circularization between individual strands H1–H4 and H2–H3 from Fig. 3G and I, as shown in Fig. 3J, could yield a circular dsDNA byproduct with two nicks and two copies of α- and β-DNAs (Fig. 3K). More four-nicked **C**-analog products can also form from annealing between several alternative H strands, either through 5′- or 3′-annealing, as for example, 3′-annealing between H1 and H4 strands (Fig. 3L) to release **M** products (Fig. 3M). Next, we experimentally proved our CiPCR hypothesis.

### Construction of pcDNA3-SLC16A2(wt)-EGFP by insertion, using Type I and Type II CiPCR

**Figure 4.**
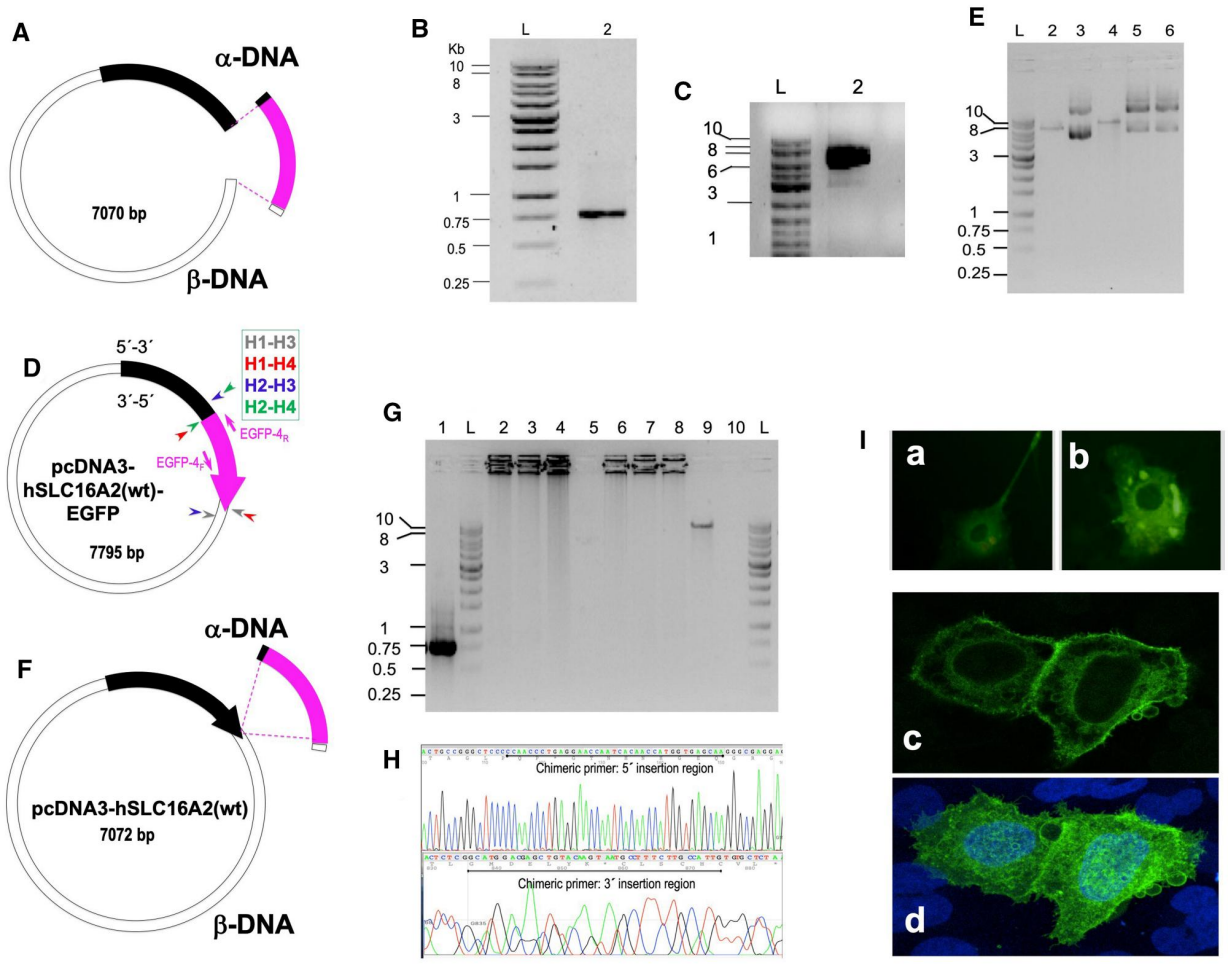
Construction of pcDNA3-hSLC16A2(wt)-EGFP by Type I and Type II CiPCR. (A) Schematic view of α- and β-DNA (Type I CiPCR1). *SLC16A2*, thick stripe on β-DNA; EGFP coding sequence, center stripe on α-DNA. Regions of homology are shown at both ends of α-DNA. (B) Electrophoresis analysis of α-DNA. Lane 2, PCR product (PCR1 in M&Ms). (C) Electrophoresis analysis of β-DNA. Gel. Lane 2, PCR product (iPCR1 in M&Ms). (D) pcDNA3-hSLC16A2(wt)-EGFP. Priming sites for the pair of primers EGFP-4 (F: forward, R: reverse, Table 1) are shown and their directionality is indicated by the small arrows next to the thick arrow. The location of the nicks is indicated with small arrowheads for the four different Type I CiPCR dsDNA products H–H (Figure 2I). The direction of transcription of the fusion gene is indicated by thick arrowhead. (E). Electrophoresis analysis of CiPCR cloned products. Lane 2, BamHI digested pcDNA3-hSLC16A2(wt) (7072 bp); lane 3, pcDNA3-SLC16A2, uncut control; lane 4, BamHI digested pcDNA3-hSLC16A2(wt)-EGFP (7795 bp); lanes 5 and 6, pcDNA3-hSLC16A2(wt)-EGFP, uncut control. (F) Schematic view of the α- and β-DNA used in Type II CiPCR2. The direction of transcription of the *hSLC16A2* gene is indicated by thick arrowhead. (G) Electrophoresis analysis of potential CiPCR byproducts. Lane 1, α-DNA PCR product (same as in gel B, PCR1 in M&Ms). Lane 2, Type II CiPCR products before DpnI treatment; lanes 3–4, Type II CiPCR products after DpnI treatment; lane 5, pcDNA3-hSLC16A2(wt) at the same concentration as in lanes 2–4; lanes 6–8, iPCR amplification of the products shown in lanes 2–4, respectively; lane 9, iPCR amplification of pcDNA3-hSLC16A2(wt)-EGFP obtained from Type II CiPCR after nick repair in *E. coli* (expected size 7789) lane 10, iPCR negative control. (H) Electropherograms showing the sequence of the junction between α- and β-DNA in pcDNA3-SLC16A2(wt)-EGFP (chimeric primer regions are indicated). (I) Expression of MCT8-EGFP observed under a fluorescence microscope in A-172 (a) and in NCI H-460 (b) cells, and in a confocal microscope in NCI H-460 cells (c (section) and d (whole field)). Molecular weights in kbp are indicated in (B), (C), (E), and (G).

To prove our hypothesis, we decided to create a C-terminal fusion of EGFP to the human gene *SLC16A2* known as solute carrier family 16 member 2, encoding the protein monocarboxylate transporter 8 (MCT8), a transmembrane thyroid hormone (TH) transporter that regulates the entrance of TH into cells [48]. The ORF of *hSLC16A2* was carried in pcDNA3-hSLC16A2(wt). We first attempted Type I CiPCR. In this case, α-DNA, consisted of a 763-bp dsDNA fragment containing the ORF of EGFP (small, pink fragment in Fig. 4A) that was amplified by regular PCR using pIRES2-EGFP as a template (PCR1 in Materials and Methods, M&Ms, Fig. 4B). As β-DNA, we used a 7070-bp dsDNA fragment resulting from the iPCR amplification of a region of pcDNA3-SLC16A2(wt) (iPCR1 in M&Ms, Fig. 4C) carrying the origin of replication, antibiotic resistance marker, and the coding sequence of *hSLC16A2* (large fragment in Fig. 4A). We ensured that both fragments carried terminal homology regions by the use of chimeric primers described in PCR1, resulting in an α-DNA carrying sequences of 20 and 18 nts at its ends that were homologous to the terminal regions of β-DNA. Transformation of *E. coli* TG1 cells with the CiPCR1 products displayed an efficiency of 4 × 10^2^ colonies/µg of β-DNA. Transformants were found to harbor the target pcDNA3-hSLC16A2(wt)-EGFP by colony-PCR, restriction analysis, and sequencing (Fig. 4E and Supplementary Fig. S1).

We also constructed pcDNA3-hSLC16A2(wt)-EGFP by Type II CiPCR (CiPCR2, Fig. 4F). In this case, α-DNA was the same as in Fig. 4B (see Fig. 4G, lane 1), whereas β-DNA was composed of pcDNA3-hSLC16A2(wt) in its circular form Fig. 4F. Following the CiPCR2, we obtained a transformation efficiency of up to 2 × 10^4^ colonies/µg. Transformants were found to harbor the pcDNA3-hSLC16A2(wt)-EGFP target by colony-PCR. Sequencing analysis of the whole α-DNA and flanking regions was used to verify that Type I and II CiPCR gave rise to the exact same circular DNA products, as predicted (Fig. 4H and Supplementary Fig. S1).

The large amount of homology presented by the expected CiPCR products shown in Fig. 2H and M, and of these products with α-DNA and β-DNA, is expected to provide ample opportunities for the generation of undesired CiPCR byproducts. As shown in Fig. 4G (lanes 2–4) Type II CiPCR products (CiPCR2) form a smear on an electrophoresis gel, consistent with the idea that dsDNA products of continuous sizes are generated in the reaction (Fig. 3). We were particularly worried about the presence of byproducts with continuous unnicked strands longer than that of the expected CiPCR products (Fig. 2H and M). We hypothesized that, in case that linear products were formed with sizes longer that those of the expected products (Fig. 3G and I), or the potentially derived circular byproducts (Fig. 3K), they would carry more than one copy of α- or β-DNA, or both. IPCR amplification performing with a pair of primers as those shown in Fig. 4D (iPCR2 in M&Ms), would only be productive if CiPCR products with, at least, two copies of α-DNA present on a continuous unnicked strand were generated (i.e. Fig. 3G, I, and K). Lanes 6–8 in Fig. 4G show our failure to identify such products, whereas we could easily amplify a control composed of intact pcDNA3-hSLC16A2(wt)-EGFP (lane 9). This indicates that only nicked concatemers are responsible for the observed smear in lanes 2–4, which in any case the products trapped in these nicked concatemers are recuperated during the respective melting stages of each CiPCR cycle.

We analyzed the expression of the chimeric protein encoded by the h*SLC16A2*(wt)-EGFP fusion present in pcDNA3-hSLC16A2(wt)-EGFP. As shown in panels a–d of Fig. 4I, expression of MCT8-EGFP can be easily detected following transfection of human cell lines with pcDNA3-hSLC16A(wt)-EGFP. The fusion protein is located preferentially at the membrane surface and perinuclear areas, as expected for a transmembrane protein (panel c in Fig. 4I).

### Construction of pSUPER.neo-Si-hSLC16A by insertion, using Type II CiPCR

**Figure 5.**
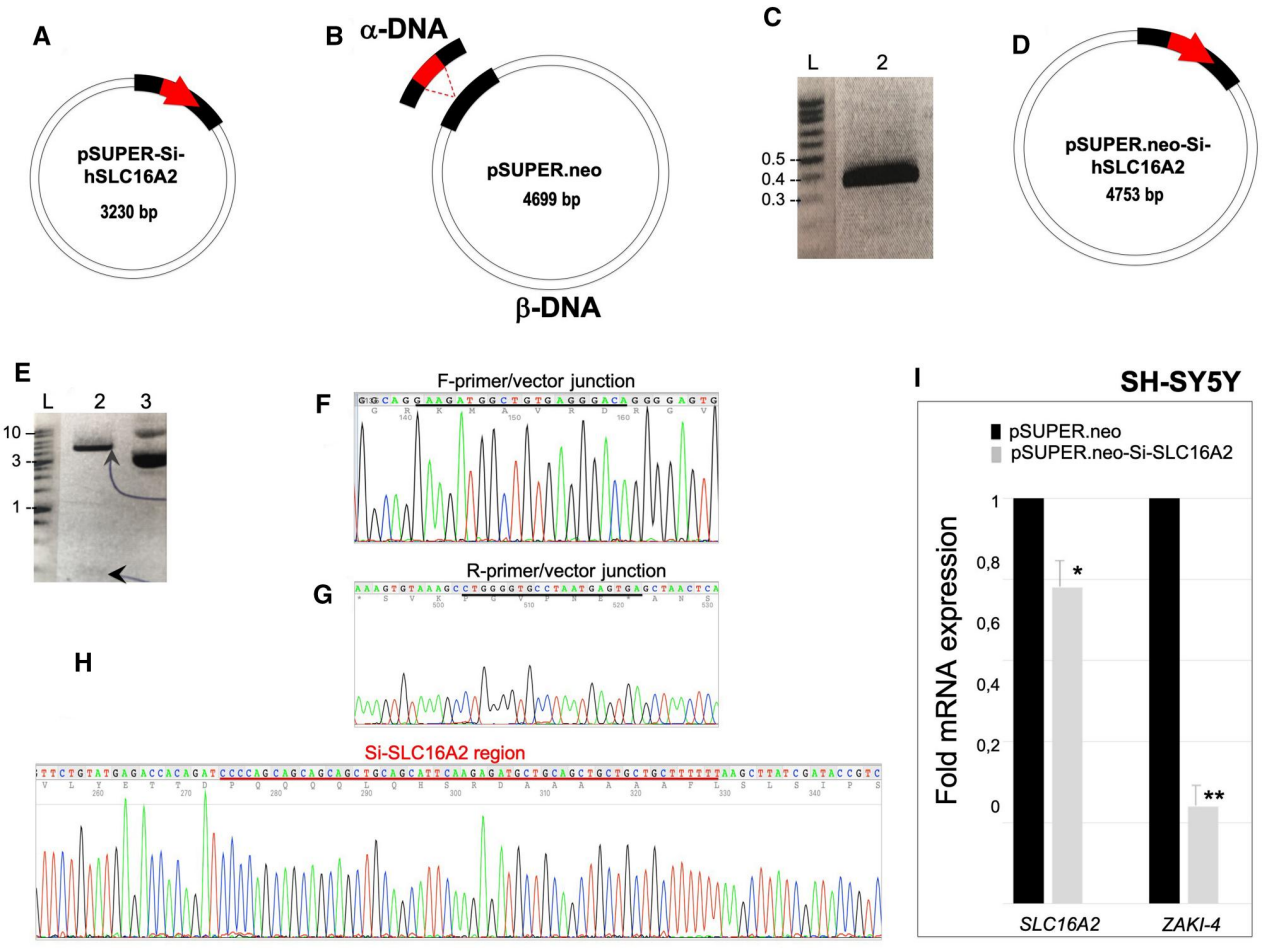
Construction of pSUPER.neo-Si-hSLC16A2. (A) pSUPER-Si-hSLC16A2. Black thick stripes, internal homology sites in the β-DNA. Thick arrow, silencer sequence. The direction of transcription is indicated by thick arrowhead. (B) Schematic view of the α- and β-DNAs used in this procedure (CiPCR3 in M&Ms). Regions of homology are shown at both ends of α-DNA. (C) Electrophoresis analysis of the 382-bp α-DNA (PCR2 in M&Ms). Lane 2, α-DNA. (D) Predicted pSUPER.neo-Si-hSLC16A2 construct. (E). Restriction analysis of pSUPER.neo-Si-hSLC16A2. Lane 2, EcoRI–XhoI digest with arrows indicating digest products; lane 3, uncut control. (F)–(G). Electropherograms showing the sequence of the forward (F) and reverse (G) junctions between α- and β-DNA in pSUPER.neo-Si-hSLC16A2. The underlined sequence represents the α-DNA ends. (H) Electropherogram showing the silencer sequence (underlined) (I). Silencing efficiency of pSUPER.neo-Si-hSLC16A2. X axis, gene; y axis, fold change of mRNA expression in a.u. Dark boxes, control SH-SY5Y cells carrying pSUPER.neo; light boxes, SH-SY5Y cells carrying pSUPER.neo-Si-hSLC16A2. Error bars and levels of significance are shown (NS, *, **, ***, non-significant, significant for a *P* < 0.05, 0.01, or 0.001, respectively). Molecular weights in kbp are indicated in (C) and (E).

We used CiPCR to generate a construct capable of silencing *SLC16A2* expression in human cell lines (Fig. 5). To do this, we used the small interfering RNA (si-RNA) expression vector pSUPER.neo (OligoEngine). In this case, a 382-bp α-DNA fragment (Fig. 5C, lane 2) was obtained via standard PCR (PCR2 in M&Ms) using a previously constructed plasmid, pSUPER-Si-hSLC16A2 (Fig. 5A) that carried the 60-nt silencer sequence (Si-*SLC16A2*, Table 1). Type II CiPCR (CiPCR3 in M&Ms) was used to transfer the 60-bp silencer sequence to the geneticin expressing vector pSUPER.neo (β-DNA, Fig. 5B) using the existing homology between the two vectors, namely 130 nts of 5′- and 192 nts of 3′-terminal homology present in α-DNA. The integrity of pSUPER.neo-Si-hSLC16A2 (Fig. 5D) was analyzed by restriction and sequencing analysis (Fig. 5E–H).

We tested the functionality of the pSUPER.neo-Si-hSLC16A2 construct following stable transfection into SH-SY5Y cells. After 8 days of culture under selection with geneticin, the expression levels of the endogenous *hSLC16A2* mRNA in cells with pSUPER.neo-Si-hSLC16A2, or in control cells carrying pSUPER.neo, were analyzed via qPCR. The results show a small, but statistically significant 22% decrease of transcription (*P* < 0.05) (Fig. 5I). We also measured the transcription of the gene *ZAKI-4*, known to be strongly affected by TH levels [49]. A dramatic statistically significant 86% decrease of *ZAKI-4* mRNA was detected (*P* < 0.01), indicating that even modest reductions in the expression of *SLC16A2* induce large expression differences in downstream genes (Fig. 5I).

### Construction of p2GUS-10H-GST-PS-10H-EK-PCBP1(wt) by replacement, using Type II CiPCR

**Figure 6.**
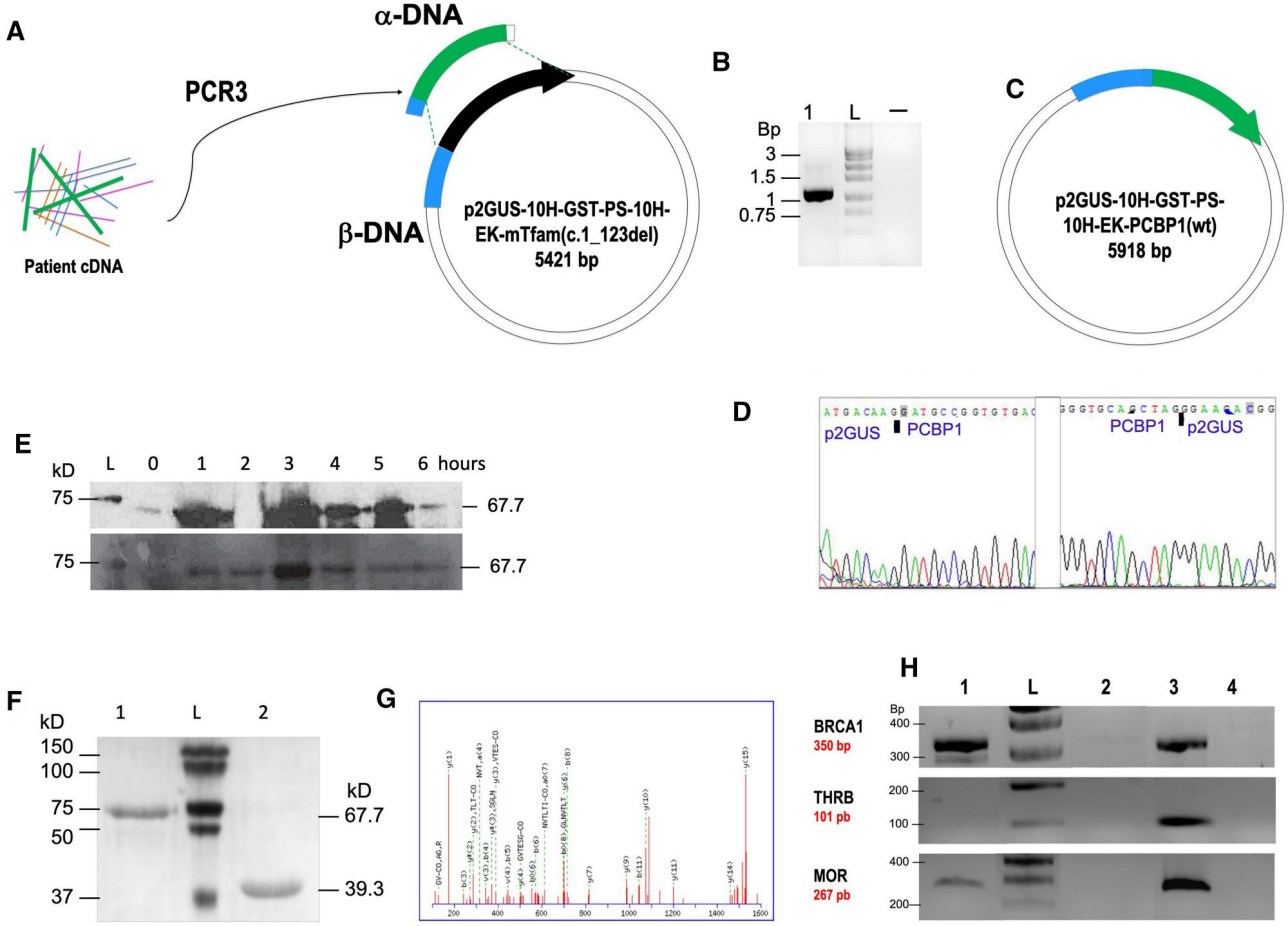
Construction of p2GUS-10H-GST-PS-10H-EK-PCBP1(wt). (A) Cloning scheme. The thick stripe on the β-DNA preceeding the thick arrow contains the 10H tag, followed by a GST tag, a PS target sequence, a second 10H tag and an enterokinase target sequence. The *10H-GST-PS-10H-EK-mTfam(c.1_123del)* coding sequence is shown by the thick arrow. The direction of transcription is indicated by the thick arrowhead. (B) Electrophoresis analysis of α-DNA. Lane 1, PCR product (PCR3 in M&Ms), lane -, negative PCR control. (C) Final predicted construct p2GUS-10H-GST-PS-10H-EK-hPCBP1(wt). (D) Electropherograms showing the sequence of the junction between α- and β-DNA in p2GUS-10H-GST-PS-10H-EK-PCBP1(wt). (E). Western blot analysis of r-10H-GST-PS-10H-EK-hPCBP1(wt) expression following arabinose induction. Upper panel using a polyhistidine antibody for detection. Lower panel using a GST antibody for detection; lanes 1–6, time in hours. (F) SDS–PAGE analysis of r-tagged-hPCBP1(wt). Lane 1 fully tagged-PCBP1(wt); lane 2, 10H-EK-hPCBP1(wt). (G) Mass spectrum histogram of the r-tagged-hPCBP1(wt). (H) Electrophoresis analysis of BRCA1 (upper panel), THRβ (center panel) and OPRM1 (lower panel) promoter PCR- amplified fragments. Lane 1, gDNA/r-tagged-PCBP1(wt) pull-down DNA fragments; lane 2 gDNA/light chain of a mouse enterokinase protein negative control for non-specific pull-down; lane 3 PCR-amplified fragments from the gDNA used for the pull-down assays; lane 4, PCR negative control. Protein molecular weights in kD, and dsDNA fragments in kbp, are indicated.

Fig. 6A shows the schematic view of this procedure. Total cDNA was generated by retro-transcription following mRNA extraction from a patients’ white blood cells. PCR amplification of this cDNA with the pair of chimeric primers PCBP1-1 (Table 1) (PCR3), yielded a 1104-bp DNA fragment containing the *hPCBP1(wt)* ORF (Fig. 6B) to be used as α-DNA in this procedure (Fig. 6A). The pair of chimeric primers PCBP1-1 carried homology to non-contiguous sites of p2GUS-10H-GST-PS-10H-EK-mTfam(c.1_123del), to be used as β-DNA (Fig. 6A). Type II CiPCR4 on this plasmid with the aforementioned α-DNA would result in the replacement of a 567-bp fragment, carrying the partial ORF of the *mTfam(c.1_123del)* gene, with the *hPCBP1(wt)* ORF, yielding p2GUS-10H-GST-PS-10H-EK-PCBP1(wt) (Fig. 6C). The sequencing analysis corroborated the insertion of the *hPCBP1* α-DNA in the final construct p2GUS-10H-GST-PS-10H-EK-PCBP1(wt) (Fig. 6D).

The expression of p2GUS-10H-GST-PS-10H-EK-PCBP1(wt) is under control of the P_BAD_ promoter (own vector, manuscript in preparation), which can be induced by arabinose to initiate the synthesis of the recombinant protein [42]. Since p2GUS-10H-GST-PS-10H-EK-PCBP1(wt) carried a 10-histidine (10H) tag, followed by a glutathione S-transferase (GST) tag, a target sequence for the preScission (PS) protease, a second 10H tag and a target sequence for the enterokinase (EK) protease (blue stripe in Fig. 6A and C), the expected molecular weight of the recombinant protein r-10H-GST-PS-10H-EK-PCBP1 is 67.7 kD. In agreement with this, Fig. 6E shows a time-course experiment analyzed by Western blot with an polyhistidine antibody (upper panel) (Table 2) and reblotted with an GST antibody (lower panel) (Table 2) showing the detection of bands with the expected molecular weight for r-10H-GST-PS-10H-EK-PCBP1 with the maximum expression at 3 h upon arabinose induction. Figure 6F further corroborate these findings showing the detection of a band of the same size after being purified by Nickel affinity (Table 2) followed by Gt affinity (Table 2) chromatography (lane 1 on the SDS–PAGE gel analysis). Lane 2 shows the same protein after being cut with preScission protease were a band with a smaller molecular weight of 39.3 kD is observed, which agrees to the expected size for r-10H-EK-PCBP1. Mass spectrometry analysis of the purified bands (Fig. 6G), corroborated the integrity of both proteins, namely the fully tagged and partially tagged versions of r-PCBP1. Furthermore, hPCBP1 was reported to be involved in the transcription of *hBRCA1* [50] and human and mouse *OPRM1 (MOR)* [51] genes. Based on that information, pull-down assays with gDNA from human white blood cells using the r-fully-tagged-PCBP1 obtained as described above, still attached to Gt beads, were performed. The pulled-down gDNA specific fragments contained in the supernatants, analyzed by PCR generate for BRCA1-PCR, 327-bp dsDNA fragment; for THRB-PCR, 101 bp fragment; and for OPRM1-PCR, 268 bp fragment. In Fig. 6H, lane 1 (top panel) shows a positive amplification with specific primers BCRA1-1 (Table 1) to detect *hBCRA1*promoter–PCBP1 interactions when r-hPCBP1 is used for the pull-down of gDNA contrasting to the negative result observed on lane 2 using the non-specific protein (similarly obtained from construct p2GUS-10H-GST-PS-mTmprss(c.1_2582del)), as a negative control. Similar results are observed from the PCR to detect *hOPRM1*promoter–PCBP1 interactions (Fig. 6H, bottom panel), with OPRM1-1 primers (Table 1) by PCR showed on lane 1. As expected, there is no detection of the TH receptor beta (THRB gene) promoter–PCBP1 interactions (Fig. 6H, center panel), as this gene-promoter was not reported to be regulated by PCBP1. These results demonstrate that r-10H-GST-PS-10H-EK-hPCBP1(wt) cloned by CiPCR and obtained by the described expression protocol is fully functional.

### Construction of pGL4.10(luc2)-hSLC16A2pr(pr.-993_-985del) by deletion, using Type II CiPCR

**Figure 7.**
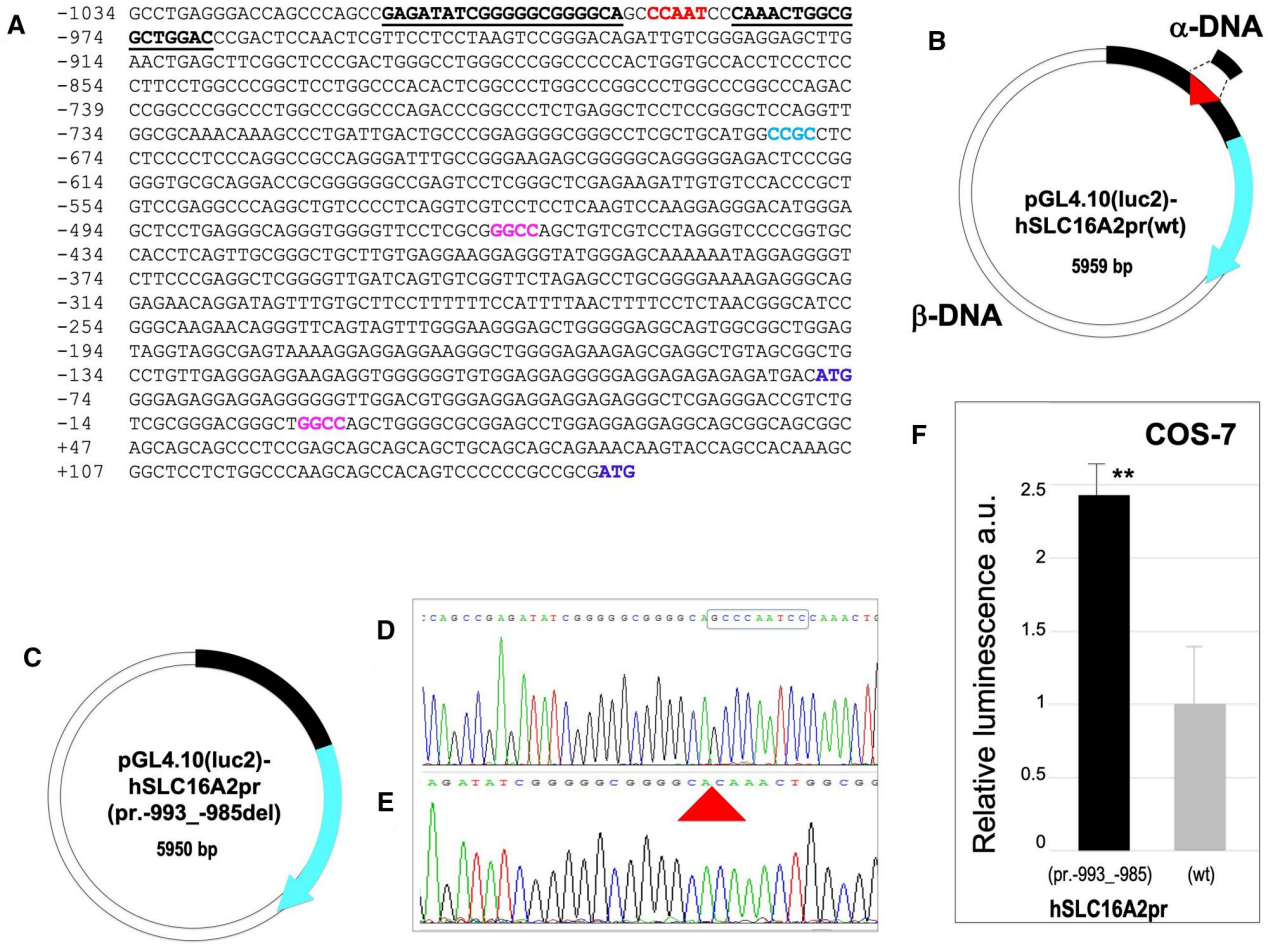
Construction of pGL4.10(luc2)-hSLC16A2pr(pr.-993_-985del) by deletion. (A) Partial sequence of the *SLC16A2* gene promoter (h*SLC16A2*pr) showing the +1TSS (GGCC, bold font at position +1), possible TSS for a shorter transcript (an alternative larger transcript is also shown at position −461 (GGCC, bold font) (TRANSFAC (https://genexplain.com/transfac/) predicted TSS (CCGC, bold font at position -681)). CCAAT box (bold font starting at position -991), the two alternative TIS (ATG, bold font at positions -77 and +146 respectively), and the region of homology contained in the chimeric primers used in this procedure (bold font underlined). (B) Type II CiPCR scheme. The thick stripe, h*SLC16A2*pr sequence. The thick triangle, CCAAT box location.Thick arrow following the thick-stripe, luciferase. The direction of transcription is indicated by the arrowhead. **C**. Expected CiPCR (CiPCR5) product, following transformation into *E. coli* host. (D)–(E) Electropherogram of the original (D) and deletion plasmids (E). The target sequence is indicated with a box in (D). The thick triangle in E indicates the site of deletion. (F) Luminescence assays. x axis, construct; y axis, relative luminescence in arbitrary units a.u.). Error bars and levels of significance are shown (NS, *, **, ***, non-significant, significant for a *P* <0 .05, 0.01, or, 0.001, respectively).

The **CCAAT** box of the h*SLC16A2* promoter is located between positions −991 to −987, upstream of the most common transcription start site (TSS) indicated at position +1 (Fig. 7A) and before the second translation initiation site (TIS; Fig. 7A). The h*SLC16A2* promoter in pGL4.10(luc2)-hSLC16A2pr(wt), indicated by the thick black stripe with a red triangle pointing to the CCAAT box, is followed by the luciferase CDS fused to it (Fig. 7B, cyan arrow). To delete the **CCAAT,** indicated as a red triangle, we designed the pair of chimeric primers CCAAT-1 (underlined in Fig. 7A), whose sequence includes the region surrounding the **CCAAT** box, but lacks the nine nucleotides GC**CCAAT**CC, which include this element. They were used directly in a Type II CiPCR (CiPCR5 in M&Ms) to yield the construct pGL4.10(luc2)-hSLC16A2pr(pr.-991_-987del) (Fig. 7C), whose integrity was corroborated by sequencing (Fig. 7D).

The two constructs were transfected into COS-7 cells to perform luminescence analysis. The **CCAAT**-deleted construct displayed close to a 2.5-fold stronger statistically significant (*P*-value < 0.01) induction relative to the construct with the intact promoter (Fig. 7F).

### Construction of single-nucleotide mutations in pcDNA3-hSLC16A2(wt) by SDM, using Type II CiPCR

**Figure 8.**
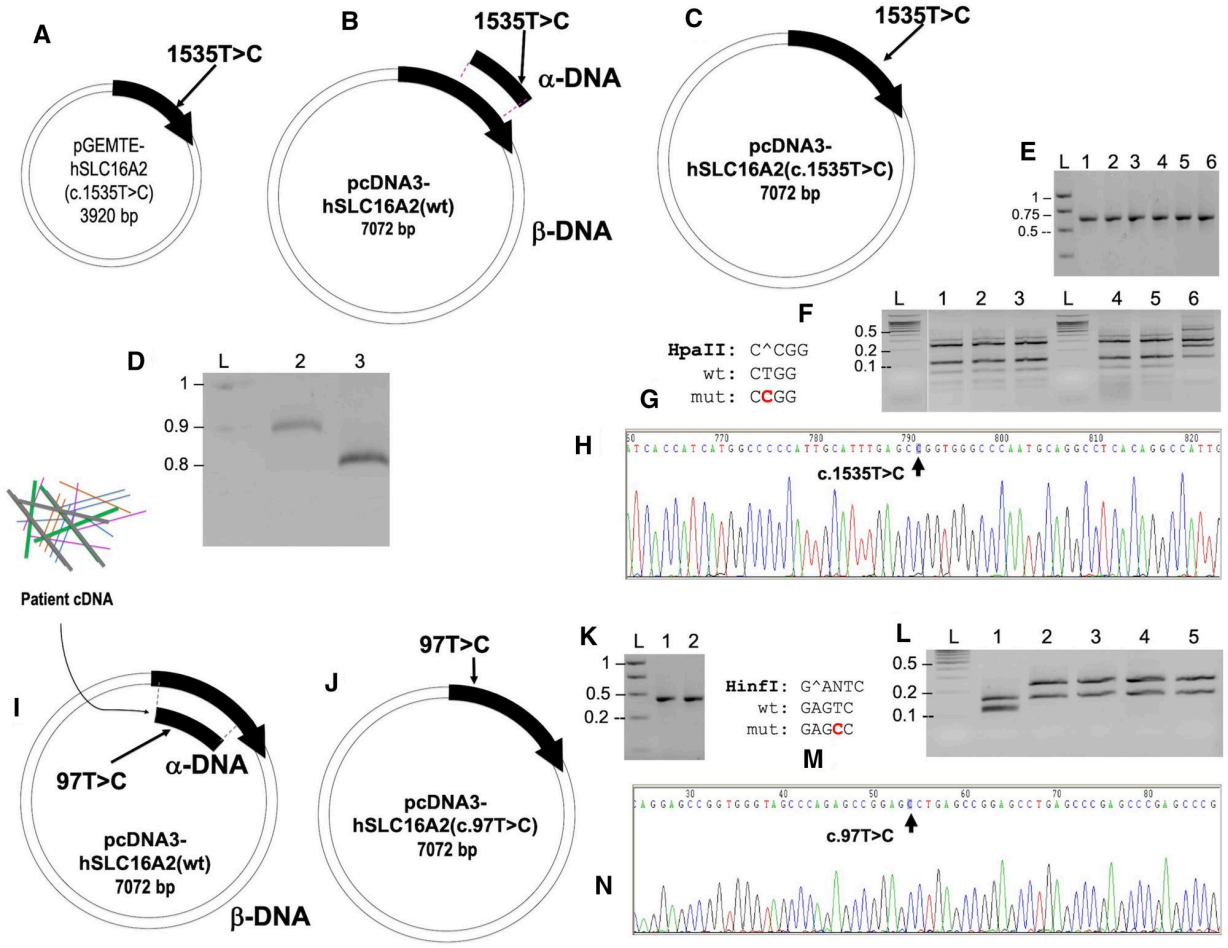
Construction of single nucleotide mutations in pcDNA3-hSLC16A2(wt). (A) Construct pGEMTE-partial-hSLC16A2 (c. 1535 T > C). Partial-h*SLC16A2* (c. 1535 T > C) is shown as thick, black arrow indicating the direction of transcription. The approximate location of the c.1535T>C variant is indicated. (B and C). Type II CiPCR scheme (B) for the construction of pcDNA3-hSLC16A2(c.1535T>C (C). α- and β-DNA are indicated. (D) Electrophoresis analysis of 894-bp α-DNA Lane 2. (E) Colony-PCR analysis of transformants, 689-bp PCR fragment, Lanes 1–5, pcDNA3-hSLC16A2(c.1535T>C) from 5 independent transformants; lane 6, control pcDNA3-hSLC16A2(wt). (F) Restriction analysis with Hpa II corresponding to the six 689-bp fragments shown in (E). (G) Hpa II target sequence as well as the wt and mutant alleles are shown for clarity. (H) Electropherogram of construct pcDNA3-hSLC16A2(c.1535T>C) showing the region around the c.1535T>C mutation. (I and J) Type II CiPCR scheme (I) for the construction of pcDNA3-hSLC16A2(c.97T>C) (J). The 812-bp Lane 3, in D corresponds to the α-DNA, and α- and β-DNA are indicated in (I). (K) A 400-bp fragment for the colony-PCR of transformants. Lane 1, control PCR-fragment from pcDNA3-hSLC16A2(wt); lane 2, PCR-fragment from pcDNA3-hSLC16A2(c.97T>C) from transformant 2 (transformants 3–5 showed identical PCR-fragments). (L) Restriction analysis of the 4 PCR-fragments from independent transformants 2-5 with Hinf (I). Lane 1, control pcDNA3-hSLC16A2(wt); lanes 2–5, pcDNA3-hSLC16A2(c.97T>C). (M) HinfI target sequence as well as the wt and mutant alleles are shown for clarity. (N) Electropherogram of construct pcDNA3-SLC16A2(c.97T>C) showing the region around the c.97T>C mutation.

An 894-bp mutagenic insert carrying the pathogenic h*SLC16A2*(c.1535T>C) transition, found in the allelic variant rs104894931 (OMIM 300095.0001) [52], was generated with the pair of primers SDM-L512P-1 (Table 1). This variant causes a L512P amino acid replacement in the sequence of the most commonly expressed MCT8 protein variant, as a larger protein product from an upstream TIS is also expressed in same tissues [52]. Construct pGEMTE-hSLC16A2(c.1535T>C), carries a copy of the *hSLC16A2* gene with the 1535 T > C variant (Fig. 8). PCR4 was used to generate an 894-bp α-DNA carrying the variant. Type II CiPCR with pcDNA3-hSLC16A2(wt), as β-DNA yielded pcDNA3-hSLC16A2(c.1535T>C) (Fig. 8B–D). Since the 1535 T > C variant creates a HpaII restriction site, we checked few colonies for the presence of the variant by colony-PCR with primers SDM-L512P-2, which yield a 689-bp fragment, followed by restriction analysis. These results are shown in Fig. 8E–G, indicating that all clones carry the mutation, Sequencing analysis confirmed these results (Fig. 8H).

A second *hSLC16A2* variant, namely the h*SLC16A2*(c.97T>C) transition at position 97 (allelic variant rs6647476) **(**GWAS **rs6647476**) was also cloned. This non-pathogenic SNP variant causes the S33P aminoacid replacement in the sequence of the shorter MCT8 protein product (or S107P if referred to the large MCT8 protein product from the first TIS) [53]. The mutation was introduced in pcDNA3-hSLC16A2(wt) by Type II CiPCR. In this case, an 812-bp α-DNA was directly generated by PCR with primers S33P-2 from patients’ mRNA carrying this variant (PCR5 see M&Ms) (Fig. 8D, lane 3). The construct pcDNA3-hSLC16A2(wt) was used as β-DNA in this procedure (Fig. 8I) to yield pcDNA3-hSLC16A2(c.97T>C (Fig. 8J). Since the 97T>C variant destroys the Hinf I restriction site present in the wild type (wt) sequence, we checked few transformants for the presence of the variant by colony-PCR with primers SDM-S33P-2, which yield a 400-bp fragment, followed by restriction analysis, as shown in Fig. 8K–M, indicating that all transformants harbor the mutation. Sequencing analysis confirmed these results (Fig. 8N).

### Enlargement of terminal homology regions of α-DNA

**Figure 9.**
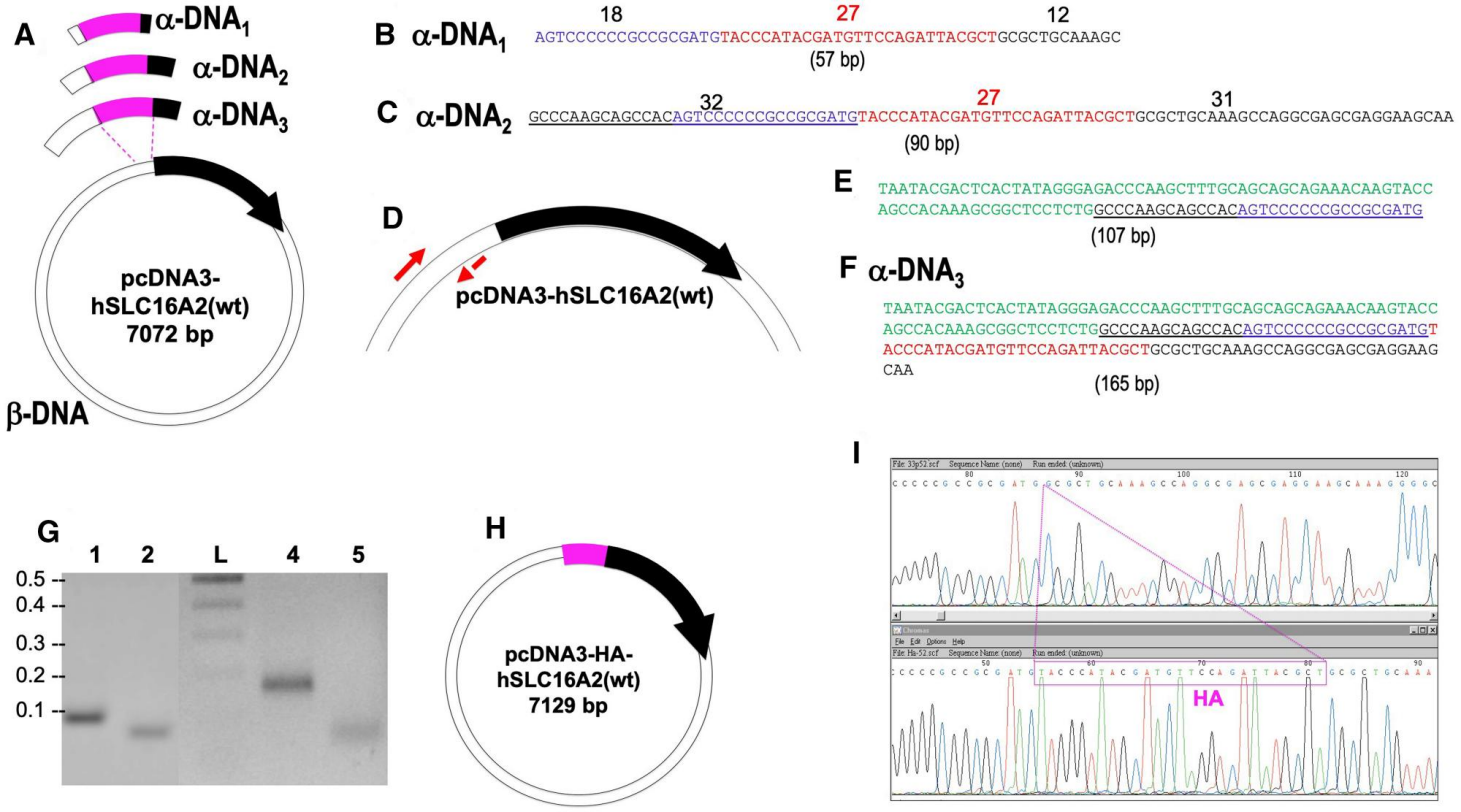
Enlargement of terminal homology regions. (A) Type II CiPCR scheme with α-DNAs_1–3_ and β-DNA (pcDNA3-hSLC16A2(wt)). Thick arrow in β-DNA, h*SLC16A2* CDS. The thick stripe in the center of the α-DNAs_1–3_, HA. The direction of transcription is indicated by arrowhead. (B) α-DNA_1_, forward strand. (C) α-DNA_2_, forward strand. (D) PCR8 with the pair of primers HA-4 (small arrows) and template pcDNA3-hSLC16A2(wt). (E) Sequence of the forward strand of 107-bp DNA fragment amplified by PCR8. (F) α-DNA_3_, forward strand. Homology between α-DNA_2_, 107-bp fragment and α-DNA_3_ (for OE-PCR1 in M&Ms) is underlined. All sequences are shown in the 5′- to 3′-direction. (G) Electrophoresis analysis of the intermediates used in this procedure. Lane 1, α-DNA_2_ (90-bp) (From PCR7 in M&Ms); lane 2, PCR7 negative control; lane 4, α-DNA_3_ (165-bp) (from OE-PCR1 in M&Ms); lane 5, OE-PCR1 negative control. Molecular weights in kbp are indicated. (H) pcDNA3-HA-hSLC16A2(wt). (I) Electropherograms of pcDNA3-hSLC16A2(wt) (top panel) and pcDNA3-HA-hSLC16A2(wt) (bottom panel). The position of the HA tag is indicated with a light box.

We have found that for the successful completion of some CiPCR procedures, a large extent of homology between α-DNA and β-DNA must be used. When large homology regions are to be created synthetically, rather than by the amplification of natural DNA sequences, the creation of large synthetic oligonucleotides can be performed, that is, as shown in Fig. 9. In this example, we attempted to add the hemagglutinin (HA) tag to the N-terminus of *hSLC16A2(wt)* by Type II CiPCR, using the construct pcDNA3-hSLC16A2(wt) as β-DNA. As α-DNA_1_, we designed the pair of primers HA-1 that carried the 27-nt HA sequence between two regions of terminal homology to the above β-DNA, namely an 18- and a 12-nt region of homology (Fig. 9A and B). Since Type II CiPCR with α-DNA_1_ failed to produce the expected construct, we extended the two regions of terminal homology to 32 and 31 nts (PCR7 in M&Ms) with primers HA-2, to obtain a 90-bp PCR product that could be used as α-DNA_2_ (Fig. 9A, C, and G, lane 1). Again, failure of Type II CiPCR with α-DNA_2_ led us to perform a drastic, new round of enlargement, now carried out in two steps. First, a 107-bp dsDNA fragment was amplified by regular PCR (PCR8 in M&Ms) with the pair of primers HA-4 and pcDNA3-hSLC16A2(wt) as a template (Fig. 9D–E). The homology shared by the 107-bp fragments from PCR8 and α-DNA_2_ was used to obtain α-DNA_3_ (165-nt) by OE-PCR (OE-PCR1 in M&Ms) (Fig. 9F and G, lane 4) with primers HA-3, which was used in a new Type II CiPCR (CiPCR9 in M&Ms) (Fig. 9A) that gave rise to the desired product pcDNA3-HA-hSLC16A2(wt) (Fig. 9H). The integrity of pcDNA3-HA-hSLC16A2(wt) was verified by sequencing (Fig. 9I).

### Simultaneous insertion and deletion by Type II CiPCR

The following section provides an example of the use of Type II CiPCR for simultaneous insertion and deletion and, at the same time, it offers solutions for the design of long, fully synthetic α-DNAs.

**Figure 10.**
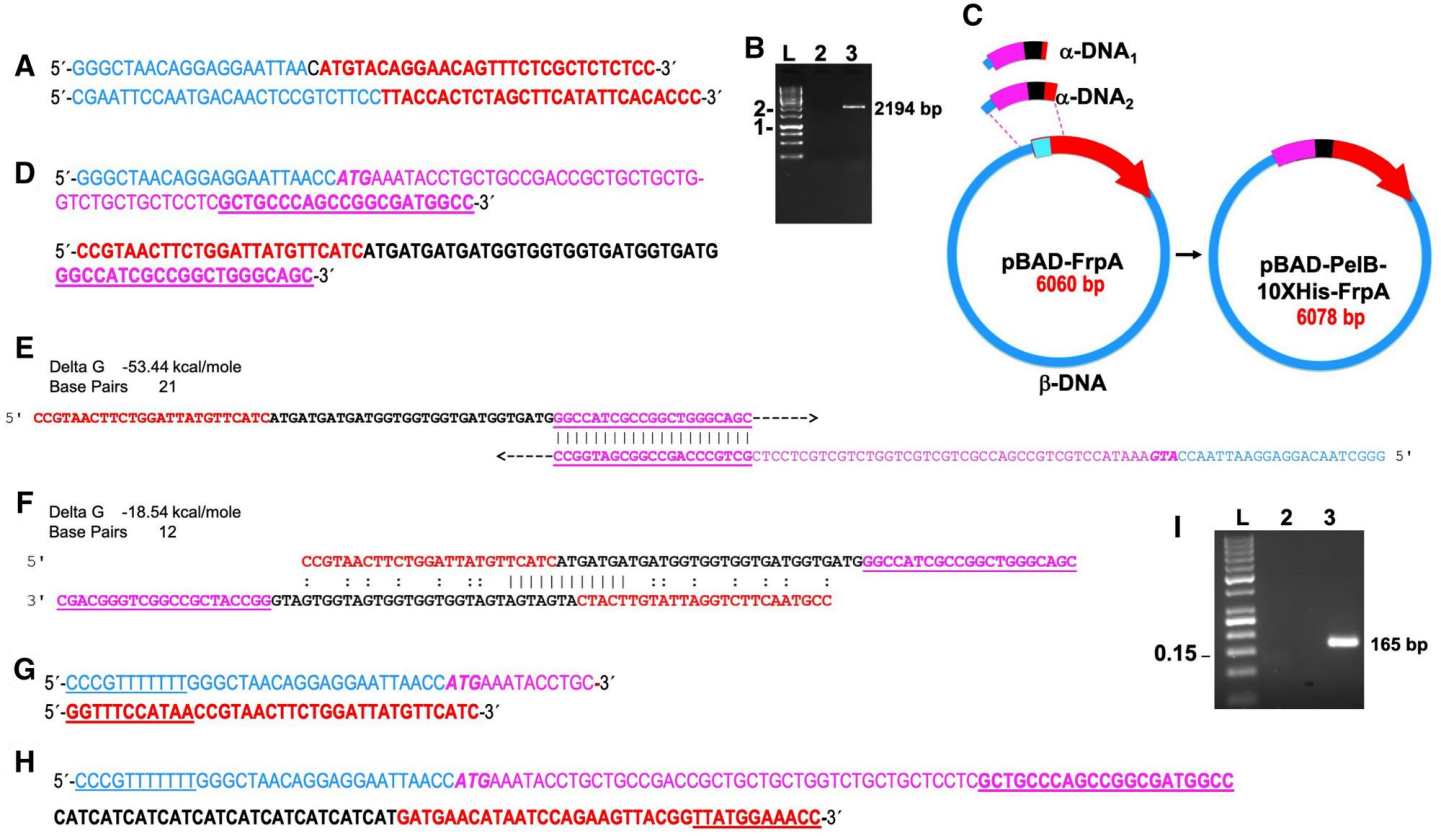
Primer design for the construction of pBAD-pelB-10XHis-FrpA. (A) Primers Fwd-frpA-pBAD (top) and Rev-frpA-pBAD (bottom). Sequences with homology to pBAD are highlighted at the 5’-ends. Sequences with homology to frpA are highlighted at the 3’-ends. (B) Electrophoresis analysis of colony-PCR products. Lane 2, no-polymerase control; lane 3, 2194-nt product. (C) Schematic view of α- and β-DNA in a Type II CiPCR for simultaneous insertion/deletion (CiPCR10). Two α-DNAs are shown, α-DNA_1_ and α-DNA_2_, displaying their regions of homology to β-DNA and the regions to be inserted. FrpA, thick arrow in the β-DNA; FrpA’s signal sequence, thick stripe preceeding the arrow in the β-DNA; PelB coding sequence, thick stripe on the left of α-DNA_1_ and α-DNA_2_; 10H tag, thick stripe following PelB coding sequence; thick stripe on the right of α-DNA_1_ and α-DNA_2_, region of homology to FrpA in the β-DNA; region with homology to pBAD, thin stripe on the left of α-DNA_1_ and α-DNA_2_. The direction of transcription of the fusion gene is indicated by arrowhead. (D) Primers F-pBAD-pelB (top) and pelB-His-FrpA-R (bottom). Region with homology to pBAD is highlighted in the 5’-end in top primer. PelB sequence is shown following the homology to pBAD region, including the underlined part in the 3’-end. Sequence encoding the 10H tag is shown in bold font in the center of the bottom primer. Sequence with homology to FrpA is highlighted on the 5’-end. The region of terminal homology between Primer F-pBAD-pelB and pelB-His-FrpA-R is underlined and in bold font. The position of the 5′- and 3′-ends is indicated. The same format applies to the following panels. (E) Expected annealing for primers F-pBAD-pelB and pelB-His-FrpA-R. Thin arrows denote the direction of extension by polymerase. (F) Self-dimer of pelB-His-FrpA-R. (G) Primers Reamp forward (top) and reverse (bottom). (H) Expected sequence of α-DNA following amplification with Reamp primers. A single strand is shown. Underlined sequences denote the newly added regions, increasing homology to pBAD-FrpA(wt). (I) Electrophoresis analysis of PCR products. Lane 2, no-polymerase control; lane 3 165-nt product. Molecular weights in bp are indicated in (B) and (I, lane 3) and in kbp in (B, lane L) and (I, lane L).

The outer membrane protein FrpA is a 660 amino acid protein that functions as an iron transporter in the fish pathogen *P. damselae* subsp. *piscicola* DI21. Our goal was to over-express FrpA for the generation of vaccines against *P. damselae* subsp. *piscicola* DI21. To do this, we cloned the *P. damselae* subsp. *piscicola* DI21 *frpA(wt)* gene, encoding FrpA(wt), in the expression plasmid pBAD [42]. It was important to isolate FrpA(wt) in its native conformation, which requires its transport to the outer membrane for proper folding. One of the plasmids created at the initial stages of the project was pBAD-FrpA(wt), carrying the entire sequence of the *frpA(wt)* gene directly under the control of the P_BAD_ promoter. The *frpA(wt)* gene was amplified by colony-PCR from a culture of *P. damselae* subsp. *piscicola* DI21, with the pair of chimeric primers frpA-pBAD (Table 1, Fig. 10A). These primers contain 5′-terminal homology to a region of the pBAD that would place the *frpA(wt)* CDS under the control of the P_BAD_ promoter. As expected, colony-PCR with these primers yielded the 2194-nt product containing the entire sequence of *frpA(wt)* (Fig. 10B). pBAD-FrpA(wt) was constructed by insertion of the *frpA(wt)* sequence in pBAD by means of a Type II CiPCR (CiPCR9) with the product of PCR9 as α-DNA and pBAD as β-DNA (data not shown). In these initial stages of the project, we also attempted to express a his-tagged version of the entire FrpA(wt), carrying FrpA(wt)’s native signal sequence (data not shown). Failure to achieve high levels of His-tagged r-FrpA(wt) in these initial trials (data not shown), prompted us to replace FrpA(wt)’s native signal sequence with the PelB signal from *Erwinia carotovora*, which has been successfully used for the expression of outer membrane proteins in *E. coli* [54, 55]. In order to replace FrpA(wt)’s native signal sequence with the PelB signal from *E. carotovora*, we first identified FrpA(wt)’s putative 26-amino-acid signal sequence by using the SignalP 4.1 Server [56]. Following the design of Yue *et al*. [55] who successfully over-expressed the *E. coli* iron transporter FecA, following pelB, we sought to add a 10H tag for affinity purification. Hence, our goal was to build a fusion protein carrying N-terminal PelB, followed by 10H, and the sequence of FrpA without amino acids 1-26, FrpA(p.1_26del). To do this, we attempted to delete amino acids 1–26 from pBAD-FrpA(wt) (to be used as β-DNA), concomitantly to the insertion of the sequence encoding PelB-10H. We designed an α-DNA (α-DNA_1_ in Fig. 10C) carrying the 25-nts of pBAD-FrpA(wt) sequence preceding and including the inducible start codon, followed by the sequence encoding PelB fused to 10H and by an additional 25 nts corresponding to the coding sequence of FrpA(wt) starting at codon 27. To build α-DNA_1_ (143 nts) (Fig. 10C) we designed two long primers, the 88-mer F-pBAD-pelB (Fig. 10 D (top)) and the 76-mer pelB-10H-FrpA-R (Fig. 10D (bottom)), which contained a 21-nt region of 3′-terminal homology to allow their hybridization (Fig. 10E). The primers were analyzed with the IDT SciTools web server [44], which predicted a total decrease in ΔG of −53.44 kcal/mole for the expected annealing product (Fig. 10E). Single-cycle extension of primers F-pBAD-pelB and pelB-10H-FrpA-R with Phusion DNA polymerase failed to yield the expected 143-nt dsDNA product, as judged by electrophoresis analysis (data not shown). This failure was possibly explained by the formation of undesired annealing intermediates, as predicted by IDT SciTools (Fig. 10F). Despite this, we suspected that α-DNA_1_ was still generated, albeit at levels too low to detect by electrophoresis. To check this possibility, we designed the pair of primers Reamp, capable of amplifying α-DNA_1_, were it indeed in the single-cycle extension reaction mixture. The sequence of the pair of primers Reamp is shown in Fig. 10G. Both primers contained long regions of homology to α-DNA_1_ at their 3′-ends and carried an extra 11 nts on their 5′-ends, effectively increasing 5′-terminal homology relative to pBAD-FrpA(wt) (β-DNA) (Fig. 10G). These primers were used in a regular PCR (PCR10) to amplify the single-cycle extended products, giving rise to the expected 165-nt α-DNA_2_ (Fig. 10H–I). α-DNA_2_ was used directly in a Type II CiPCR intended for simultaneous insertion/deletion, together with pBAD-FrpA(wt) as β-DNA (Fig. 10C). The resulting plasmid was named pBAD-pelB-10XHis-FrpA (Fig. 10C). Expression assays with this plasmid and proof of the proper folding of the protein at the outer membrane have been reported elsewhere [43].

### Random SDM by Type II CiPCR

The following is an example of random SDM by Type II CiPCR. In addition, a restriction-enzyme-based strategy for the quick identification of mutant plasmids is provided.

**Figure 11.**
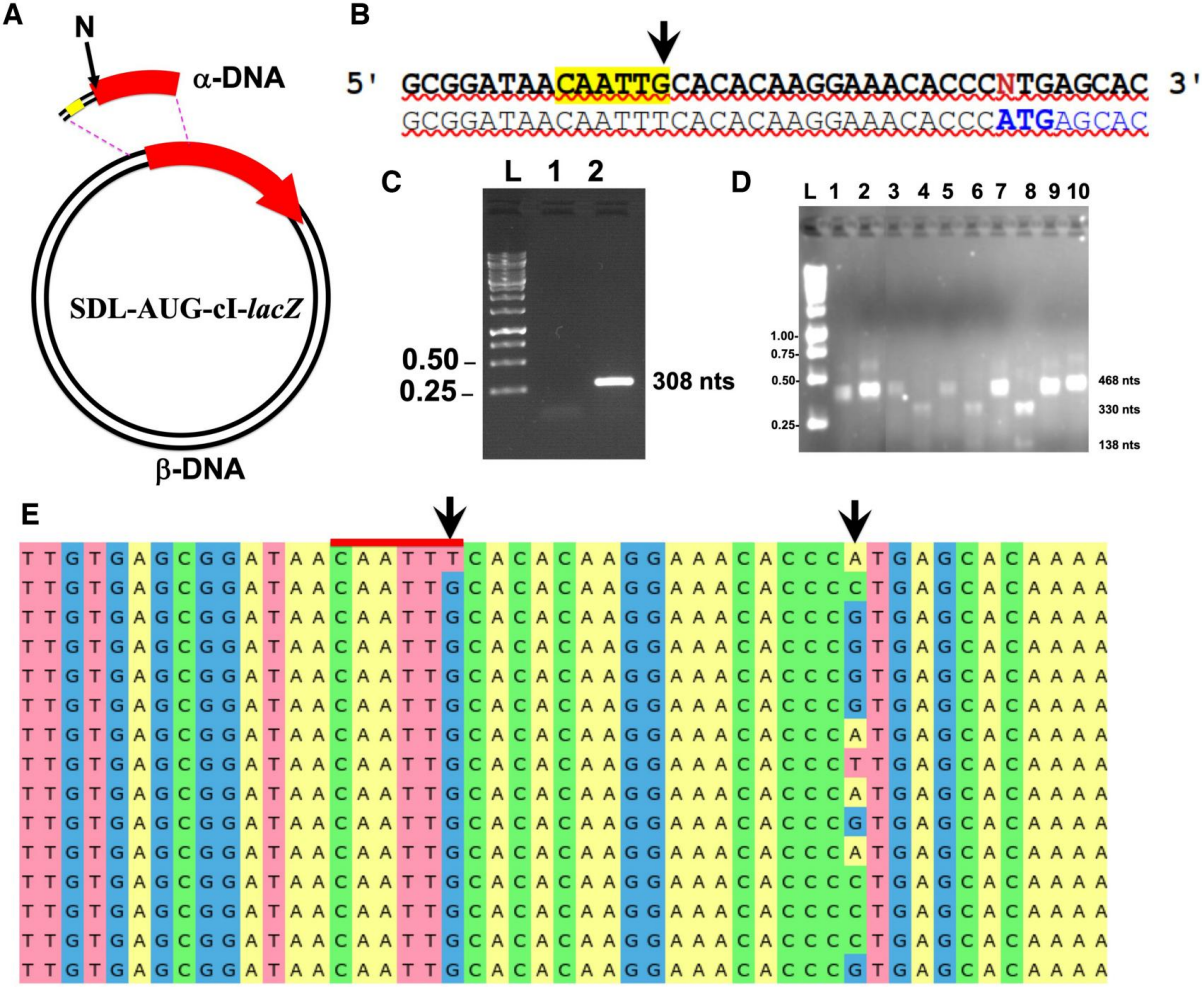
Primer design for the construction of the plasmid series SDL-+1N. (A) Schematic view of α- and β-DNAs in a Type II CiPCR for simultaneous insertion/deletion (CiPCR11). LacZ-based reporter, thick, stripe in the α-DNA and thick arrow in the β-DNA. N, randomized position. Thin stripe in the left of α-DNA, MunI restriction site. (B) Design of primer +1 N. Randomized mutagenic position is indicated with an N close to the 3’-end. The MunI restriction site is highlighted at the left side of the T > G mutation (position 14, indicated with an arrow). The original sequence of plasmid SDL is shown underneath with coding sequence and ATG start codon in bold font. (C) Electrophoresis analysis of α-DNA. Lane 1, no-polymerase control; lane 2, 308-nt product. (D) Electrophoresis analysis of colony-PCR-generated fragments, following transformation with CiPCR11 products. Lane 1: SDL, uncut; lane 2: SDL, MunI; lane 3: colony 1, uncut; lane 4: colony 1, MunI; lane 5: colony 2, uncut; lane 6: colony 2, MunI; lane 7: colony 3, uncut; lane 8: colony 3, MunI; lane 9: colony 4, uncut; lane 10: colony 4, MunI. Molecular weights in kbp are indicated in C and D for lanes L, and in bp in lanes 1-10. The position of the expected 468-bp, colony-PCR fragment and the 138- and a 330-bp MunI fragments are indicated. (E) Sequence analysis showing the sites of mutation. Top sequence, reference; other sequences, mutants. Stripe underneath the left arrow, MunI restriction site. Right arrow, randomized mutagenic position The alignment was performed with the Clustal-O multiple-alignment tool of UGENE [85, 86].

The SDL plasmid construct, carrying a derivative of the *E. colilacZ* gene that encodes the reporter enzyme β-galactosidase, has been reported [39, 40]. We have used this construct to measure β-galactosidase activity as proxy for the rate of translation initiation by *E. coli* ribosomes [40]. One of the most important determinants of translation initiation rates is the sequence of the start codon (position +1) [43]. To modify, the sequence of position +1 of the lacZ-based reporter carried in SDL, we have designed a CiPCR strategy. The, so called, SDL-+1 series of constructs, carrying all four bases at position +1 of the lacZ-based reporter, will enable us to measure the influence of this position on the rates of translation initation. Fig. 11A shows a schematic view of this Type II CiPCR. Fig. 11B (top) shows the sequence of the mutagenesis primer +1 N (39-mer, Table 1) indicating the location of the randomized position +1 (red N, N = A, C, G, T) (Fig. 11A). The sequence of primer +1 N carried one additional modification, relative to the original SDL sequence, namely a T to G base change at position 14 that created a MunI restriction site (highlighted in yellow in Fig. 11A and B). This restriction site would later be used to select transformants carrying the recombinant plasmid. Following amplification of SDL with primers +1 N and Sec-lacZ-R (Table 1), the latter binding 255 nts downstream of position +1 (PCR11), a 308-bp α-DNA was obtained (Fig. 11A and C). To generate the SDL-+1 series of constructs, carrying all possible bases at position +1, we used this α-DNA in a Type II CiPCR, together with the original plasmid SDL as β-DNA (CiPCR11). Colony-PCR analysis of the transformants, followed by MunI digest, was used to detect the mutant constructs (Fig. 11D). All four possible bases were verified by DNA sequencing analysis (Fig. 11E).

## Discussion

### CiPCR is a simple, powerful, and highly versatile molecular cloning technique

The increasing need for new genetic constructs to be used in scientific studies and in medical applications has led to the introduction of many kinds of commercial products and solutions that are often quite expensive. The modern molecular biology laboratory aspires to invest less time and money in obtaining such genetic constructs. CiPCR is a highly versatile tool that meets these needs by allowing the seamless fusion of any two fragments of dsDNA as long as they present the required homology. When performed with dsDNA fragments carrying bacterial vector elements, nicked CiPCR products can be directly transformed into *E. coli* hosts, giving rise to intact plasmids (Fig. 2O). Molecular cloning, SDM, and the induction of deletions and inversions (untested) are possible using CiPCR. The generation of new DNA constructs by CiPCR merely requires simple presence of appropriate regions of homology in α- and β-DNAs.

In this work, we have demonstrated that CiPCR is a swift, highly versatile, and cheap DNA manipulation tool that can be used for almost all molecular cloning applications. At least 57 CiPCR were successfully performed in our laboratory and in that of our collaborators in a period of about three years. Of these, approximately 26 were intended for SDM, with 1 case of random SDM; 26 for the introduction of insertions; 3 to generate deletions; and 1 for the simultaneous generation of an insertion and a deletion. Both Type I and Type II CiPCR schemes were successfully used with α-DNAs ranging from 25 to approximately 2.400 bp, either fully synthetic or obtained via PCR amplification. We have also shown that many different types of sequences can be added to α-DNAs for their incorporation to the final CiPCR nicked circular products. Such sequences included short elements such as HA, 10H, EGFP, and GST tags or the preScission (PS) and the enterokinase (EK) target sequences (Table 3) or the pelB signal peptide; entire CDSs such as those of *mTmprss*, *HIST4H4, PCBP1*, *KLF9*, *SP1*, *SLC16A2*, *frpA*, and *THRβ* (Table 3). We have also succeeded in using CiPCR to modify expression of genes by using α-DNAs with modified promoter regions, silencer sequences, or with removed stop codons. We also used at least six different plasmids as β-DNA (Table 4).

Among all the presented examples, perhaps the two most important are shown in Fig. 4. In this Figure (together with the DNA sequencing analysis of Supplementary Fig. S1), our prediction that Type I and II CiPCR methods must give rise to the same circular DNA products was confirmed, by showing that the two methods gave rise to exactly the same circular DNA molecules, as expected. Hence, the user can freely choose which of the two CiPCR methods is more suitable for a particular protocol, depending in part on the nature of the starting materials.

For most CiPCR protocols we described, we demonstrated the ability of the constructs to express the intended recombinant products. In the particular case of pcDNA3-hSLC16A2(wt)-EGFP, expressing a C-terminal, EGFP tagged version of MCT8(wt), MCT8(wt)-EGFP, the evidence for its proper expression is twofold. First, the strong fluorescent activity of the EGFP portion of the fusion protein is shown in Fig. 4I. Second, evidence of MCT8 activity was provided by the staining pattern afforded by the fusion protein. MCT8 belongs to the MCT family, known to insert into cellular membranes by means of twelve transmembrane domains [57–59]. While some of the MCT family members require aid from chaperone proteins for correct insertion into the plasma membrane, MCT8 does not [60]. Regarding the correct membrane localization of MCT8 variants carrying N- and C-terminal tags, numerous articles have shown that this ability is not affected by the extensions [61–66]. With this in mind, the staining pattern observed in panel c of Fig. 4I, namely diffuse perinuclear-cytoplasmic staining accompanied by strong membrane staining, is highly reminiscent of results reported by others [66]. Since the EGFP portion of the fusion protein lacks membrane localization signals [67, 68], it is clear that the ability to generate the correct staining pattern, must be provided by the MCT8 fragment.

Besides the correct localization of MCT8-EGFP, we functionally demonstrated the correct expression of another multi-tagged, fusion protein, namely 10H-GST-PS-10H-EK-hPCBP1(wt), which was expressed from p2GUS-10H-GST-PS-10H-EK-hPCBP1 (see Fig. 6). In the case of pSUPER.neo-Si-SLC16A2, encoding a gene silencing construct, we demonstrated its functionality by transfection assays decreasing the levels of expression of endogenous h*SLC16A2* mRNA (Fig. 5I). We also used luminescence assays to show the functional effect of a deletion in the promoter region of a gene (see Fig. 7). Finally, one particularly difficult project involved the construction of a recombinant version of a bacterial membrane protein (FrpA) (see Fig. 10). We successfully used CiPCR to build pBAD-pelB-10XHis-FrpA and demonstrated the correct expression of the intended recombinant protein, as reported elsewhere [43].

The rate of CiPCR success on the first try is about 90% and in cases were failure was observed, redesigning α-DNAs, as mentioned above (described in Figs 9 and 10, and see also Supplementary data), often solved the problem. Hence, CiPCR is a highly efficient cloning tool whose rate of success can be compared at least to that of the traditional restriction-ligation method with the difference that CiPCR can be performed quicker when compared to restriction-ligation procedures used for complicated cloning schemes. From the point of view of the cost of CiPCR, only a single enzyme is required in Type I with linear β-DNAs and two in Type II when plasmid DNA is used as β-DNA. Hence this technique is highly affordable, providing a highly cost-efficient solution for most molecular cloning applications encountered by biology laboratories.

Hence, CiPCR ensues that performing basically any type of molecular cloning procedure comes down to simply designing compatible α- and β-DNAs, preparing these precursors, performing the CiPCR, and transforming the products into an appropriate host. The entire process can be accomplished in one day, and the second day transformants are ready for screening. Its simplicity at the drawing board and swiftness in the wet lab, together with its high rate of success, makes this procedure an affordable alternative for most applications requiring molecular cloning.

### Additional theoretical considerations

**Figure 12.**
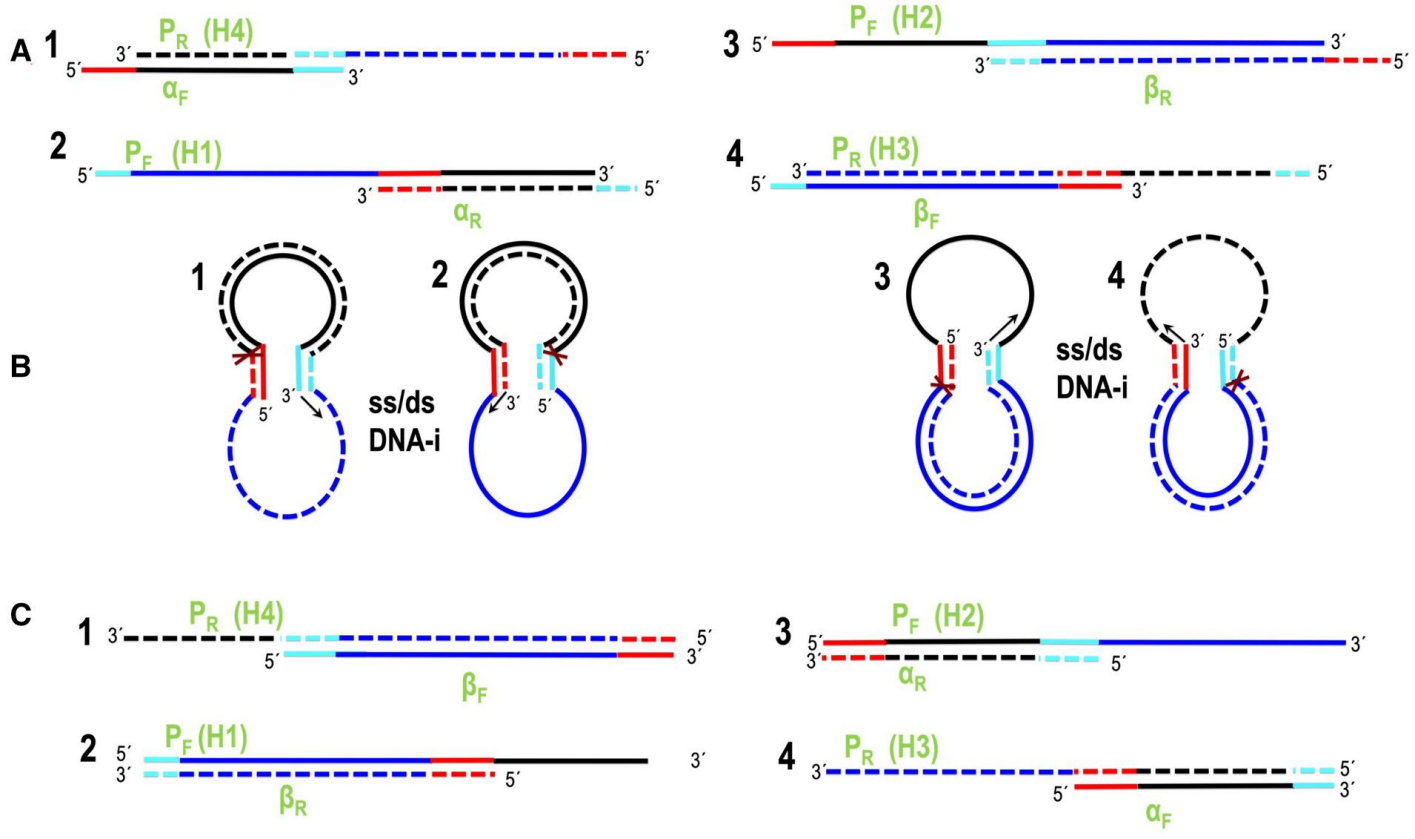
Templating by CiPCR products. (A) Type I CiPCR forward- and reverse-product strands (P_F_ and P_R_) can anneal with complementary α- or β-strands (α_F_ and α_R_, β_F_ and β_R_). (B) Ss/dsDNA-i stage following the annealing arrangements shown in (A) Arrows denote extension at the available 3′-ends. Crosses indicate nicks. (C) Non-productive annealing. Strand names relative to the CiPCR products shown in Fig. 2H and M are provided in parentheses for clarity in (A) and (C).

Beyond the mechanistic differences between Type I and Type II CiPCR noted in the previous section, we hypothesize that there must exist important kinetic differences. For example, after the first Type I CiPCR cycle, the annealing of ss CiPCR products with complementary α- or β-strands (Fig. 12A) could potentially lead to *ss/dsDNA-i* complexes with single extendable 3′- ends (Fig. 12B). Extension of these 3′-ends (Fig. 12B) by product templating, as in regular PCR, would yield doubly nicked dsDNA molecules identical to the final CiPCR products shown in Fig. 2I. Note that only half of the annealing products formed between ss Type I CiPCR products with complementary α- or β-strands, namely those shown in Fig. 12A, could lead to productive complexes, containing extendable 3′-ends, at the *ss/dsDNA-i* stage. The other half are expected to result in non-productive intermediates (Fig. 12C). Neither of the two ss products expected for Type II CiPCR (Fig. 2M) is expected to result in productive complexes by this mechanism (Fig. 12C).

While CiPCR byproduct formation is minimized by the use of polymerases without strand displacement activity, low template concentrations, and the use of few amplification cycles (between 10 and 20), their presence is expected. The detection of smears on electrophoresis gels (Fig. 4G), clearly reveals the production of a wide range of byproducts that could interfere with the efficiency of the procedure. Some of these byproducts could be the result of the aberrant annealing between the α- and β-DNA strands present in the mixture (e.g. Fig. 3A). Byproducts of this type, giving rise to circular dsDNAs with staggered nicks (e.g. Fig. 3B and C) are expected to form readily but will yield unstable plasmids with more than one origin of replication. Byproducts with single origins, but with more than one copy of α-DNA are also possible but have not been detected by either iPCR or following transformation. At any rate, the presence of such byproducts should be easily detected by restriction analysis following transformation.

The large amount of homology presented by the substrates and CiPCR products could also give rise to unnicked continuous ssDNA strands longer than the predicted product shown in Fig. 2G and H. Some combinations of such ssDNA strands could result in the large, circular, nicked dsDNA molecules shown in Fig. 3K. Our iPCR results failed to detect such large, continuous strands. These results must be taken with caution, however, as it is possible that the presence of many different types of CiPCR products with large homology could just inhibit iPCR amplification. The formation of larger nicked concatemers by self-annealing of the products D, E, F, H, or L shown in Fig. 3 is also possible. In fact, we have detected this type of CiPCR byproducts larger than the canonical ones in the Type II CiPCR with 19 amplification cycles (Fig. 4G, lanes 2–4), which agrees with the results published by other authors [69]. The formation of these products may become particularly relevant after 18/20 cycles [69]. Although additional research must be conducted to begin to understand the kinetics of product and byproduct formation during CiPCR, we do not recommend using more than 20 cycles.

Clearly, the formation of undesired byproducts is much more probable in Type I than in Type II CiPCR. As a result, the idea that Type I CiPCR might operate with exponential kinetics, as suggested by the possibility that the target ssDNA intermediates (Fig 12A-B, H1, H2, H3 and H4) can function as templates during subsequent amplification cycles, is expected to be hampered by two highly interconnected issues, namely the pervasive trapping of CiPCR intermediates in non-productive byproducts and the existence of many types of potential byproducts. In the case of Type II CiPCR, the maximum rate of product formation should be much less affected by the formation of undesired byproducts, albeit it is expected to reduce the maximum amplification rate afforded by linear kinetics. In the absence of proper kinetic assays aimed at measuring the actual rates of Type I and Type II CiPCR, here, we merely attest that both techniques are capable of producing sufficient material to successfully resolve most problems normally encountered during the creation of the type of DNA constructs often used in molecular biology with an efficiency that is comparable, if not superior, to that of the traditional restriction-ligation methods. The elucidation of the actual CiPCR kinetics is an important issue to be resolved by future investigation. A clear understanding of this matter might be required in order to extend the use of CiPCR-based techniques to tasks lying beyond the realm of host-dependent, molecular cloning techniques (see below).

### Fit of CiPCR in the realm of molecular cloning techniques

**Figure 13.**
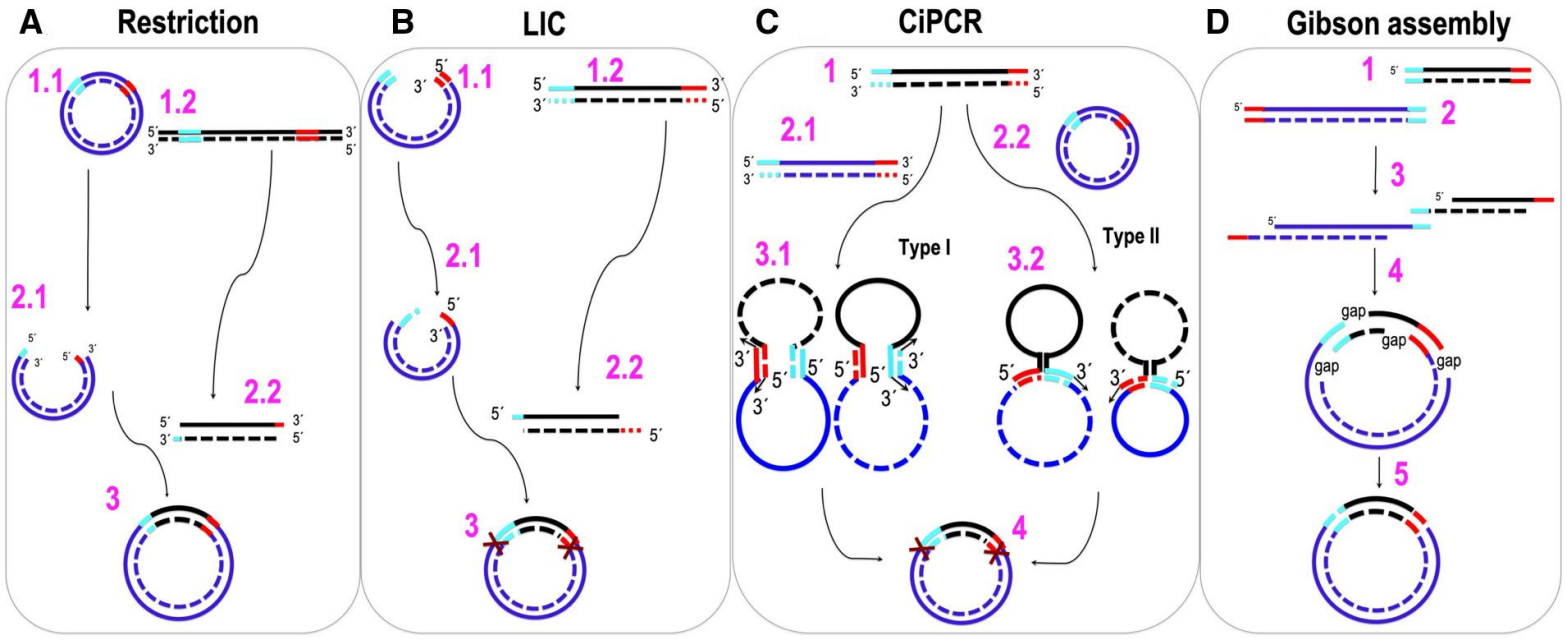
Methods of overhang generation for molecular cloning. (A) Restriction-ligation cloning. Steps 1.1 and 1.2, vector (1.1, circular) and insert (1.2, linear), with appropriate restriction sites (highlighted regions). Step 2.1, vector following restriction digest. Step 2.2, insert following restriction digest. Step 3, ligated product. (B) LIC, original design. Steps 1.1 and 1.2, PCR amplification of vector (1.1) and insert (1.2). Steps 2.1 and 2.2, creation of 3′-overhangs by the (3′–5′) exonuclease activity o T4 DNA polymerase. Step 3, construction of circular dsDNA molecules with staggered nicks following the annealing of the linear products of Steps 2.1 and 2.2. (C) CiPCR. Step 1. α-DNA. Steps 2.1 and 2.2 β-DNAs for Type I (2.1) and Type II (2.2) CiPCR. Steps 3.1 and 3.2, ss/ds DNA-is for Type I (3.1) and Type II (3.2) CiPCR. Step 4, circular dsDNA with staggered nicks. (D) Gibson assembly. Steps 1 and 2 PCR amplification of DNA1 (1) and DNA2 (2). Isothermal step 3-5: T5 exonuclase reaction to create 5′-overhangs (indicated as 3); overhangs annealing (indicated as 4); gap filling by a polymerase to generate circular dsDNA with staggered nicks, repaired by a ligase (indicated as 5). DNA strand in the 5′–3′ direction, solid line, DNA strand in the 3′–5′ direction, broken line. The position of 5′- and 3′-ends is indicated.

Several other methods for molecular cloning exist that employ different types of sticky overhangs (summarized in Fig. 13). Some methods are of general use and others are more appropriate for particular applications. Similarly, they range from simple and cheap, to laborious and expensive. Restriction-ligation cloning [3–5] was the first of such methods and it uses short overhangs, often just 4 nts, generated by restriction enzymes. Its main disadvantage is the requirement of restriction enzyme compatibility that limits its use. Moreover, since the short overhangs cannot be used to stabilize plasmid DNA in a circular configuration, *in vitro* ligation is obligatory. Finally, this method is not seamless, as the use of restriction enzymes with defined target sites may lead to the presence of “scars” following ligation.

The search for cloning methods more versatile than restriction-ligation cloning, led to the development of LIC methods, starting in the 1990s. The original LIC method functioned by the generation of 12–15-nt complementary overhangs in the vector and insert by PCR-amplification, by using carefully designed primers carrying extensions at their 5′-ends (steps 1.1 and 1.2 in Fig. 13B) [9]. The 3′-end portion of the extension was removed by T4 DNA polymerase (steps 2.1 and 2.2 in Fig. 13B), prior to the annealing of vector and insert to form a circular dsDNA molecule with staggered nicks (step 3 in Fig. 13B). While the original LIC method was dependent of certain base composition for the overhangs, thus making it not sequence independent [9], more versatile versions rapidly were reported [10, 12, 70]. One such methods, named RCPCR (recombinant circle PCR) has been used for SDM and cloning [71]. When used for SDM, RCPCR requires two iPCRs performed separately, each with one member of a pair of complementary, mutagenic primers, together with a secondary primer targeted to a site immediately upstream of the mutagenic oligo [71]. Following the iPCRs, the dsDNA, linear products are mixed, giving rise to circular dsDNA molecules with two staggered nicks. Hence, the RCPCR, when used for SDM, is akin to our CiPCR. Cloning by RCPCR, however, is cumbersome, as it requires the amplification of vector and insert with four different primers each, to generate four dsDNA linear products presenting offset regions of homology. Upon mixing and annealing, these products yield circular dsDNA molecules with four staggered nicks [71]. We have shown that half of the primers can be avoided with our Type I CiPCR design and three quarters with Type II.

The use of iPCR allowed the introduction of mutations at any site in circular plasmids by means of mutagenic primers and the use of a ligase [72]. This idea was later adapted into a “LIC” method akin to Type II CiPCR [73], that was eventually commercialized as the QuikChange™ Site Directed Mutagenesis kit (Stratagene). This method, leading to the construction of circular dsDNA molecules with two nicks, was the inspiration for the establishment of the CiPCR hypothesis presented here. In fact, some of the examples included here under the CiPCR umbrella (Fig. 8) are nothing but mere variations of the original iPCR-based, “LIC” method for SDM. This idea was soon extended to the construction of multiple mutations, or to induce site-directed mutations in large plasmids [74, 75]. OE-PCR, was invented in 1988 to introduce site-directed mutations by the union and amplification of two linear fragments [24, 71] that could be subsequently cloned into a vector. An adaptation of this method to the insertion of dsDNA into plasmids, namely the OE-PCR cloning method [69], was soon reported. Since OE-PCR cloning is a Type II CiPCR identical to the one described in Fig. 2J–N, it should be also classified under the umbrella of CiPCR methods. Despite this, there are slight differences between the OE-PCR cloning method and our Type II CiPCR examples. While for OE-PCR cloning the authors recommended the use of Phusion DNA polymerase and the generation of somewhat long regions of homology, 30–40 nts [69], we found that Takara DNA polymerase performed better than Phusion DNA polymerase and demonstrated that regions of homology as short as 12–20 nts usually behaved satisfactorily.

The long-fragment circular-efficient PCR (LC-PCR) of Jailani *et al*. [76] is another variation of OE-PCR that is capable of effectively modifying vectors as large as 16.4 kb. Notably, in all the LC-PCR examples the authors added an extra primer that would be rendered unnecessary had they designed their α- and β-DNAs according to either Type I or II CiPCR. This raises the interesting question of whether CiPCR can be efficiently applied to long vectors. The largest vector used in a truly CiPCR experiment had a size of 13 kb [74]. We believe that since techniques for the amplification of large vectors are rapidly improving, CiPCR will be proven useful for the modification of vectors with sizes larger than 13 kb.

Shao et al reported the so-called *in situ* error-prone PCR (is-epPCR) to generate DNA libraries carrying random mutations [77]. Their method is similar to CiPCR in that a dsDNA linear fragment carrying an entire empty vector is used to prime the amplification of a target gene carried on another circular vector. Homology between the linear fragment and the circular vector is complete, except for the resistance marker and target gene. Amplification with an error-prone DNA polymerase instead of a high-fidelity polymerase yielded random mutations only in the target gene. The procedure requires the use of phosphorylated primers and a thermostable DNA ligase to ligate all nicks, prior to transformation. Our results and those of others [69], demonstrating that ligases and phosphorylated primers are not necessary for cloning, strongly suggest that is-epPCR could be simplified by adapting it to a Type II CiPCR design.

Another interesting OE-PCR-based technique is ABC cloning [78]. The authors used three gel-purified dsDNA fragments instead of the two used in Type I CiPCR, the so-called B fragment, carrying the dsDNA insert, and the overlapping A and C fragments, covering the entire vector pET28a (5369 bp) [78]. Hence, to generate the precursor DNAs, they use three regular PCRs, followed by gel extraction for each product. The mixing of the three fragments, together with two complementary primers designed for iPCR, resulted in the amplification of the intended recombinant construct. Our results demonstrate that the same results can be achieved with a much simpler design composed of only two fragments and without gel extraction. Notably, the ABC three-fragment scheme might prove useful for the extension of CiPCR to long plasmids (see below).

One excellent method for DNA manipulation is Gibson assembly. This non-PCR-based method uses T5 exonuclease to create random-length overhangs that can be used for the joining and circularization of dsDNA products [17] (Fig. 13). The assembly is performed in a single tube under isothermal conditions. Originally, a DNA polymerase and a ligase were included in the reaction to fill gaps and seal nicks, respectively. Later, LIC versions of this method were also described [79]. The great advantage of Gibson assembly is the length of the dsDNA constructs that it can build [80].

Another cloning method that uses overhangs of random length is FastCLoning [81]. In this case, only a high-fidelity DNA polymerase is used to separately amplify the vector and the insert with primers designed to create terminal homology between the two dsDNA fragments. Following their mixture and Dpn I treatment to destroy the templates required, the protocol leads to the creation of a circular construct with nicks, supposedly by the generation of sticky ends in the regions of homology. Although the mechanism for the creation of sticky ends is unknown, the authors cite the possibility that such ends are the result of the 3′-exonuclease activity of the DNA polymerase acting on the terminal homology regions during DpnI digestion [81].

Not surprisingly, these two methods relying on sticky overhangs are afflicted by the same issue, namely undesired mutations. Gibson assembly is reported to produce ∼1 error per 50 DNA molecules joined [17], whereas FastCloning produces errors in up to 5% of the clones [81]. According to the FastCloning authors, the sequences across the primers are particularly sensitive, a fact that they explain by the presence of unwanted variation in the sequences of a small fraction of the primers [81]. In contrast to these claims, we have not yet detected a single undesired mutation with CiPCR.

As mentioned above, the CiPCR hypothesis was motivated by LIC iPCR-based methods intended for SDM. At the same time, some of the methods classified as OE-PCR, namely OE-PCR cloning, fall within the scope of the CiPCR definition, more specifically as a Type II CiPCR cloning method. Hence, the CiPCR mechanism overlaps with those of iPCR and OE-PCR. It is important to note that iPCR was originally designed to amplify plasmid DNA into linear dsDNA molecules [23]. Similarly, OE-PCR was originally intended for the construction of linear dsDNAs (see Fig. 1), and, for these reasons, we believe that neither iPCR nor OE-PCR are appropriate terms to define the group of PCR-based methods that yield circular dsDNA products with staggered nicks. This distinction prompted us to propose the CiPCR name to include our Type I and II methods, as well as potential derivations thereof that result in circular dsDNA products with staggered nicks.

Considering the CiPCR examples available in the literature, along with those presented here, we have shown that the CiPCR hypothesis gives rise to a series of methods that successfully address most of the molecular cloning challenges customarily faced by biology/biomedicine laboratories. Most importantly, our mechanistic explanation for the functioning of CiPCR permitted the distinction between Type I and Type II CiPCR, with the former being formally demonstrated here for the first time as a cloning technique, as far as we know. While Type I CiPCR can, in principle, perform all the same tasks as Type II CiPCR, the latter is usually preferred for cloning for two reasons. First, it produces fewer byproduct species and likely smaller quantities of each due to its slower kinetics. Second, plasmid DNA can be used directly in the reaction as a template. However, the fact that Type I CiPCR leads to the construction of circular dsDNA with staggered nicks using linear dsDNA as a precursor, makes this technique a potential host-independent cloning method. In other words, Type I CiPCR is ideal for constructing any circular dsDNA molecule, regardless of whether it carries the sequences required for its own maintenance in the host. For example, Type I CiPCR could be particularly interesting for the construction of next-generation DNA vaccines [82, 83]. DNA-based vaccines are expected to be less sensitive to storage, handling, and shipping conditions than RNA- or protein-based vaccines [84]. For some DNA vaccines, avoiding the inclusion of sequences derived from the bacterial amplification system is a requirement [83]. In this case, nick reparation via passage through a bacterial host is no longer possible. Currently, the construction of such circular DNA molecules has been achieved by the construction of DNA minicircles starting from plasmid DNA [83]. This is a complicated method involving recombination, followed by purification of the resulting recombinant DNA minicircles [83]. We believe that Type I CiPCR could be used to obtain DNA minicircles by coupling the construction of dsDNA-staggered nick minicircles with nick ligation using a thermostable ligase. A better understanding of the kinetics of Type I CiPCR, both during target product and byproduct formation, will be likely required to optimize the output of this technique. Besides DNA vaccines, we envision that the creation of this type of circular DNAs could also be of interest in the fields of gene therapy and cancer immunotherapy.

## Supplementary Material

bpae051_Supplementary_Data

## Data Availability

All data and constructs described in this article will be shared upon reasonable request placed to the corresponding author.
