# Supplementary material for: Circular PCR as an efficient and precise umbrella of methods for the generation of circular dsDNA with staggered nicks: Mechanism and types
Source: Biol Methods Protoc. 2024 Aug 12;9(1):bpae051. doi: 10.1093/biomethods/bpae051 (PMC11319657; doi:10.1093/biomethods/bpae051)
Supplement: bpae051_Supplementary_Data [file bpae051_supplementary_data.docx]

**MANUSCRIPT TITLE**

**Circular PCR as an efficient and precise umbrella of methods for the generation of circular dsDNA with staggered nicks: mechanism and types**

# Supplementary Information

1. **Example of a practical procedure for the design of chimeric primers for CiPCR to construct the plasmid pcDNA3-SLC16A2-EGFP.**

***First.*** Choose the position to insert your dsDNA of interest, such as EGFP, in frame with the C-terminal region of the *SLC16A2* gene in the plasmid pcDNA3-SLC16A2(wt). To do this, write a small sequence around that position of insertion (shown by the black arrowhead) using the 5´-3´ oriented strand only (for simplicity) as shown below: blue belong to flanking sequences of the plasmid, the pink to the flanking sequences of the *SLC16A2* ORF and orange and cyan are the selected sequences flanking the insertion point of fragment EGFP:

5´….ccccattgcaggcctactccgcaactgttttggggactaccatgtggccttctactttgccggtgtgccccccatcatcggggctgtaatcctcttcttcgtccctctgatgcatcaaaggatgttcaagaaagagcagagagattccagcaaggataagatgttggcccctgacccagaccccaatggggagctactgccgggctcccccaaccctgaggaaccaatcTAAtgcctttcttgccattgtgtgctctagagggccctattctatagtgtcacctaaatgctagagctcgctgatcagcctcgactgtgccttctagttgccagccatctgttgtttgcccctcccccgtgccttccttgaccctggaaggtgccactcccactgtcctttcctaataaaatgaggaaattgcatcgcattgtctgagtaggtgt……3´

***Second*.** Now write the sequence of the 5´-3´ strand of the insert (black font: EGFP ORF insert) and add the sequences highlighted in orange and cyan in the above plasmid pcDNA3-SLC16A2 positioned as shown below (to introduce the homology sites, make sure the insert sequence remains in frame with the SLC16A2 ORF):

5´ccaaccctgaggaaccaatc**acaaccatggtgagcaa**gggcgaggagctgttcaccggggtggtgcccatcctggtcgagctggacggcgacgtaaacggccacaagttcagcgtgtccggcgagggcgagggcgatgccacctacggcaagctgaccctgaagttcatctgcaccaccggcaagctgcccgtgccctggcccaccctcgtgaccaccctgacctacggcgtgcagtgcttcagccgctaccccgaccacatgaagcagcacgacttcttcaagtccgccatgcccgaaggctacgtccaggagcgcaccatcttcttcaaggacgacggcaactacaagacccgcgccgaggtgaagttcgagggcgacaccctggtgaaccgcatcgagctgaagggcatcgacttcaaggaggacggcaacatcctggggcacaagctggagtacaactacaacagccacaacgtctatatcatggccgacaagcagaagaacggcatcaaggtgaacttcaagatccgccacaacatcgaggacggcagcgtgcagctcgccgaccactaccagcagaacacccccatcggcgacggccccgtgctgctgcccgacaaccactacctgagcacccagtccgccctgagcaaagaccccaacgagaagcgcgatcacatggtcctgctggagttcgtgaccgccgccgggatcactctcg**gcatggacgagctgtacaagTAA**tgcctttcttgccattg3´

***Third.*** Write the final sequence of the 5´-3´strand of the construct you want to obtain as shown below for pcDNA3-SLC16A2-EGFP: blue belong to the plasmid sequence; pink to the SLC16A2 ORF; black to the EGFP ORF and orange and cyan to the homology sites:

5´…..gccttctactttgccggtgtgccccccatcatcggggctgtaatcctcttcttcgtccctctgatgcatcaaaggatgttcaagaaagagcagagagattccagcaaggataagatgttggcccctgacccagaccccaatggggagctactgccgggctcccccaaccctgaggaaccaatc**acaaccatggtgagcaa**gggcgaggagctgttcaccggggtggtgcccatcctggtcgagctggacggcgacgtaaacggccacaagttcagcgtgtccggcgagggcgagggcgatgccacctacggcaagctgaccctgaagttcatctgcaccaccggcaagctgcccgtgccctggcccaccctcgtgaccaccctgacctacggcgtgcagtgcttcagccgctaccccgaccacatgaagcagcacgacttcttcaagtccgccatgcccgaaggctacgtccaggagcgcaccatcttcttcaaggacgacggcaactacaagacccgcgccgaggtgaagttcgagggcgacaccctggtgaaccgcatcgagctgaagggcatcgacttcaaggaggacggcaacatcctggggcacaagctggagtacaactacaacagccacaacgtctatatcatggccgacaagcagaagaacggcatcaaggtgaacttcaagatccgccacaacatcgaggacggcagcgtgcagctcgccgaccactaccagcagaacacccccatcggcgacggccccgtgctgctgcccgacaaccactacctgagcacccagtccgccctgagcaaagaccccaacgagaagcgcgatcacatggtcctgctggagttcgtgaccgccgccgggatcactctcg**gcatggacgagctgtacaagTAA**tgcctttcttgccattgtgtgctctagagggccctattctatagtgtcacctaaatgctagagctcgctgatcagcctcgactgtgccttctagttgccagccatctgttgtttgcccctcccccgtgccttccttgaccctggaaggtgccact…..3´

***Fourth.*** Proceed directly to study the secondary structures of the CiPCR primers using a standard software by analyzing the entire sequence (orange + bold-black underlined), and then study the underlined parts shown in orange and black separately (see ***Points for the design and analysis of chimeric primers* below**). At this point, primers can be modified by expanding or shortening them, or by adding/removing nucleotides to introduce deletions or additional sequences, i.e. tags, restriction sites, or by exchanging nucleotides to add mutations for site-directed mutagenesis in these regions, etc. until the required or satisfactory sequence is found. If you add modifications, be sure to maintain the correct frame and keep the 5´and 3´ tails at the appropriate nucleotide length to maintain sufficient hybridization strength with the template to signal priming or stopping of the polymerase (no less than 6 nucleotides). Those modifications will be incorporated along with the dsDNA during CiPCR. Proceed similarly to study the reverse primer sequence (bold-black underlined + cyan), and modify it similarly as desired.

***Fifth.*** Modify these regions in the sequence written in the third point according to the sequences ultimately selected in the fourth point, highlight them as desired, and copy them as shown below.

**Selected sequences to design chimeric primers B:**

For the forward (F) primer: 5´-ccaaccctgaggaaccaatc-acaaccatggtgagcaa**-**3´

For the reverse (R) primer: 5´-**gcatggacgagctgtacaagTAA**tgcctttcttgccattg-3´

***Sixth.*** Use these sequences to directly design the chimeric B primers as in normal PCR: for B_F_ copy the orange-black sequence just as it appears in the fifth point in the direction 5´-3´; for B_R_ write the complementary sequence to the black-cyan sequence shown in the fifth point, then invert it so that it is oriented in the 5´-3´ direction (reverse orientation).

**Sequences of chimeric primers B are as follows:**

B_F_ : 5´-ccaaccctgaggaaccaatc-**acaaccatggtgagcaa-**3´

B_R_ : 5´-caatggcaagaaaggcatta-**cttgtacagctcgtccatgc-**3´

***Seventh.*** Chimeric primers B are now ready to be ordered to amplify your insert. To design the normal PCR to amplify the insert, consider only the bold black region of the chimeric primers B to calculate the temperature for the annealing step. Similarly, for CiPCR, consider only those nucleotides of the B primers that can hybridize with complementary nucleotides in the corresponding homology regions of templates to calculate the temperature for the annealing step. Instead of a fixing temperature you could also consider programing a decreasing gradient (see below).

1. **Points to be considered for the design and analysis of chimeric primers.**

For 3´ regions to prime amplification as in regular PCR:

- Not recommended a T or 3 or more C or G at the 3´-terminal end.
- Check 3´ end complementary between primer pairs and self-complementary to avoid hairpin loops or dimerization (if they are high can reduce primer availability and sensitivity).
- Leave a minimum of 3-6 homologous nucleotides to the DNA template at 3´ ends when introducing mutations with primers.
- If using degenerated primers choose the most 3´ end sequences homologous to highly conserved regions on the template.
- A length between 12-20 bp is reasonable. However, to amplify from a DNA template 18 is optimal, while to amplify from a plasmid or a shorter DNA 12-15 will provide sufficient specificity.
- An annealing T 5º below the Tm is usual but sometimes a gradient experiment must be performed for its optimization. Also note that this T could be raised after few cycles when the newly formed products act as templates, which then can anneal with the entire chimeric primer (see also below for considering a T gradient for the annealing stage).
- The concentration may vary from 0.1 to 0.5 µM, but is normal to start with 0.2 µM of each primer.

In a regular primer the above rules for the 3´ regions do not apply to its 5´ regions. However, in a chimeric primer designed for CiPCR, 5´ regions, which carry the homology to the β-DNA, must be similarly checked (be aware that in the amplified α-DNA, **a)** the two strands carry those 5´-sequences in one end, and 3´-complementary sequences derived from 5´-regions of the chimeric primers in the opposite end; **b)** both end-sequences of the α-DNA are homologous to the the β-DNA; and **c)** the α-DNA 3´end sequences are responsible for the annealing to the homologous regions in the β-DNA and directly involved in priming CiPCR amplification; and **d)** the α-DNA 5´end sequences are responsible for the annealing to the homologous regions in the β-DNA and directly involved in signaling the stop to the polymerase).

The rules for the 5´regions of chimeric primers are the following:

- Not recommended a A or 3 or more C or G at the 5´-terminal end.
- Check 5´ end complementary between primer pairs and self-complementary to avoid hairpin loops or dimerization.
- Leave a minimum of 3-6 nucleotides at 5´ ends homologous to the DNA template when introducing mutations with primers.
- Use most 5´ end sequences homologous to highly conserved regions on the template if using degenerated primers
- A length between 12-20 bp is reasonable, but for this purpose you can use 5´ ends as large as you wish (even megaprimers as large as thousands bp).
- An annealing T 5º below the Tm is usual but sometimes a gradient experiment must be performed for its optimization. If using long primers the annealing T can be raised and that will increase the probability of the annealing between different templates vs the reannealing between themselves. Remember that CiPCR does not use oligonucleotide primers except for site-directed mutagenesis (SDM). The concentration may vary from equimolar amounts of the two dsDNAs to 5 or more times higher of one of the fragments (we recommend to select the smaller fragment for this purpose, which is usually the one to be inserted).

1. **Some options for CiPCR design or redesign in case or troubleshooting:**

The chemical synthesis of long primers requires a different scale, becoming more expensive. Thus, our first choice to amplify the α-DNA1 is to order chimeric primers fitting in a standard synthetic scale. In most of our experiments they perform well.

In case of failure, an extra pair of outer chimeric primers can be used to reamplify the α-DNA1 to get α-DNA2 with larger homologous regions (as in **Figure 9 and 10**). This second pair of primers must be check again following the same rules as for the first chimeric primers used to amplify α-DNA1.

In case of failure you still have options, such as a second extension of α-DNA2 with a new pair of synthetic chimeric primers to get α-DNA3 with larger homology sites.

Another option to generate large homology overlapping sites is the use of vector-amplified DNA fragments from the flanking regions, with overlapping sequences to the α-DNA1_,_ 2 or 3 (as in Figure 9) targeted to one or to the two ends of the α-DNA, and then run classical OE-PCR to joint then to obtain the corresponding α-DNA (see **Figure 9**).

Other CiPCR items susceptible to change in the protocols are:

- PCR reagents, can also be modified:
  - Mg^2+^ concentrations
  - Addition of DMSO
  - Addition of glycerol
  - Other
- The thermocycle program. The use of gradients can be very useful in CiPCR, particularly when large homology sites are present. In this case the annealing T can be > 90 ºC. Other possiblitlity is that the two homologous sites have different annealing temperature. In these cases, two different annealing temperatures can be programmed, or alternatively, a gradient starting at high as 80-98ºC for the annealing of the larger homologous region, and then decreasing to allow the annealing of the shorter homologous regions. The gradient could be even different for sets of cycles.
- Try different polymerases. Takara HS, Phusion, Pfu are performing well, but other polymerases can be tested.

**Supplementary Figure 1**

**A**


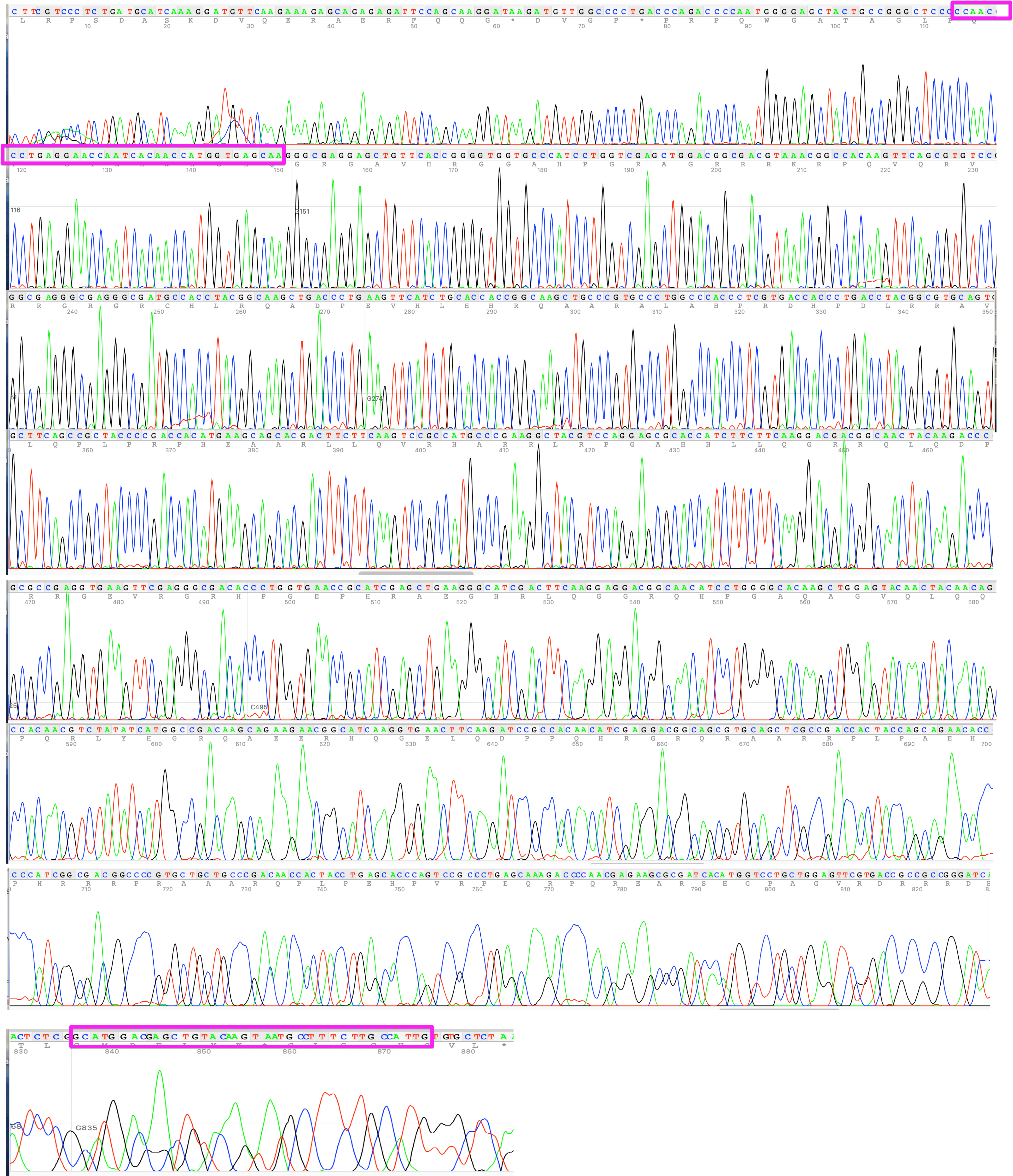


**B**

**
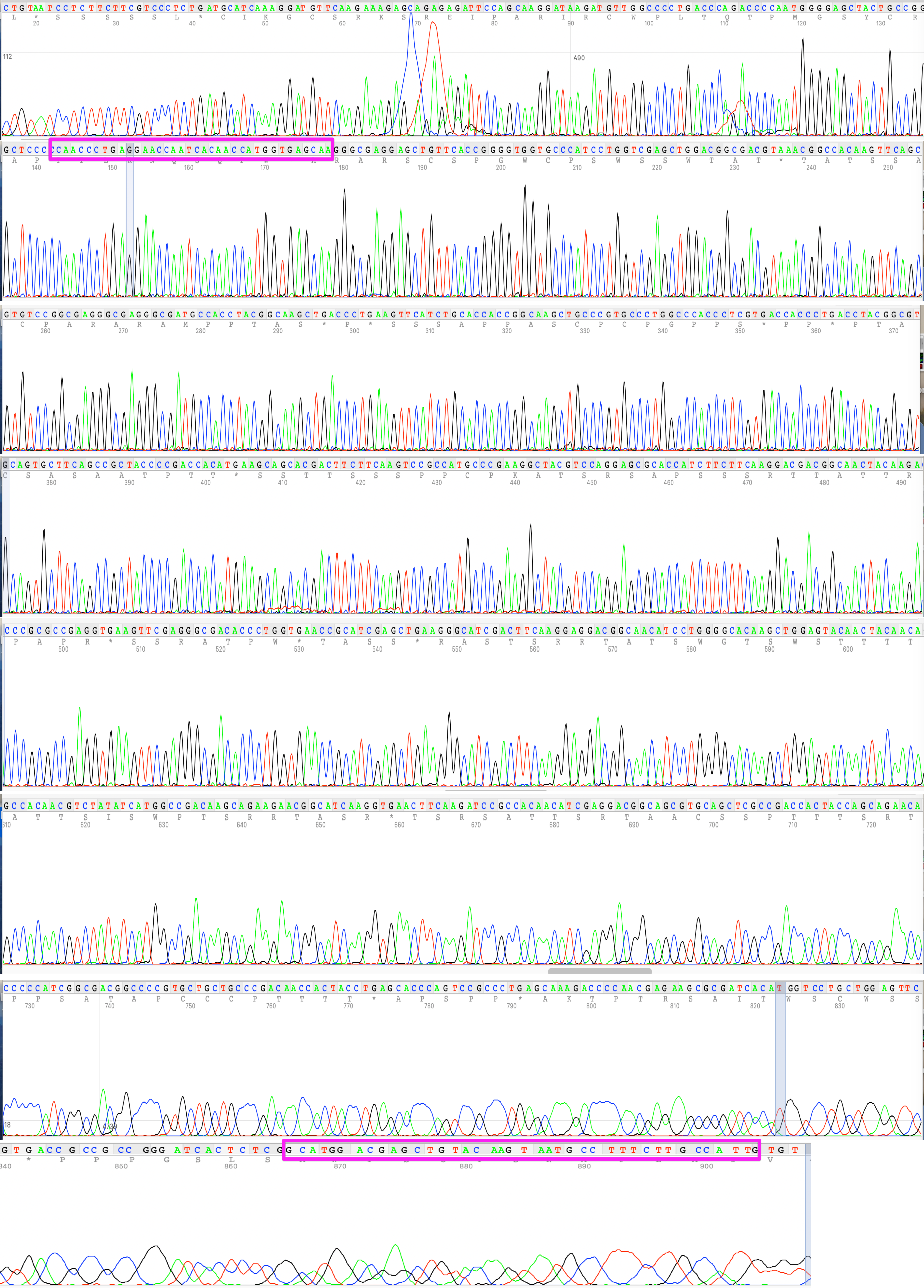
**

**C**

CLUSTAL O(1.2.4) multiple sequence alignment

**B** -CGTAATCCTCTTCTTCGTCCCTCTGATGCATCAAAGGATGTTCAAGAAAGAGCAGAGAG 59

**E**  CTGTAATCCTCTTCTTCGTCCCTCTGATGCATCAAAGGATGTTCAAGAAAGAGCAGAGAG 60

**A** -------------CTTCGTCCCTCTGATGCATCAAAGGATGTTCAAGAAAGAGCAGAGAG 47

***********************************************

B ATTCCAGCAAGGATAAGATGTTGGCCCCTGACCCAGACCCCAATGGGGAGCTACTGCCGG 119

E ATTCCAGCAAGGATAAGATGTTGGCCCCTGACCCAGACCCCAATGGGGAGCTACTGCCGG 120

A ATTCCAGCAAGGATAAGATGTTGGCCCCTGACCCAGACCCCAATGGGGAGCTACTGCCGG 107

************************************************************

B GCTCCCCCAACCCTGAGGAACCAATCACAACCATGGTGAGCAAGGGCGAGGAGCTGTTCA 179

E GCTCCCCCAACCCTGAGGAACCAATCACAACCATGGTGAGCAAGGGCGAGGAGCTGTTCA 180

A GCTCCCCCAACCCTGAGGAACCAATCACAACCATGGTGAGCAAGGGCGAGGAGCTGTTCA 167

************************************************************

B CCGGGGTGGTGCCCATCCTGGTCGAGCTGGACGGCGACGTAAACGGCCACAAGTTCAGCG 239

E CCGGGGTGGTGCCCATCCTGGTCGAGCTGGACGGCGACGTAAACGGCCACAAGTTCAGCG 240

A CCGGGGTGGTGCCCATCCTGGTCGAGCTGGACGGCGACGTAAACGGCCACAAGTTCAGCG 227

************************************************************

B TGTCCGGCGAGGGCGAGGGCGATGCCACCTACGGCAAGCTGACCCTGAAGTTCATCTGCA 299

E TGTCCGGCGAGGGCGAGGGCGATGCCACCTACGGCAAGCTGACCCTGAAGTTCATCTGCA 300

A TGTCCGGCGAGGGCGAGGGCGATGCCACCTACGGCAAGCTGACCCTGAAGTTCATCTGCA 287

************************************************************

B CCACCGGCAAGCTGCCCGTGCCCTGGCCCACCCTCGTGACCACCCTGACCTACGGCGTGC 359

E CCACCGGCAAGCTGCCCGTGCCCTGGCCCACCCTCGTGACCACCCTGACCTACGGCGTGC 360

A CCACCGGCAAGCTGCCCGTGCCCTGGCCCACCCTCGTGACCACCCTGACCTACGGCGTGC 347

************************************************************

B AGTGCTTCAGCCGCTACCCCGACCACATGAAGCAGCACGACTTCTTCAAGTCCGCCATGC 419

E AGTGCTTCAGCCGCTACCCCGACCACATGAAGCAGCACGACTTCTTCAAGTCCGCCATGC 420

A AGTGCTTCAGCCGCTACCCCGACCACATGAAGCAGCACGACTTCTTCAAGTCCGCCATGC 407

************************************************************

B CCGAAGGCTACGTCCAGGAGCGCACCATCTTCTTCAAGGACGACGGCAACTACAAGACCC 479

E CCGAAGGCTACGTCCAGGAGCGCACCATCTTCTTCAAGGACGACGGCAACTACAAGACCC 480

A CCGAAGGCTACGTCCAGGAGCGCACCATCTTCTTCAAGGACGACGGCAACTACAAGACCC 467

************************************************************

B GCGCCGAGGTGAAGTTCGAGGGCGACACCCTGGTGAACCGCATCGAGCTGAAGGGCATCG 539

E GCGCCGAGGTGAAGTTCGAGGGCGACACCCTGGTGAACCGCATCGAGCTGAAGGGCATCG 540

A GCGCCGAGGTGAAGTTCGAGGGCGACACCCTGGTGAACCGCATCGAGCTGAAGGGCATCG 527

************************************************************

B ACTTCAAGGAGGACGGCAACATCCTGGGGCACAAGCTGGAGTACAACTACAACAGCCACA 599

E ACTTCAAGGAGGACGGCAACATCCTGGGGCACAAGCTGGAGTACAACTACAACAGCCACA 600

A ACTTCAAGGAGGACGGCAACATCCTGGGGCACAAGCTGGAGTACAACTACAACAGCCACA 587

************************************************************

B ACGTCTATATCATGGCCGACAAGCAGAAGAACGGCATCAAGGTGAACTTCAAGATCCGCC 659

E ACGTCTATATCATGGCCGACAAGCAGAAGAACGGCATCAAGGTGAACTTCAAGATCCGCC 660

A ACGTCTATATCATGGCCGACAAGCAGAAGAACGGCATCAAGGTGAACTTCAAGATCCGCC 647

************************************************************

B ACAACATCGAGGACGGCAGCGTGCAGCTCGCCGACCACTACCAGCAGAACACCCCCATCG 719

E ACAACATCGAGGACGGCAGCGTGCAGCTCGCCGACCACTACCAGCAGAACACCCCCATCG 720

A ACAACATCGAGGACGGCAGCGTGCAGCTCGCCGACCACTACCAGCAGAACACCCCCATCG 707

************************************************************

B GCGACGGCCCCGTGCTGCTGCCCGACAACCACTACCTGAGCACCCAGTCCGCCCTGAGCA 779

E GCGACGGCCCCGTGCTGCTGCCCGACAACCACTACCTGAGCACCCAGTCCGCCCTGAGCA 780

A GCGACGGCCCCGTGCTGCTGCCCGACAACCACTACCTGAGCACCCAGTCCGCCCTGAGCA 767

************************************************************

B AAGACCCCAACGAGAAGCGCGATCACATGGTCCTGCTGGAGTTCGTGACCGCCGCCGGGA 839

E AAGACCCCAACGAGAAGCGCGATCACATGGTCCTGCTGGAGTTCGTGACCGCCGCCGGGA 840

A AAGACCCCAACGAGAAGCGCGATCACATGGTCCTGCTGGAGTTCGTGACCGCCGCCGGGA 827

************************************************************

B TCACTCTCGGCATGGACGAGCTGTACAAGTAATGCCTTTCTTGCCATTGTGT-------- 891

E TCACTCTCGGCATGGACGAGCTGTACAAGTAATGCCTTTCTTGCCATTGTGTGCTCTAGA 900

A TCACTCTCGGCATGGACGAGCTGTACAAGTAATGCCTTTCTTGCCATTGTGTGCTCTA-- 885

****************************************************

**Supplementary Fig. 1**. Electropherograms showing the complete coding sequence of EGFP (717 bp) of the two identical constructs pcDNA3-SLC16A2(wt)-EGFP, obtained by Type I and Type II CiPCR (corresponding to the insertion of EGFP into pcDNA3-SLC16A2(wt) shown in Figure 4, main text), and the corresponding 5´ and 3´ flanking regions with SLC16A2 and pcDNA3 respectively. **A**. Sequence of the 5´-3´ strand of the construct obtained by Type I CiPCR. **B**. Sequence of the 5´-3´ strand of the construct obtained by Type II CiPCR**. C**. Shows the alignment of sequences from A and B with the expected sequence **E** (correspondent to the complete coding sequence of EGFP (717 bp) and 5´ SLC16A2 and 3´ pcDNA3 flanking regions:

Sequence A: Type I CiPCR I, 5´-3´strand 885 bp

Sequence B: Type II CiPCR, 5´-3´strand 892 bp

Sequence E: Expected sequence 900 bp

Pink squares (in **A** and **B**) and pink characters (in **A,** **B** and **C)** indicate the chimeric primers location and their correct insertion in the construct pcDNA3-SLC16A2(wt)-EGFP. In **C**, the upstream sequence, which belongs to the end part of hSLC16A2, in highlighted in yellow. The stop codon TAA is underlined. The downstream sequence following EGFP, which belongs to the pcDNA3 vector-backbone in highlighted in cyan. 100% homology is observed in the three sequences.

**
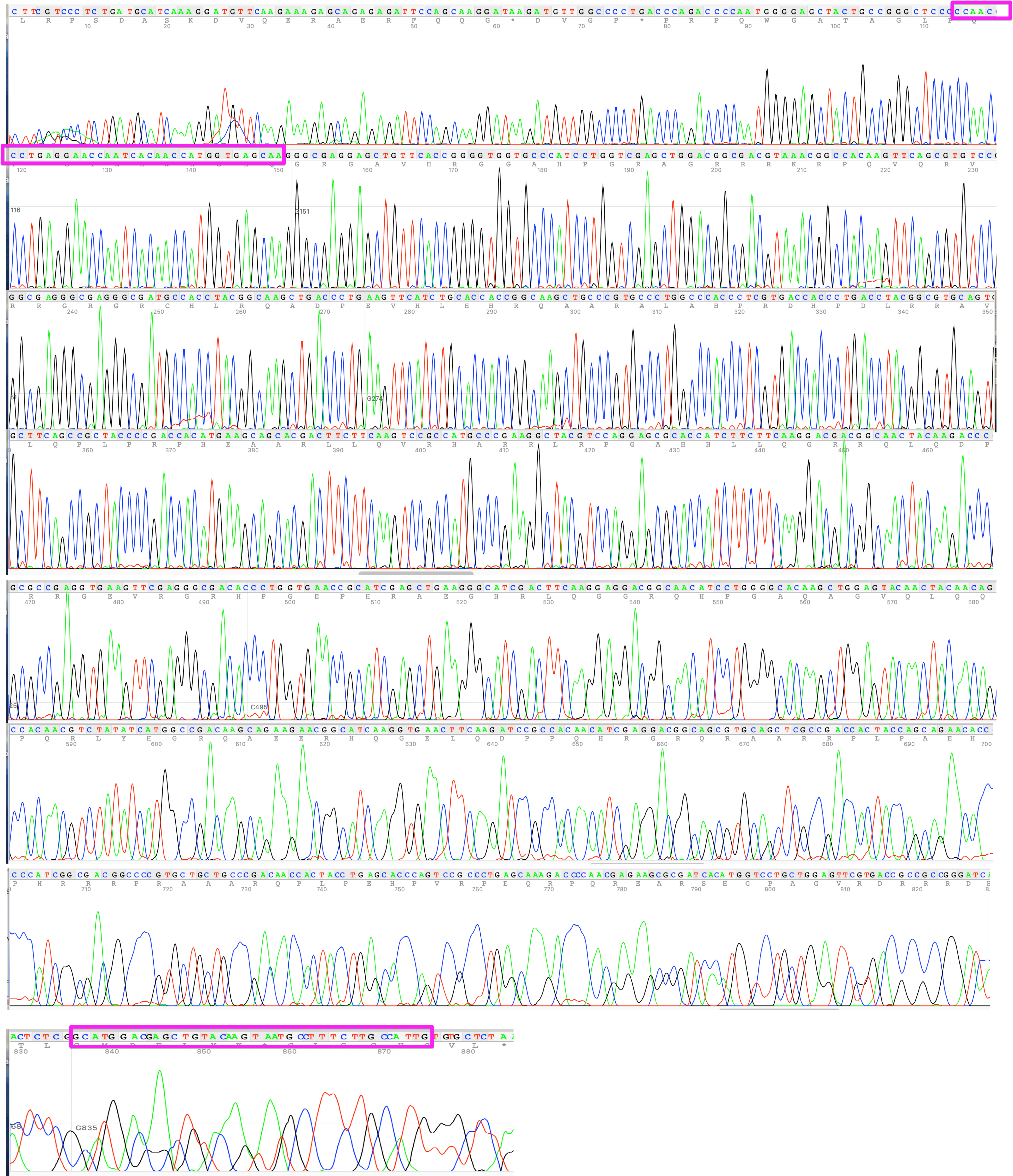

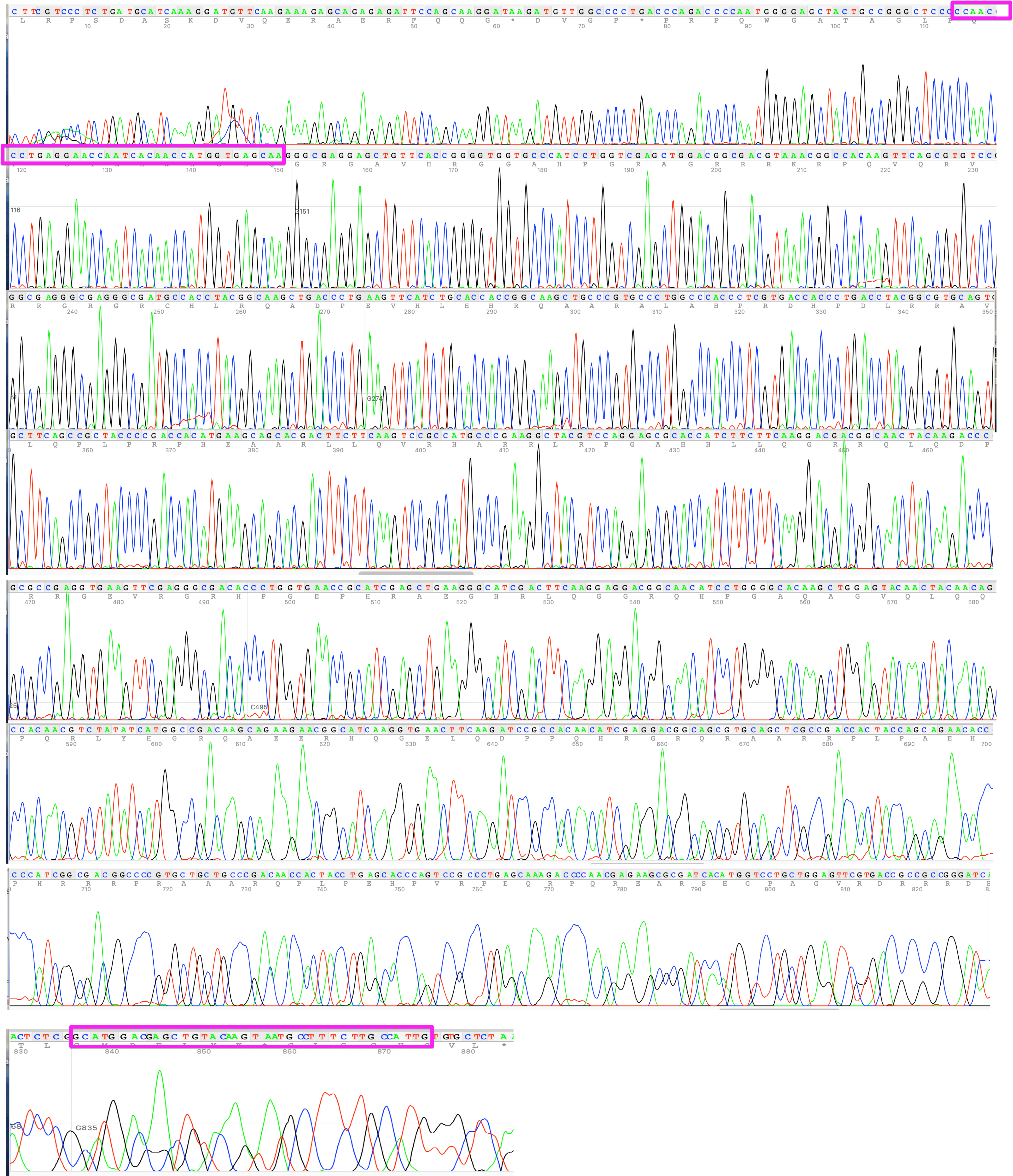
Supplementary Figure 2**

**Type II CiPCR**

**Sequence 5´-3´ pcDNA3-SLC16A2(wt)-EGFP**

**Pink squares indicate the chimeric primes**

**Type II CiPCR**

**Sequence 5´-3´ pcDNA3-SLC16A2(wt)-EGFP**

**Pink squares indicate the chimeric primes**


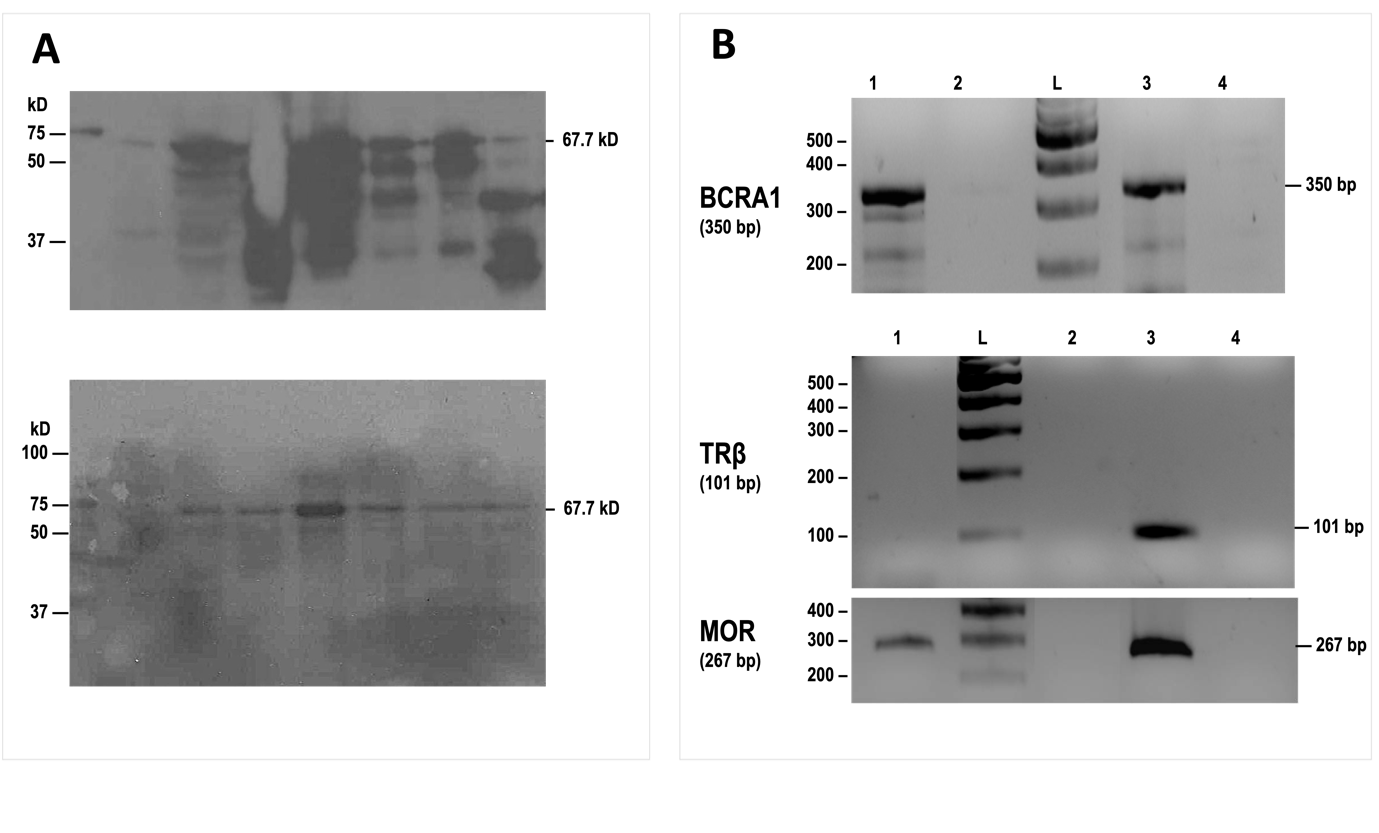


**
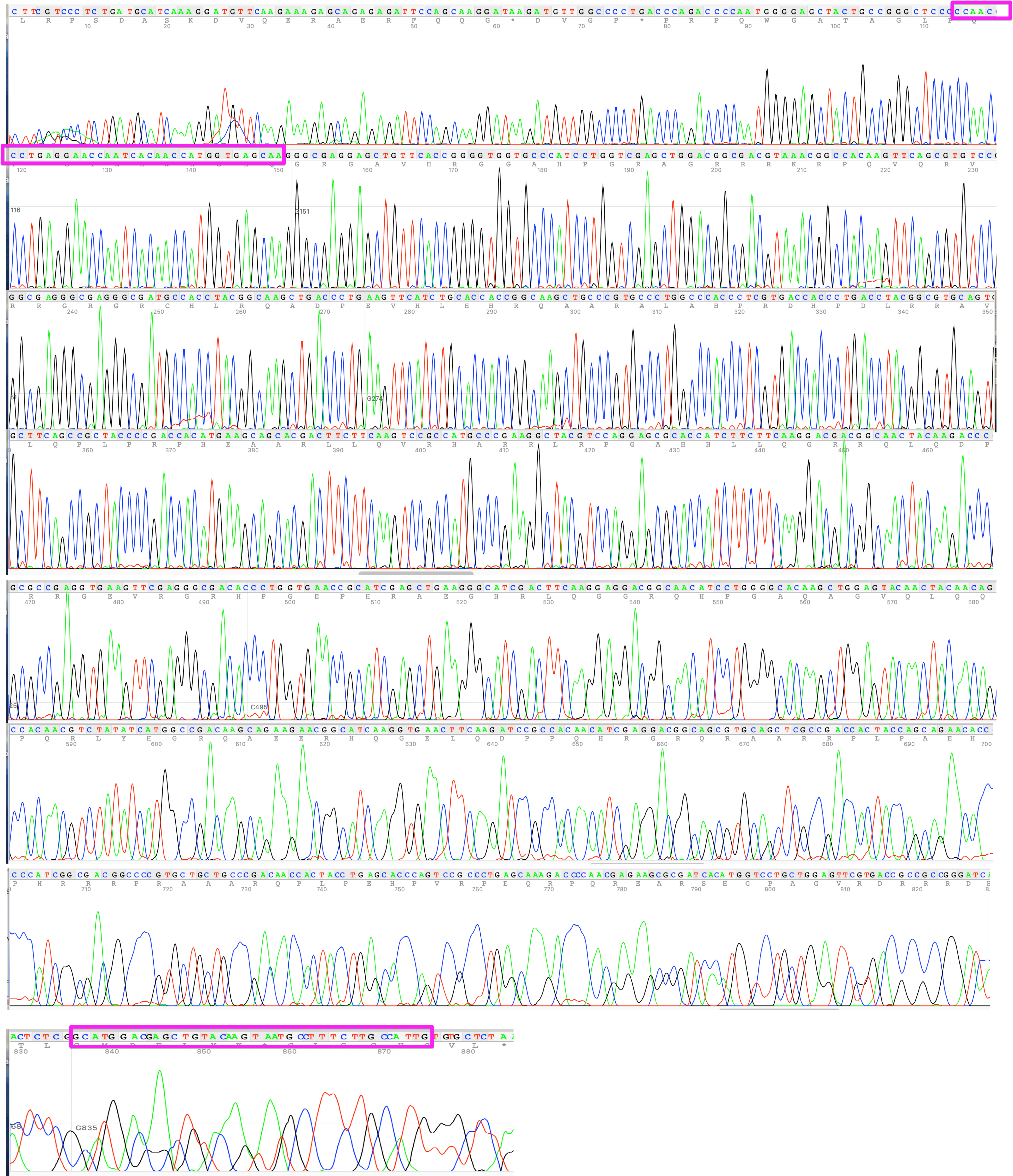

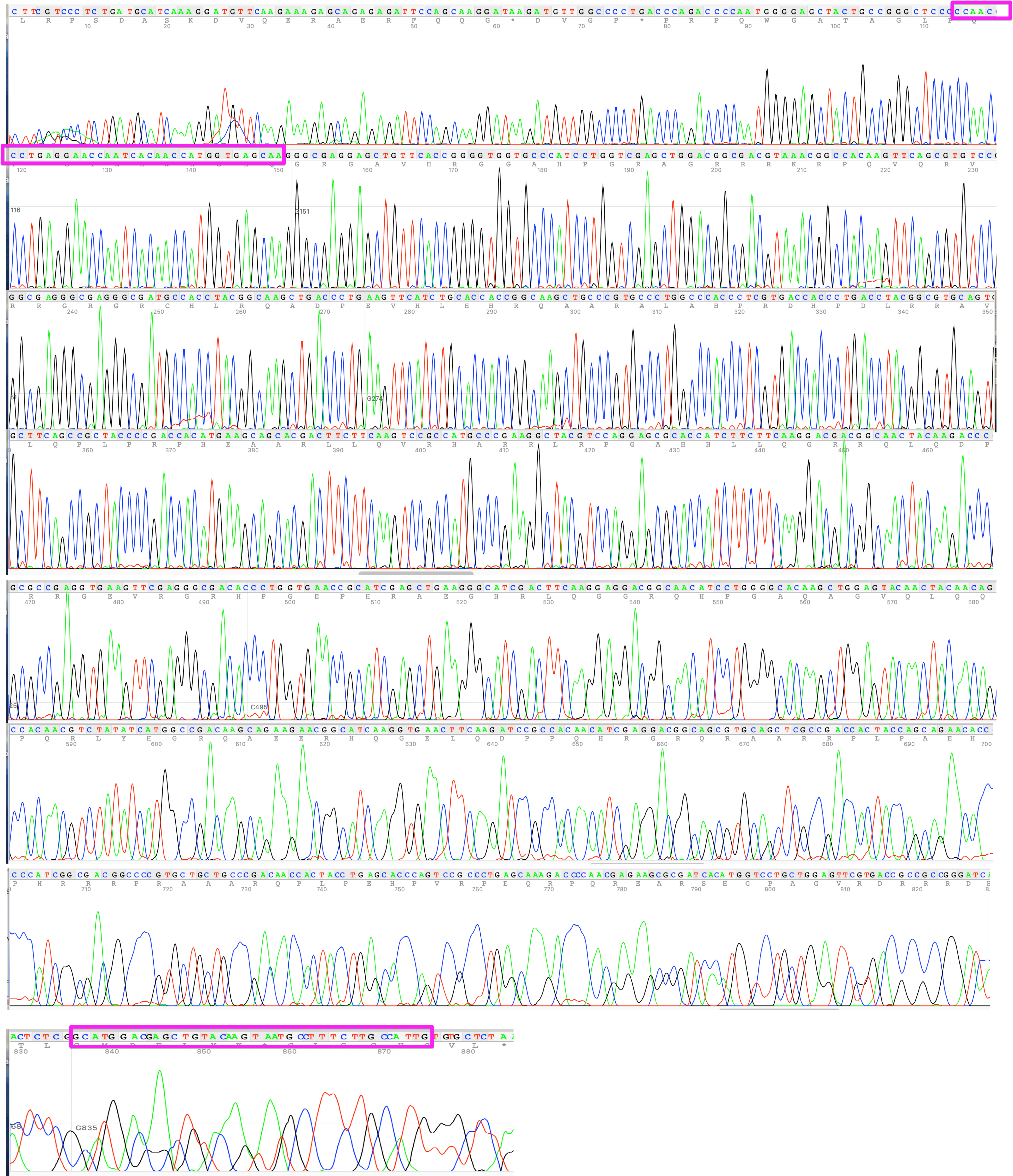
Supplementary Fig. 2. A**. Uncropped immunoblots from Figure 6E. **B**. Uncropped agarose gels from Figure 6H.

**Type II CiPCR**

**Sequence 5´-3´ pcDNA3-SLC16A2(wt)-EGFP**

**Pink squares indicate the chimeric primes**

**Type II CiPCR**

**Sequence 5´-3´ pcDNA3-SLC16A2(wt)-EGFP**

**Pink squares indicate the chimeric primes**
